# Microbiome analysis and confocal microscopy of used kitchen sponges reveal massive colonization by *Acinetobacter*, *Moraxella* and *Chryseobacterium* species

**DOI:** 10.1038/s41598-017-06055-9

**Published:** 2017-07-19

**Authors:** Massimiliano Cardinale, Dominik Kaiser, Tillmann Lueders, Sylvia Schnell, Markus Egert

**Affiliations:** 1 Institute of Applied Microbiology, Research Center for BioSystems, Land Use, and Nutrition (IFZ), Justus–Liebig–University Giessen, Giessen, Germany; 2 Faculty of Medical and Life Sciences, Institute of Precision Medicine (IPM), Microbiology and Hygiene Group, Furtwangen University, Villingen-Schwenningen, Germany; 3 Institute of Groundwater Ecology, Helmholtz Zentrum München - German Research Center for Environmental Health, Neuherberg, Germany

## Abstract

The built environment (BE) and in particular kitchen environments harbor a remarkable microbial diversity, including pathogens. We analyzed the bacterial microbiome of used kitchen sponges by 454–pyrosequencing of 16S rRNA genes and fluorescence *in situ* hybridization coupled with confocal laser scanning microscopy (FISH–CLSM). Pyrosequencing showed a relative dominance of *Gammaproteobacteria* within the sponge microbiota. Five of the ten most abundant OTUs were closely related to risk group 2 (RG2) species, previously detected in the BE and kitchen microbiome. Regular cleaning of sponges, indicated by their users, significantly affected the microbiome structure. Two of the ten dominant OTUs, closely related to the RG2-species *Chryseobacterium hominis* and *Moraxella osloensis*, showed significantly greater proportions in regularly sanitized sponges, thereby questioning such sanitation methods in a long term perspective. FISH–CLSM showed an ubiquitous distribution of bacteria within the sponge tissue, concentrating in internal cavities and on sponge surfaces, where biofilm–like structures occurred. Image analysis showed local densities of up to 5.4 * 10^10^ cells per cm^3^, and confirmed the dominance of *Gammaproteobacteria*. Our study stresses and visualizes the role of kitchen sponges as microbiological hot spots in the BE, with the capability to collect and spread bacteria with a probable pathogenic potential.

## Introduction

In industrialized countries, humans spend up to 90% of their lifetime within built environments (BEs)^1^. BEs harbor a huge variety of microhabitats that are colonized by a wealth of microbial species, which occupied, or evolved to adapt to, the available niches^2, 3^. These microorganisms form the so called BE microbiome^2–8^, whose variability reflects the heterogeneity of the BE environment, ranging from simple huts in rural villages to very confined and extreme habitats, such as intensive care units and cleanrooms^9^. Due to continuous mutual interactions, the BE microbiome is suspected to have a significant impact on health and well–being of the human occupants, which probably goes beyond classical infectious diseases, such as food–borne illnesses; however, the details are far from being fully understood^10, 11^.

**Figure 1:**
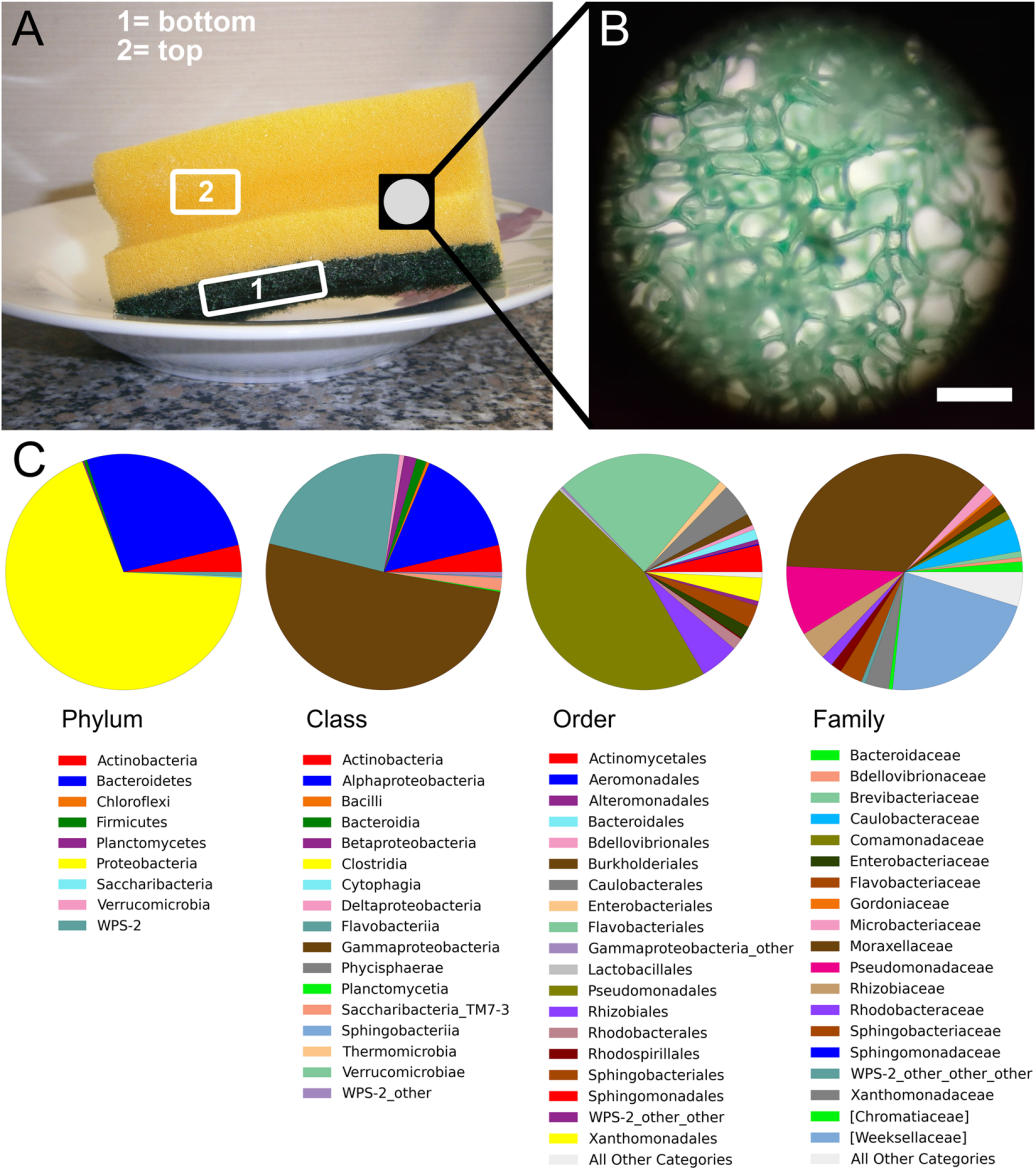
(**A**) Kitchen sponges, due to their porous nature (evident under the binocular; (**B**)) and water-soaking capacity, represent ideal incubators for microorganisms. Scale bar (**B**): 1 mm. (**C**) Pie charts showing the taxonomic composition of the bacterial kitchen sponge microbiome, as delivered by pyrosequencing of 16S rRNA gene libraries of 28 sponge samples (top and bottom samples of 14 sponges, respectively). For better readability, only the 20 most abundant orders and families are listed.

Within a domestic environment, kitchens and bathrooms have a high potential to function as “microbial incubators”, due to the continuous inoculation of new microbial cells, e.g. by food handling and direct body contact to the domestic surfaces; the colonization success of these microbes then depends on the suitability of the environmental conditions, such as humidity and nutrient availability. Despite common misconception, it was demonstrated that kitchen environments host more microbes than toilets^12–14^. This was mainly due to the contribution of kitchen sponges (Fig. 1A, B), which were proven to represent the biggest reservoirs of active bacteria in the whole house^13, 14^. Ojima and coworkers (2002)^11^ showed that kitchen sponges had the second highest load of coliforms of the whole house, after the drain traps. Further works showed the presence of specific pathogenic bacteria in kitchen sponges, including *Campylobacter* spp.^15^, *Enterobacter cloacae* ^16, 17^, *Escherichia coli* ^14, 17, 18^, *Klebsiella* spp.^14, 16–18^, *Proteus* spp.^17^, *Salmonella* spp.^19^, and *Staphylococcus* spp.^14, 16, 17^. This evidence, as well as the common perception of kitchen sponges as collectors of microorganisms, initiated the development of commercial products and devices for effective sanitation of kitchen sponges (for example, Martz (2001)^20^). In addition, microwave and boiling treatments were shown to significantly reduce the bacterial load^19, 21^. However, results were contradictory, for example showing effectiveness in the laboratory, but not in used kitchen sponges^18^, and no method alone seemed to be able to achieve a general bacterial reduction of more than about 60%^22^. Kitchen sponges not only act as reservoir of microorganisms, but also as disseminators over domestic surfaces, which can lead to cross–contamination of hands and food, which is considered a main cause of food–borne disease outbreaks^23–29^. Studies performed so far on kitchen sponges have largely used cultivation–dependent approaches, often focusing on specific microbial target groups. Often, growth on selective media was not further confirmed, for example, by 16S rRNA gene sequencing. Thus, information is currently lacking concerning the overall composition of the kitchen sponge microbiome on a molecular, i.e. cultivation–independent level. To the best of our knowledge, the kitchen sponge microbiome was investigated by next generation sequencing only in a single study so far, in which, however, the results were stemming from just a single sponge sample, analyzed among 82 other kitchen surfaces^30^.

In this study, we comprehensively analyzed the bacterial microbiome of used, domestic kitchen sponges by high–throughput 16S rRNA gene sequencing, in order to unravel its actual taxonomic assemblage and diversity, and to assess the influence of selected intrinsic (sponge–specific) and extrinsic (sponge usage–specific) factors (Supplementary Table S1) on the microbiome structure. In addition, we aimed to estimate the pathogenic potential of the sponge microbiota. Finally, we explored the spatial distribution pattern of bacteria in the kitchen sponge tissue by 3D–microscopy using fluorescence *in situ* hybridization in combination with confocal laser–scanning microscopy (FISH–CLSM), to complement and validate the sequencing data, and to assess *in situ* abundance of bacteria. Our work closes a gap in the knowledge of the BE microbiome, and provides new and important information for an effective domestic hygiene awareness.

## Results

The aim of this study was to investigate the bacterial microbiome composition of used kitchen sponges, to visualize the spatial distribution of the bacteria within the sponge tissue and to identify factors that influence the microbiome composition. Notably, no bacteria could be detected in a collection of newly bought, i.e. unused kitchen sponges, using 16S rRNA gene PCR, FISH or cultivation (Supplementary Figures S1 and S2). Although new kitchen sponges are probably not sterile, the data presented below obviously result from a bacterial colonization that largely took place during the use of the sponges.

### Pyrosequencing of 16S rRNA gene amplicon libraries and phylogenetic analysis

A total of 223,741 raw sequences were obtained by 454–pyrosequencing of 16S rRNA gene amplicon libraries from 28 sponge samples, stemming from 14 used kitchen sponges. After length/quality filtering, a total of 33,181 high quality sequences were kept (Supplementary log File 1), which were then grouped into 4014 OTUs at 97% sequence similarity threshold. After removal of chimeric OTUs (1371), singleton OTUs (2218) and further plastidic OTUs (63), 362 bacterial OTUs remained, representing a total of 27,473 sequences (357–1418 sequences per sample, with an average of 981.2 reads per sample) (Supplementary Figure S3). No mitochondrial OTUs occurred. The sequence dataset was submitted to EMBL (www.ebi.ac.uk/ena) under the project number PRJEB18578.

**Figure 2:**
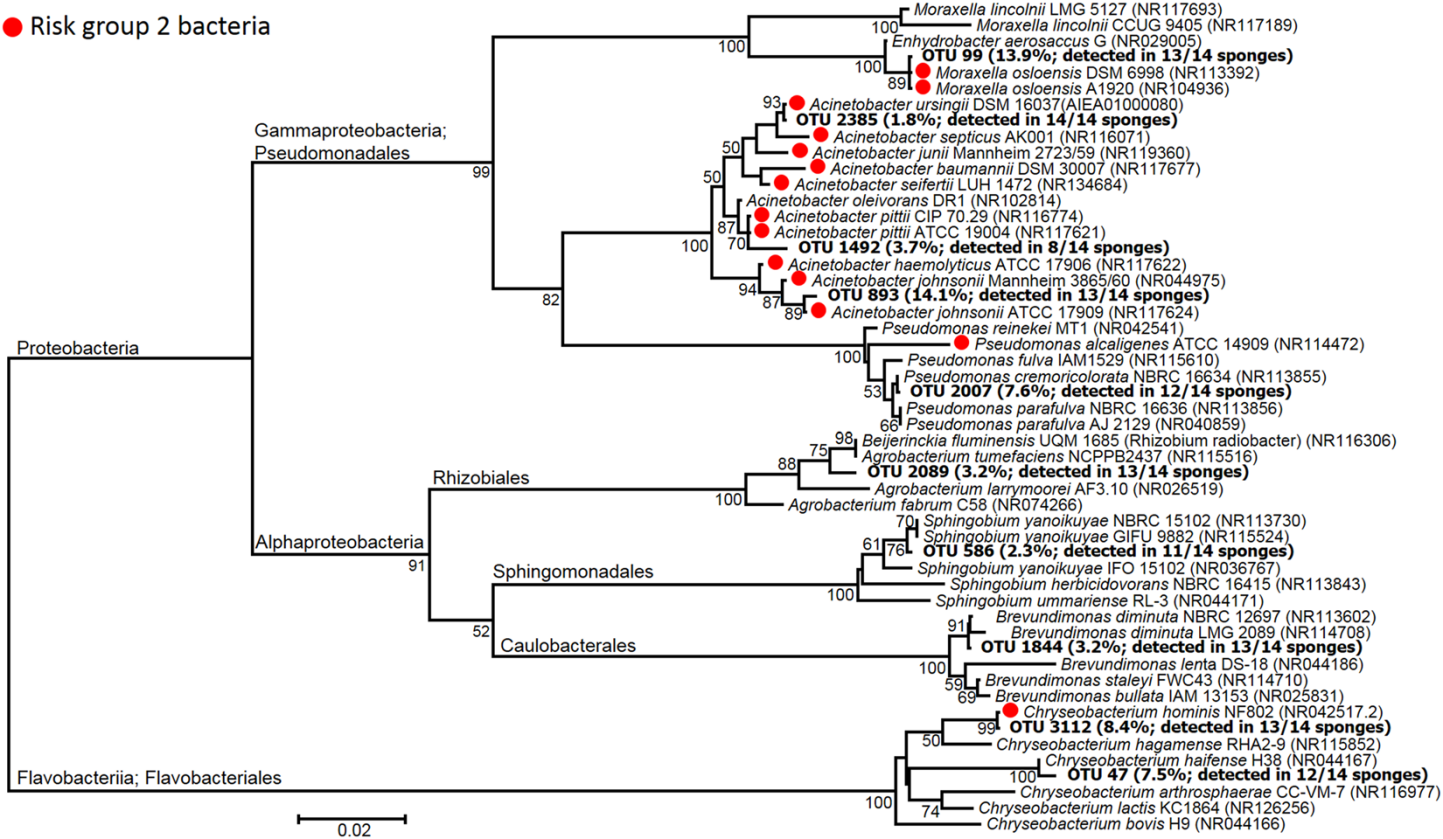
Neighbor-joining phylogenetic tree of the ten most abundant OTUs in the analyzed kitchen sponges, as retrieved by pyrosequencing of 16S rRNA gene amplicon libraries. The relative abundance (percentage of the total sequence dataset) and the detection frequency (number of sponges where they were detected) are given in parenthesis after the OTU number. The most similar reference sequences retrieved by BLAST and EzTaxon alignment (type strains only) were included in the tree, followed by the corresponding accession numbers. Red circles indicate risk group 2 organisms, according to the German Technical Rule for Biological Agents No. 466 (TRBA 466^31^). Numbers at the nodes indicate percentage values of 1000 bootstrap re–samplings (only percentages ≥ 50 are shown). Scale bar represents substitution rate per nucleotide position.

The taxonomic assignment of the OTUs performed by QIIME according to the RDP database, revealed 9 phyla, 17 classes, 35 orders, 73 families and 118 genera (Fig. 1C). The most dominant phylum was *Proteobacteria* with an average relative abundance of 68.51%, per sample followed by *Bacteroidetes* (26.35%) and *Actinobacteria* (3.69%). The remaining phyla (WPS-2, *Firmicutes*, *Planctomycetes*, *Saccharibacteria*, *Verrucomicrobia* and *Chloroflexi*) accounted for 0.612%, 0.439%, 0.196%, 0.166%, 0.032% and 0.007%, respectively. The class *Gammaproteobatceria* dominated the community with 51.14% relative abundance. *Pseudomonadales* (class *Gammaproteobatceria*) and *Flavobacteriales* (phylum *Bacteroidetes*) were the dominant orders, with 45.64% and 23.21%, respectively. The most abundant families were *Moraxellaceae* and *Pseudomonadaceae* among the *Gammaproteobacteria* (36.04% and 9.58%, respectively), and [*Weeksellaceae*] among the *Flavobacteriales* (21.90%) (Fig. 1C). The ten most abundant OTUs accounted for 68.72% of all sequences and were quite ubiquitous, each occurring in 11 to 14 sponges, with the only exception of OTU1492 that was detected in 8 sponges only. The phylogenetic affiliation of these 10 OTUs delivered by QIIME, based on the RDP database, was not always accurate. In fact, OTU99 (the second most abundant OTU of the whole dataset, accounting for 13.9% of all sequences) was identified as *“Enhydrobacter* sp.*”*. However, manual BLASTn and EzTaxon alignment of the representative sequence yielded “*Moraxella osloensis*” as the closest relative. Therefore, a phylogenetic analysis was conducted to accurately identify the closest relative species, by including the ten most abundant OTUs of our dataset and 42 affiliated 16S rRNA gene sequences retrieved by BLAST and/or EzTaxon alignment (Fig. 2). The phylogenetic tree, based on a multialignment of 494 nucleotide positions, showed that five of the ten most frequently detected OTUs were most closely related to bacteria categorized as risk group (RG) 2 (*Acinetobacter johnsonii, Acinetobacter pittii, Acinetobacter ursingii, Chryseobacterium hominis* and *Moraxella osloensis*), according to the German Technical Rule for Biological Agents 466 (TRBA 466^31^), thereby suggesting some pathogenic potential. The TRBA 466 is issued by the German Federal Institute for Occupational Safety and Health and represents the central German document regarding the risk assessment of microorganisms, i.e. a reliable reference to evaluate the health risk potential of microorganisms. The five OTUs probably representing potentially pathogenic bacteria accounted for 41.9% of the whole sequence dataset. The five remaining OTUs were closely related to the RG 1 (non-pathogenic) species *Brevundimonas diminuta, Chryseobacterium haifense, Pseudomonas cremoricolorata*, *Rhizobium radiobacter* (*Agrobacterium tumefaciens*) and *Sphingobium yanoikuyae* (Fig. 2).

Only seven OTUs were affiliated with *Enterobacteriaceae* and had a cumulative relative abundance of 1.18%. Manual BLAST analysis of these OTUs yielded the genera *Enterobacter*, *Escherichia*, *Citrobacter* and *Leclercia*. Only single OTUs affiliated with *Staphylococcus* and *Streptococcus* were found (0.037% and 0.017% relative abundance, respectively), most closely related to the RG1 species *Staphylococcus succinus* and *Streptococcus thermophilus*, respectively. Further potentially pathogenic taxa previously isolated from kitchen sponges, such as *Salmonella*, *Proteus* and *Campylobacter*, were not detected.

**Figure 3:**
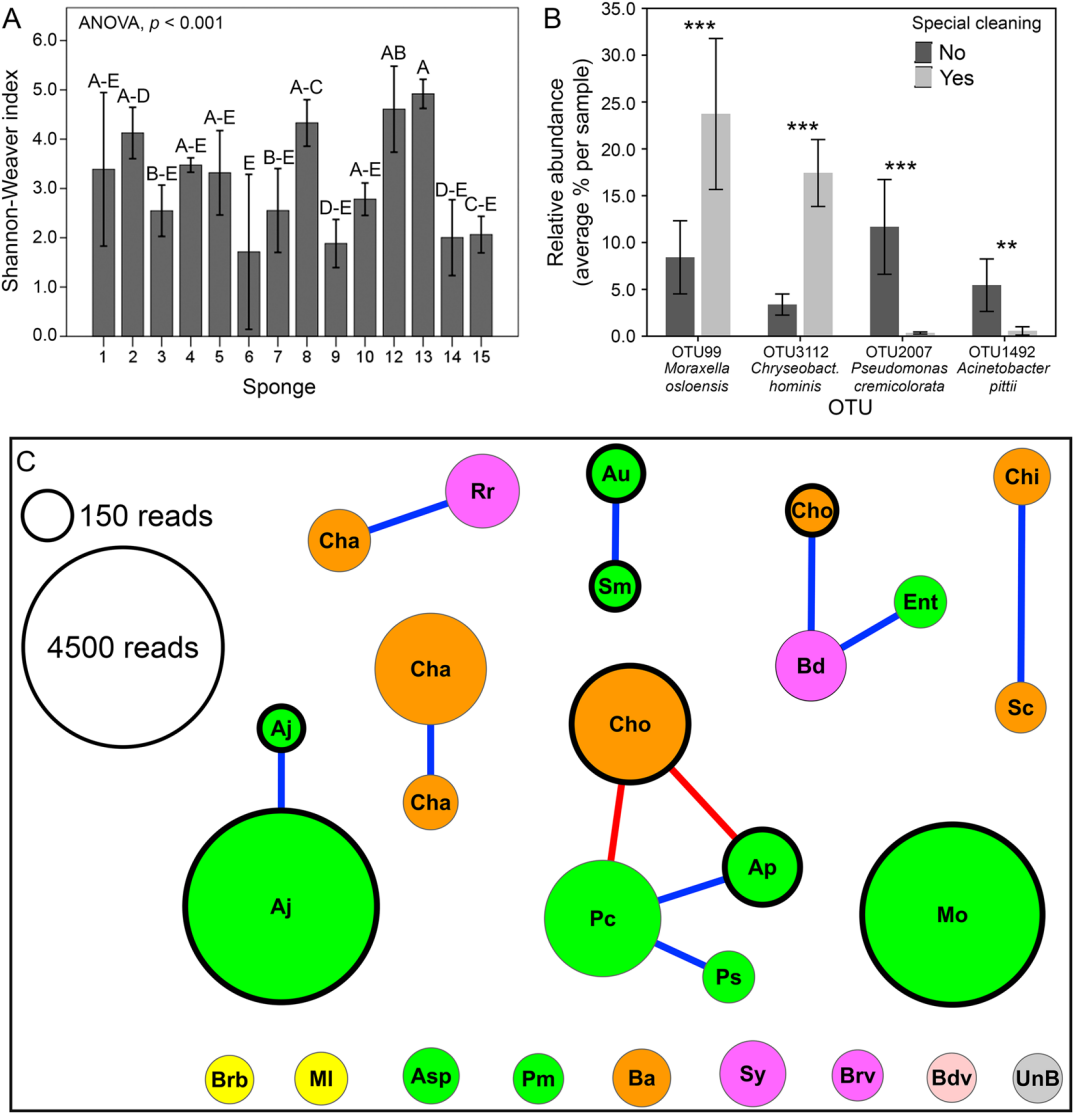
(**A**) Shannon–Weaver index, as calculated on the rarefied dataset (sequence depth: 357 sequences per sample), grouped per individual sponge; bars indicate averages and whiskers indicate ± 2 standard error; different letters indicate significantly different means (Tukey’s HSD test at *p* < 0.05). (**B**) Relative abundance of the four OTUs significantly affected by the factor “special cleaning” (G-test of independency, **p < 0.005; ***p < 0.001). OTU identification of the closest relative species, as reported on the X-axis below the OTU number, is according to phylogenetic analysis (see Fig. 2). (**C**) Correlations of occurrence patterns between OTUs of the kitchen sponge microbiome. The network shows correlations between abundant OTUs (>0.5% of the total dataset), generated at 97% similarity level. Nodes represent OTUs and edges represent strong positive (R > 0.6, *p* < 0.001) or strong negative (R < −0.6, *p* < 0.001) Spearman correlations (blue and red lines, respectively). A thicker node border indicates a close relative to a RG2-species, according to TRBA 466 (see also legend of Fig. 2). OTU abundance (total number of reads) is indicated by node size, as shown in the figure. Node color indicates taxonomic affiliation at phylum/class level: yellow – *Actinobacteria*, purple – *Alphaproteobacteria*, orange – *Bacteroidetes*, pink – *Deltaproteobacteria*, green – *Gammaproteobacteria*, grey – unidentified. Node label indicates the best possible taxonomic identification of the OTUs: Aj – *Acinetobacter johnsonii*, Ap – *Acinetobacter pittii*, Asp – *Acinetobacter* sp., Au – *Acinetobacter ursingii*, Ba – *Bacteroides* sp., Bd – *Brevundimonas diminuta*, Bdv – *Bdellovibrio* sp., Brb - *Brevibacterium* sp., Brv - *Brevundimonas* sp., Cha – *Chryseobacterium haifense*, Chi – *Chryseobacterium hispanicum*, Cho – *Chryseobacterium hominis*, Ent – *Enterobacteriaceae*, Ml – *Microbacterium lacticum*, Mo – *Moraxella oslonensis*, Pc – *Pseudomonas cremoricolorata*, Pm *Pseudoxanthomonas mexicana*, Ps – *Pseudomonas* sp., Rr – *Rhizobium radiobacter*, Sc – *Sphingobacterium caeni*, Sm – *Stenotrophomonas maltophilia*, Sy – *Sphingobium yanoikuyae*, UnB – Unidentified bacterium.

Alpha– and beta–diversity metrics were calculated on the rarefied dataset with an even sequencing depth of 357 sequences per sample. Good’s coverage ranged from 92.7% to 99.7%, with an average of 96.7 ± 1.9% (Supplementary Table S2). The Shannon–Weaver diversity index was very variable between samples and ranged from 0.93 to 5.07; the different sponges showed highly significant differences (ANOVA F_13, 14_ = 7.03, *p* < 0.001; partial-η^2^ = 0.87; 1-β = 0.999) (Fig. 3A), whereas no additional factor significantly affected the alpha–diversity of the sponge microbiome (*p* > 0.1). Other alpha–diversity metrics (Simpson index, Simpson reciprocal index, Simpson evenness, dominance, equitability, PD whole tree, Chao1, Number of OTUs) were calculated, and the results were similar to those obtained for the Shannon–Weaver index (*p* < 0.01; Supplementary Table S2), except for Simpson evenness, which was not significantly different between sponges (*p* = 0.08). Simpson reciprocal was the only not-normally distributed variable, therefore its significance was calculated with the Kruskall–Wallis test instead of ANOVA (Supplementary Table S2).

Weighed Unifrac distances were significantly affected by the factor “special cleaning” with a middle effect size (ADONIS, R^2^ = 0.107, *p* = 0.022; Supplementary Figure S4). G–test of independence showed four significantly different OTUs between the two groups (FDR–corrected *p* values between 1.3 * 10^−9^ and 0.0046; Fig. 3B), all of them belonging to the ten most abundant OTUs (OTU 99 – related to *Moraxella osloensis*, OTU 3112 – related to *Chryseobacterium hominis*, OTU 2007 – related to *Pseudomonas cremoricolorata*, and OTU 1492 – related to *Acinetobacter pittii*). Three of these OTUs were most closely related to RG2 bacteria (see Fig. 2); interestingly, two of them (OTU 99 and OTU 3112) were relatively more abundant in sponges that were regularly cleaned with special procedures (Fig. 3B). No further factor affected the beta–diversity of the sponge microbiota (ADONIS, 0.968 > *p* > 0.072).

### Co–occurrence patterns and network analysis

The co–occurrence analysis showed the presence of statistically significant correlations among the abundant OTUs, across all sponge samples (Fig. 3C), with a clustering coefficient of 0.086. Nine positive and two negative correlations were identified, involving 17 of the 27 abundant OTUs analyzed (average number of neighbors: 0.815; network density: 0.031). Both non-pathogenic as well as RG2-related organisms showed significant correlations; interestingly, seven of the nine positive correlations occurred either between two non-pathogenic or between two RG2-related species, while only two correlations occurred between one non-pathogenic and one RG2-related OTU (Fig. 3C). *Pseudomonas cremoricolorata* was the only non-pathogenic bacterium which seemed able to significantly “antagonize” a RG2-related species (*Chyseobacterium hominis*); however, it was positively correlated with another one (*Acinetobacter pittii*). Interestingly, the two most abundant RG2-related species (*Acinetobacter johnsonii* and *Moraxella osloensis*) appeared independent from the other bacterial species, which might be one explanation for their success in massively colonizing the sponges.

### Fluorescence *in situ* hybridization (FISH) analyses

**Figure 4:**
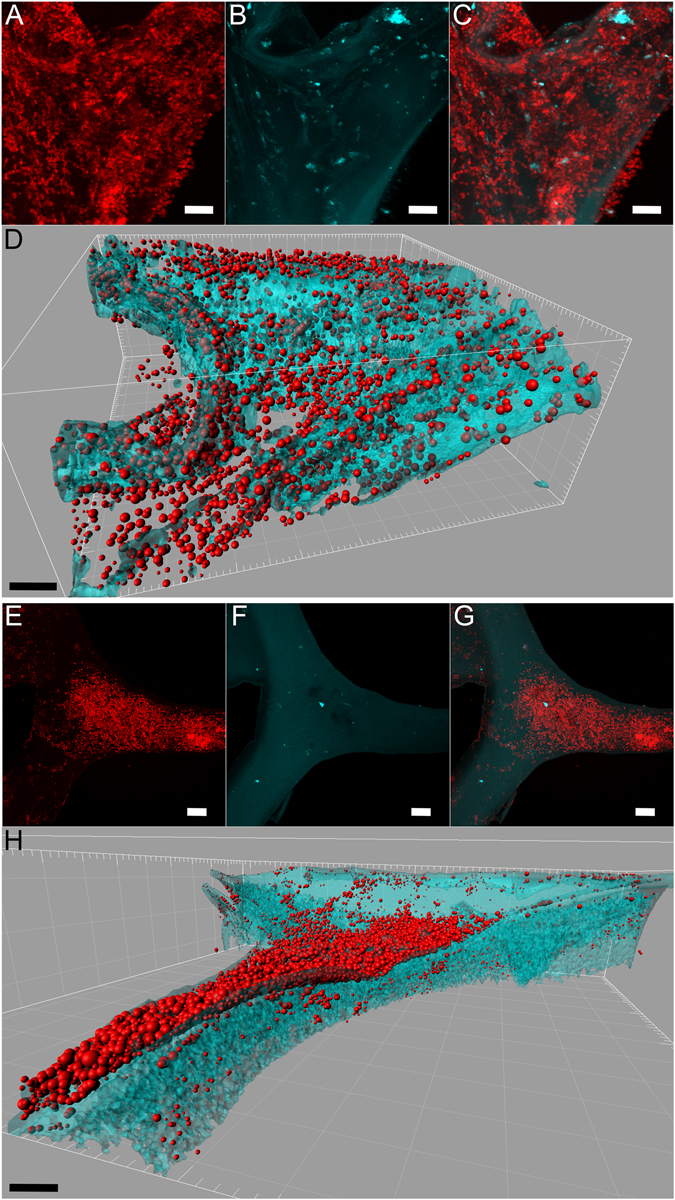
FISH-CLSM analysis of bacteria in sponge sample 9b. Maximum projections of confocal stacks, showing EUB338MIX–stained bacteria in red (**A** and **E**) and sponge autofluorescence in cyan (**B** and **F**); (**C** and **G**) are the overlap of (**A**,** B** and **E**, **F**), respectively. (**D** and **H**) are the three–dimensional models of (**C** and **G**), respectively, where bacteria are converted into size–adjusted spheres and the sponge tissue into semi–transparent iso–surfaces. Scale bars: (**A**–**D**) 10 µm, (﻿**E–H**) 20 µm.

**Figure 5:**
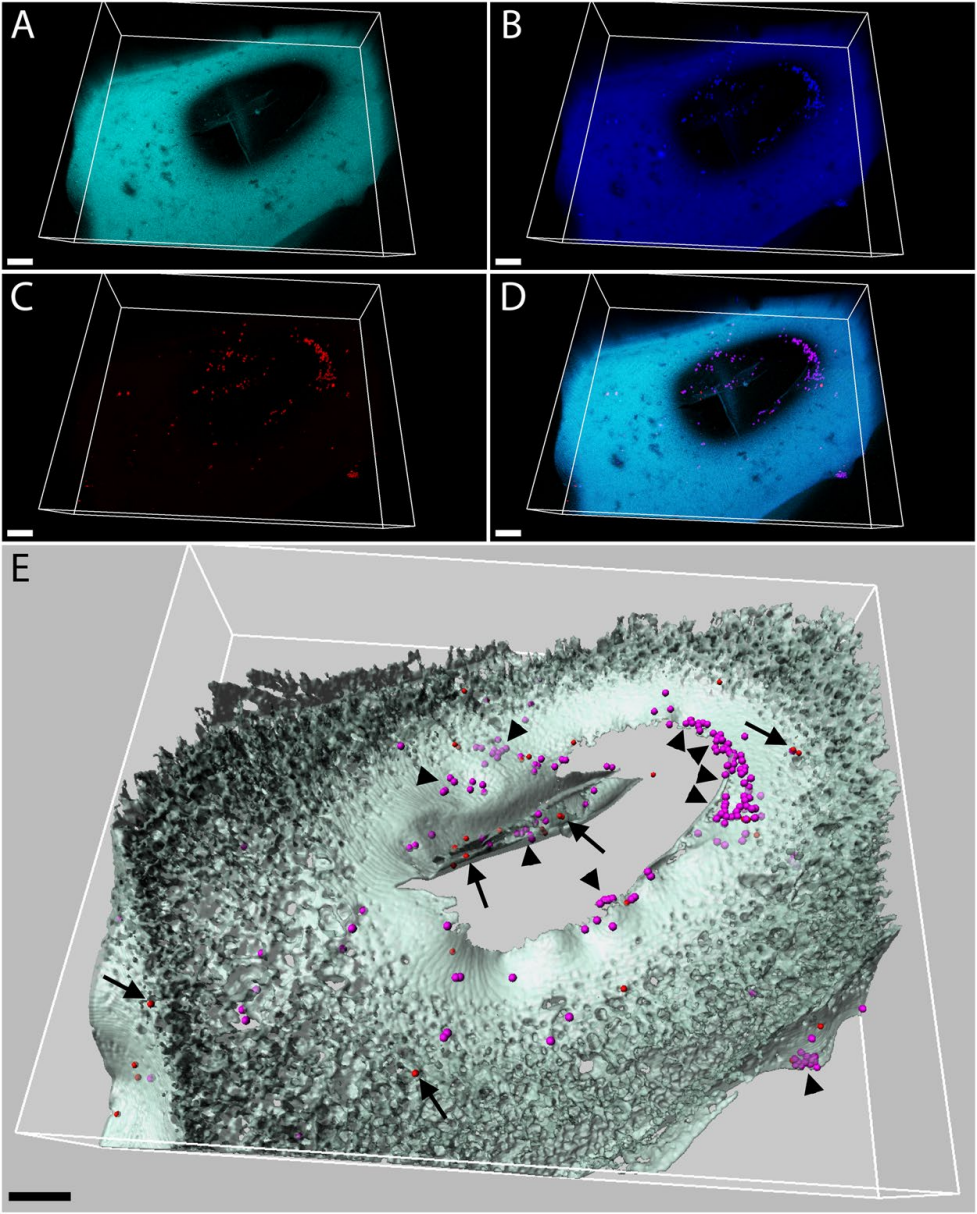
FISH-CLSM analysis of the bacteria in the sponge sample 6b. Volume–renderings of a confocal stack showing sponge autofluorescence (**A**, cyan); (**B**) Gam42a–stained bacteria (**B**, blue) and EUB338MIX–stained bacteria (**C**, red); (**D**) overlap of (**A**–**C**), where *Gammaproteobacteria* appear purple for the overlap of red and blue, while other bacteria remain only red; (**E**) three–dimensional model of (**C**), where bacteria are converted into spheres and the sponge tissue into semi–transparent iso–surfaces; *Gammaproteobacteria* are indicated by arrowheads, while arrows point to other bacteria. Scale bars: 10 µm.

**Figure 6:**
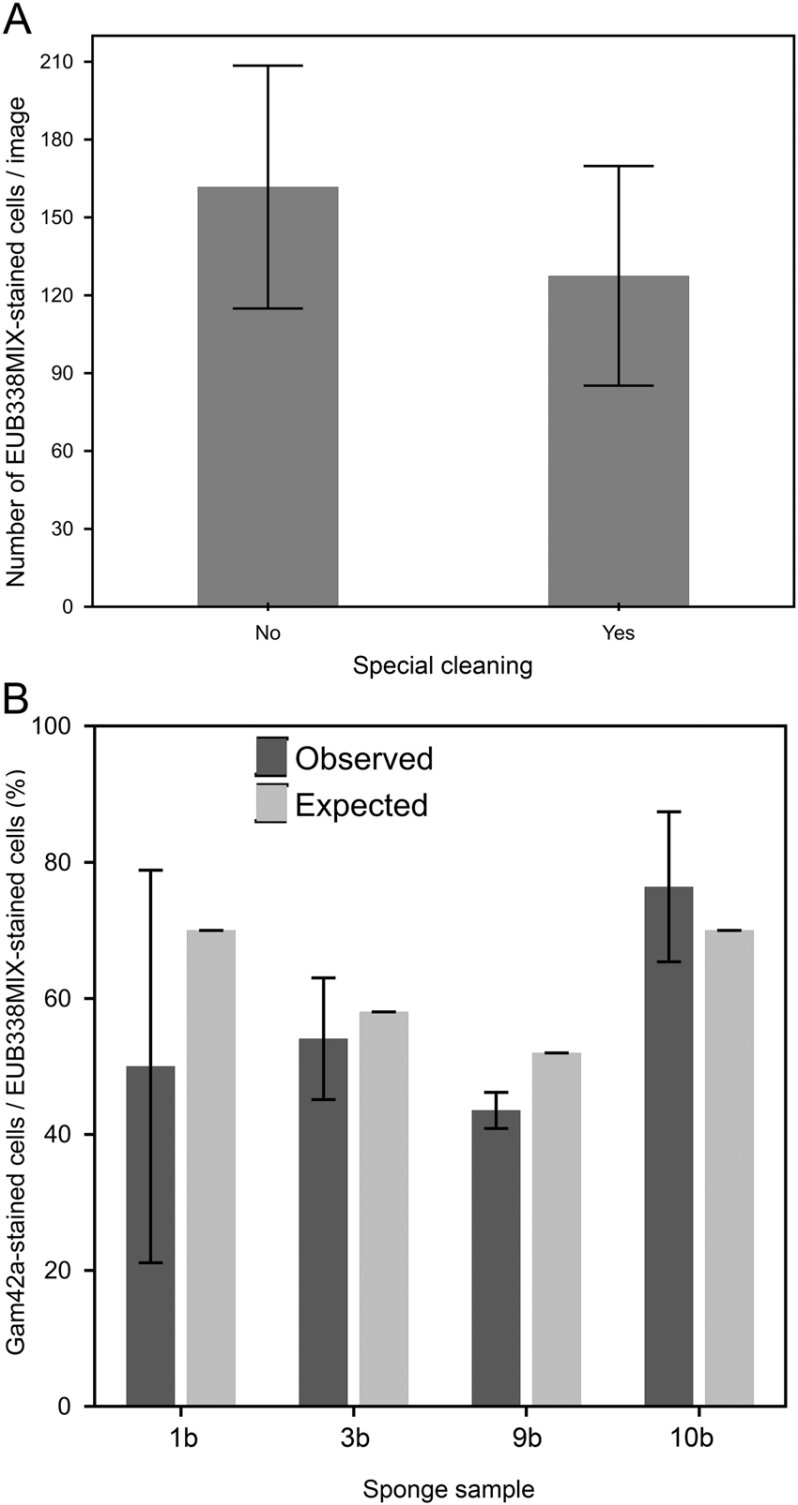
(**A**) Bacterial abundance in selected regularly sanitized (“specially cleaned”) and not sanitized kitchen sponges. Abundance was assessed as number of EUB338MIX-stained cells per microscopy image (N = 14). (**B**) Statistical count of Gam42a–stained cells in selected sponge samples (N = 6–8 microscopy images), as compared to the expected fraction of *Gammaproteobacteria* detected by pyrosequencing analysis of the same samples.

FISH analyses were performed with the aim to i) visualize the spatial distribution of bacteria within the kitchen sponges, ii) determine the local density of bacteria in the sponges, iii) compare the relative bacterial abundances between “specially cleaned” and “non cleaned” sponges, and iv) confirm selected sequencing results. Microscopy images showed that bacterial colonization basically occurred on the surfaces of the sponge tissue (Fig. 4, Supplementary Video File S1), especially on the internal cavity walls (Fig. 5). The intensity of the FISH signals was strong, well above the sponge autofluorescence, and thus indicated high ribosome content in the bacterial cytoplasm, suggesting that the majority of the inspected cells were actively growing. This was further supported by the frequent observation of coupled cells with the typical dividing phenotype. In some cases, the bacterial contamination appeared very massive, reaching local densities of 2.5 * 10^10^ (Fig. 4A–D) and 5.4 * 10^10^ (Fig. 4E–H, Supplementary Video File S1) bacterial cells per cm^3^ of sponge tissue. The specific staining of four sponge samples with the Gam42a FISH–probe resulted in a clearly discriminable signal for gammaproteobacterial cells (Fig. 5B, D–E; arrowheads; Supplementary Figure S5A, C), with respect to the other bacterial cells (Fig. 5C and E, arrows; Supplementary Figure S5B, D). Negative samples stained with the NONEUB probe showed no detectable signal except the sponge tissue autofluorescence (Supplementary Figures S5E–F and S6). Interestingly, regularly sanitized (“specially cleaned”) sponges did not show significantly reduced bacterial numbers (Mann–Whitney U–Test, *p* = 0.78; Fig. 6A).

The class *Gammaproteobacteria* was chosen to confirm the sequencing results by FISH, as it was the dominant taxon according to pyrosequencing. Quantitative assessment of the relative abundance of *Gammaproteobacteria* (number of cells stained by the specific probe Gam42a in relation to cells stained by the universal bacterial probe EUB338MIX) using cell counts from epifluorescence microscopy images (Supplementary Figure S5A–D), resulted in relative abundances that nicely confirmed the pyrosequencing results (Fig. 6B).

## Discussion

To the best of our knowledge, this study represents the first comprehensive, culture–independent characterization and visualization of the bacterial kitchen sponge microbiome. Previously, Flores and coworkers (2013)^30^ analyzed a single kitchen sponge sample from Boulder in Colorado (USA), and detected 123 OTUs (of which about 20% were singletons) with a phylogenetic diversity metric (PD) of 7.88. Our dataset was based on 14 sponges separated into top and bottom parts, each, and yielded 362 OTUs in total, with an average of 31 OTUs (8–62) and an average PD of 4.28 (1.92–7.46) per sample, not including singleton OTUs. If singleton OTUs were included, the total number of OTUs would increase to 1823 and our dataset would yield an average of 96 OTUs (42–199) and an average PD of 10.06 (5.08–18.50) per sponge sample. Thus, the data from the study of Flores and coworkers (2013)^30^ and our data were quite similar regarding species richness and diversity. However, we preferred to remove singletons, because of the well–known issue of pyrosequencing–generated artifacts^32, 33^. Moreover, by manual screening of the singleton OTUs, no further taxa, including well-known pathogens, were retrieved. Therefore, we decided to exclude singletons to avoid inflation of the diversity indices, although a few members of the rare microbiota might have been excluded from the analysis by doing so.

Flores and co-workers^30^ showed a relative dominance (40.39%) of the family *Moraxellaceae* in their single sponge sample, which is very similar to our value obtained for this family on the basis of 28 samples (36.04%). The relative abundance of other taxa, including *Pseudomonadaceae, Rhizobiaceae*, *Flavobacteriaceae* and *Staphylococcaceae* was also similar; notable differences, however, also appeared for certain taxa, including *Xanthomonadaceae*, *Enterobacteriaceae*, *Sphingomonadaceae*, *Acetobacteraceae* and especially [*Weeksellaceae*] (Supplementary Table S3). These differences might simply result from the different sample numbers, but might also be due to geographical and/or cultural (food, cleaning habits etc.) differences between the underlying countries. While the similarities suggest a kitchen sponge core-microbiome, the more variable fractions might differ from region to region. Clearly, more data from different regions are needed to support this hypothesis, but apparently bacteria affiliated with the *Moraxellaceae* seem “typical” for kitchen sponges. Interestingly, *Moraxellaceae* have been consistently detected on sink surfaces, faucets, refrigerator doors and stoves^30^, i.e. surfaces that might be regularly cleaned with kitchen sponges, suggesting them as source for these surface contaminations. However, *Moraxellaceae* also represent typical human skin bacteria, suggesting also other sources for the contamination of kitchen surfaces. In turn, human skin (hands) might represent a source for the contamination of the sponges with *Moraxellceae* during use. Recently it has been shown that in particular *Moraxellaceae* get significantly enriched on cotton laundry during a domestic washing process^34^.

Notably, *Enterobacteriaceae* were of low relative abundance (1.18%) and were only partly related to genera including pathogenic species, such as *Escherichia* sp. We also screened all excluded singletons and detected only 53 singleton OTUs additionally affiliated with *Enterobacteriaceae*, representing an additional 0.18% of relative abundance of the non-rarified dataset. Also other typical enteropathogenic genera were not detected here, such as *Campylobacter* sp.

The scarce retrieval of bacterial taxa targeted in previous studies of kitchen sponges, such as coliforms, can be explained with the very high abundance of total bacteria in the sponges, which locally exceeded 10^10^ bacterial cells per cm^3^, based on our FISH analyses. CFU counts of total heterotrophic bacteria indicated kitchen sponges as the most densely colonized bacterial reservoir of the BE, with average counts of ~10^7^–10^9^ CFUs per sponge^13, 14, 21, 29^, which is consistent with our observation, assuming that only 1–3% of the environmental bacteria are cultivable on the currently available media^35^. Rossi and coworkers (2012)^19^ reported a retrieval or 2.5 * 10^8^ CFUs/sponge (so, about 10^7^ CFUs per cm^3^) of fecal coliforms, while Josephson *et al*. (1997)^23^ reported 7.8 * 10^8^ per swab area. Averaging these two values (3.9 * 10^8^) and assuming the same amount of *Enterobacteriaceae* in our sponge samples, then a relative abundance of 0.975% should be expected in our samples for the observed bacterial abundance (~4 * 10^10^); our pyrosequencing–detected relative abundance of *Enterobacteriaceae* was about 1.18%, which is very close. Moreover, a bacterial abundance of 4 * 10^10^ was not an average value per sponge, but represented heavily contaminated local sponge sites. Therefore, considering a lower average abundance of bacteria in the sponges, the fraction of *Enterobacteriaceae* would be expected to be even greater than 0.975%, thus getting even closer to the observed value of 1.18%.

We found 5 out of the 10 most abundant OTUs to be closely related to RG2-species from the genera *Acinetobacter*, *Moraxella* and *Chryseobacterium*. Clearly, relatedness based on partial 16S rRNA gene sequences is only a weak indicator for the pathogenic potential of the identified bacteria, and we are not aware of any case, in which an infection with these bacteria was explicitly reported from a domestic environment. Nevertheless, kitchen surfaces are generally regarded as vehicles for transmission of infections^36^ and metagenome reconstruction recently retrieved pathogenicity genes–harboring *Acinetobacter baumanni* genomes from kitchen counters^10^. Therefore, we believe that in view of the high bacterial load of kitchen sponges, the dominance of bacteria closely related to species that clearly can cause infections in humans^37, 38^, warrants attention and underlines the need for appropriate hygiene measures, particularly in BE environments with many immunocompromised persons, such as hospitals, nurseries, schools and houses of home–handled patients. Kitchen sponges are likely to collect, incubate and spread bacteria from and back onto kitchen surfaces, from where they might eventually find their way into the human body, e.g. via the human hands or contaminated food. In addition, direct contact of a sponge with food and/or the human hands might transfer bacteria in and onto the human body, where they might cause infections, depending on their pathogenic potential and the environmental conditions.

Interestingly, in addition to being a RG2-species, *Moraxella osloensis*, is also known for generating malodor in laundry^39^. The abundant occurrence of this bacterium might be responsible for bad smelling kitchen sponges, too. As “special cleaning” measures even increased the relative abundance of *Moraxella*, cleaned sponges might paradoxically smell more often. However, corresponding studies are still missing.

Sanitation by boiling or microwave treatment has been shown to significantly reduce the bacterial load of kitchen sponges^19, 21^ and can therefore be regarded as a reasonable hygiene measure. However, our data showed that regularly sanitized sponges (as indicated by their users) did not contain less bacteria than uncleaned ones. Moreover, “special cleaning” even increased the relative abundance of both the *Moraxella–* and *Chryseobacterium–*affiliated OTUs (Fig. 3B). Presumably, resistant bacteria survive the sanitation process and rapidly re–colonize the released niches until reaching a similar abundance as before the treatment (Fig. 6A). This effect resembles the effect of an antibiotic therapy on the gut microbiota^40, 41^, and might promote the establishment of higher shares of RG2-related species in the kitchen sponges. Although further analyses, including controlled sanitation experiments, are needed to substantiate these findings, our data allow careful speculation that a prolonged application of sanitation measures of kitchen sponges is not advisable.

Analysis of co–occurrence patterns are useful to identify recurrent associations of relevant organisms. We aimed to find out whether non–pathogenic bacteria could act as inhibitors of RG2-related species. However, our data argue against such a protection, instead, associations of RG2-related OTUs were found more often, which suggests a synergy between these species. Whether this has any consequences in terms of clinical relevance remains to be demonstrated.

FISH coupled with confocal microscopy is a robust complementary approach that can be used to validate and support the results of next generation sequencing in microbial ecology studies^42–44^. In this work, FISH–CLSM confirmed the relative abundance of *Gammaproteobacteria* in selected samples. So far, only a single study has directly visualized the bacterial colonization of a kitchen sponge before, however, by electron microscopy^45^, which does not reveal the activity of the inspected cells as the rRNA–based FISH method does. Nevertheless, those images are consistent with our observations, showing dense, biofilm–like clusters of bacterial cells over the sponge surface. Although we calculated the bacterial density of localized, heavy colonized sponge sites (Supplementary Video File S1), a statistically averaged bacterial abundance over whole sponges remains to be assessed. Nevertheless, our work revealed an amazing bacterial colonization of kitchen sponges, and visualized its extent for this common BE–microbial hot spot for the first time. We are convinced that in particular our FISH data (including the Supplementary Video File S1) are very suitable for hygiene education and will help to create even more awareness for kitchen sponges as hygienically relevant microbial incubators.

## Conclusions and Outlook

Our work demonstrated that kitchen sponges harbor a higher bacterial diversity than previously thought, based on cultivation-based studies, and that obligate human pathogens might represent just a minority of their microbiome. Instead, close relatives of RG2-species, not to be expected from previous studies, appeared as dominant taxa. From a long term perspective, sponge sanitation methods appear not sufficient to effectively reduce the bacterial load in kitchen sponges and might even increase the shares of RG2-related bacteria. We therefore rather suggest a regular (and easily affordable) replacement of kitchen sponges, for example, on a weekly basis. In order to verify and complement our study and to better understand the hygienic relevance of the kitchen sponge microbiome, future studies should focus in more detail on i) a differentiation of active and less active (or dead) fractions of the microbiome, e.g. by differential rRNA vs. rRNA gene sequencing, ii) the actual pathogenicity of the kitchen sponge microbiome, e.g. by using a metagenomic or metatranscriptomic approach, iii) the quantitative and qualitative effect of sponge sanitation measures using a controlled experimental setup, iv) the temporal development of the sponge microbiome, v) correlations of the sponge microbiome with the microbiome of the cleaned environments and of the sponge users.

## Material and Methods

### Sample collection of used kitchen sponges

Fourteen used kitchen sponges and associated sponge usage data (Supplementary Table S1) were collected in autumn 2012 from private households in the greater area of Villingen-Schwenningen (Baden-Württemberg, Germany; Supplementary Figure S7). Sponges and usage information were provided voluntarily. No personal data of the human sponge users were recorded, rendering it impossible to assign a specific sponge microbiota to a specific user afterwards. Moreover, the sponge users neither provided any directly health-related personal data, nor were the analyses aimed at the detection of directly health-related bacteria, such as obligate pathogens or MRSA. We therefore believe that the study was performed in an ethically appropriate manner.

For each of the 14 sponges, bottom and top parts (Fig. 1A) were separated with sterile instruments and treated as independent samples (28 in total; Supplementary Table S1), and immediately frozen at −20 °C until DNA extraction or FISH staining. The provided sponge usage data helped to identify possible drivers of bacterial assemblages in the sponges. Our study was designed to test intrinsic (sponge, sponge side, brand/no-brand product) and extrinsic (number of sponge users in the household, frequency of sponge usage, frequency of replacing the sponge, application of special cleaning procedures for the sponge) factors (Supplementary Table S1). In case of the special cleaning procedures, the users were asked to specify whether they regularly apply special measures to clean their sponge. The procedures mentioned were: heating in a microwave and rinsing with hot, soapy water. Despite a relatively low number of total sponges, all investigated factors were sufficiently replicated in an independent manner. For example, in case of the factor “change frequency”, there were 8 sponges monthly-replaced and 6 sponges weekly-replaced (Supplementary Table S1).

### DNA extraction, PCR and 454–pyrosequencing of 16S rRNA gene amplicon libraries

DNA was extracted from all 28 kitchen sponge samples (ca. 75 mg, each) using the FastDNA Spin Kit for Soil according to the manufacturer’s instructions (MP Biomedicals, Eschwege, Germany). A negative extraction without sponge tissue was performed to verify the absence of 16S rRNA genes from the extraction kit.

Amplicon pyrosequencing was performed on a 454 GS FLX Titanium system (Roche, Penzberg, Germany) as described elsewhere^46^. Briefly, barcoded amplicons for multiplexing were prepared using universal primers Ba27f (5′-aga gtt tga tcm tgg ctc ag-3′) and Ba519r (5′-tat tac cgc ggc kgc tg-3′) extended with the respective sequencing adapters, key sequences and multiplex identifiers (MID) as recommended by Roche. Amplicons were purified with the StrataPrep PCR Purification Kit (Agilent Technologies, La Jolla, CA, USA) and pooled in an equimolar ratio of 10^9^ molecules µl^−1^ as quantified by the Quant-iT PicoGreen dsDNA quantification kit (Invitrogen, Paisley, UK). Emulsion PCR, emulsion breaking and sequencing were performed applying the GS FLX Titanium chemistry according to supplier protocols, as previously described^46, 47^.

### Bioinformatic analysis of the sequences

Pyrosequencing data were analyzed with QIIME 1.9^48^. First, the sequences were length-filtered (480–540 nucleotides), then MID, primer, and adapter sequences were removed, and finally quality filtering was applied (quality threshold 25) with final denoising^49^. A maximum of one primer mismatch was allowed. Operational taxonomic units (OTUs) were generated at 97% sequence similarity level (corresponding to the theoretical boundary of species) using the uclust method^50^. After OTU taxonomic identification (via the RDP database, using 0.05 as minimum confidence-level to record an assignment^51^), chimeric OTUs were detected using Chimera Slayer^52^ and removed. Then, singletons as well as plastidic and mitochondrial OTUs were also removed. The dataset was normalized to 357 sequences per sample, and this rarified dataset was used to assess alpha- and beta-diversity, and to identify the OTUs significantly affected by the investigated factors.

Statistical comparison of alpha diversity between samples was performed with Statistica version 12 (StatSoft Inc., Tulsa, OK, USA), using ANOVA and considering top and bottom parts as replicates of the individual sponges. Beta-diversity (phylogenetic distance between samples) was computed by jackknifed-UniFrac weighted pairwise distances^53^, and the effect of the investigated factors was statistically assessed by the Adonis test^54^. Beta-diversity plots (Principal Component Analysis, PCoA) were visualized by Emperor^55^. The list of specific commands used for each QIIME step is available upon request.

### Phylogenetic analysis

In order to identify the most closely related species, the 16S rRNA gene consensus sequences of the ten most abundant OTUs were subjected to a phylogenetic analysis together with the sequences retrieved by manual BLAST^56^ and EzTaxon^57^ alignment. Mega7^58^ was used for the multialignment, using the ClustalW algorithm^59, 60^. Phylogenetic relationships were inferred by using the neighbor joining method with the maximum composite likelihood substitution model^61^. One-thousand bootstrap re–samplings were used to statistically evaluate the tree topology.

### Co–occurrence patterns and network analysis

To identify significant patterns of correlated bacteria in the used kitchen sponges, the OTUs with a relative frequency higher than 0.5% of the total non–rarified sequence dataset (27 OTUs in total) were subjected to a Spearman correlation analysis of the occurrence patterns^44, 62^. Only very strong correlations (*p* < 0.001, R^2^ > 0.6 or < −0.6) were considered and analyzed in a network created with Cytoscape, version 3.3.0^63^.

### Fluorescence *in situ* hybridization – confocal laser scanning microscopy (FISH–CLSM)

Small fragments (fitting inside a 2 ml–vial tube) of frozen sponge samples were fixed with paraformaldehyde for 6 hours at 4 °C, then washed 4 times with ice–cold 1x phosphate buffered saline (PBS) and then stored at −20 °C in 1:1 (vol: vol) 96% Ethanol: 1xPBS, until FISH staining. FISH was performed according to Cardinale *et al*.^64^, using the Cy3–labelled universal bacterial probe EUB338MIX (the equimolar mixture of EUB338^65^, EUB338II and EUB338III^66^) and the FITC–labelled *Gammaproteobacteria*–specific probe Gam42a^67^, used together with its competitor probe (the unlabelled Bet42a probe; Supplementary Table S4). Hybridization was conducted for 2 h at 42 °C and 35% of formamide concentration, followed by washing at 43 °C for 10 min. Negative controls for FISH were obtained by hybridizing separate sponge sub–samples with an equimolar mixture of both FITC– and Cy3–labelled NONEUB probes^68^. FISH–stained samples were mounted with Citifluor AF1 antifade reagent (Linaris Biologische Produkte GmbH, Dossenheim, Germany) and stored at 4 °C in the dark until observation (within one week) with a Leica SP5 confocal laser scanning microscope (Leica Microsystems, Mannheim, Germany) equipped with UV, Argon and He–Ne lasers. FITC and Cy3 were excited with the laser lines 488 and 561 nm, respectively. Sponge tissue was excited additionally with the laser line 405 nm, to induce autofluorescence. Emission was detected in the range 503–535 nm for FITC, 570–613 nm for Cy3 and 412–469 nm for the sponge autofluorescence. Gain and offset were individually adjusted for each field of view in order to optimize the image quality and the signal–to–noise ratio. For each field of view, an appropriate number of optical slices were acquired with a Z-step of 0.5–0.8 μm, and the resulting “confocal stacks” were analyzed with Imaris 8.3.1 (Bitplane, Zurich, Switzerland). Three–dimensional reconstructions were obtained by replacing the original signal with spheres (bacteria) and iso–surfaces (sponge tissue). ImageJ^69^ (available at https://imagej.nih.gov/ij/) and Adobe Photoshop CS6 (Adobe Systems Inc., USA) were used to assemble and label the final figures.

### Comparative quantification of bacterial abundance

For the quantitative assessment of the relative abundance of *Gammaproteobacteria* (the dominant bacterial class according to pyrosequencing), four sponge samples with different metadata were selected: 1 b and 9 b (not regularly sanitized sponges), 3 b and 10 b (regularly sanitized sponges; Supplementary Table S1). After FISH staining, six to eight images per sponge sample were taken with an epifluorescence microscope Zeiss Axioplan 2 (Carl Zeiss Jena GmbH, Jena, Germany), using the Zeiss filter F36-720 HC-mFISH Sp. Green with Brightline HC 515/LP for FITC, and the Zeiss filter set 15 (BP 546, FT 580, LP 590) for Cy3. Cells were counted at the computer and the relative fraction of *Gammaproteobacteria* was calculated as the average percentage of Gam42a–stained cells referred to all EUB338MIX–stained cells. This value was then compared to the expected relative fraction of *Gammaproteobacteria* obtained from the pyrosequencing data. Comparative bacterial abundance between regularly sanitized (“specially cleaned”) and not regularly sanitized sponges was assessed as the average of EUB338MIX–stained cells per image (N = 14).

### Microbiome of unused kitchen sponges

In order to address the microbiome of unused kitchen sponges, 7 new sponges were purchased in local stores in Villingen-Schwenningen and Giessen in early 2017. DNA extraction and 16S rRNA gene PCR (with 5 sponges, i.e. 5 top and 5 bottom samples) and FISH using the universal bacterial probe (with 2 sponges, i.e. 2 top and 2 bottom samples), were performed as described above. In addition, combined top-bottom samples stemming from 2 new sponges were vortexed at maximum speed for one minute in 20 ml of sterile NaCl solution (0.9%). Per sponge, 100 µL of this solution were subsequently spread on a rich solid medium (CASO Agar, Roth, Karlsruhe), which were incubated for 5 d at 28 °C and 37 °C, respectively.

## Electronic supplementary material

Supplementary information

Video file S1
