# Supplementary material for: Microbiome analysis and confocal microscopy of used kitchen sponges reveal massive colonization by Acinetobacter, Moraxella and Chryseobacterium species
Source: Sci Rep. 2017 Jul 19;7:5791. doi: 10.1038/s41598-017-06055-9 (PMC5517580; doi:10.1038/s41598-017-06055-9)
Supplement: Supplementary file 1 — Supplementary information [file 41598_2017_6055_MOESM1_ESM.pdf]

Supplementary material for

Microbiome analysis and confocal microscopy of used kitchen sponges reveal massive colonization by *Acinetobacter*, *Moraxella* and *Chryseobacterium* species

Massimiliano Cardinale, Dominik Kaiser, Tillmann Lueders, Sylvia Schnell &

Markus Egert

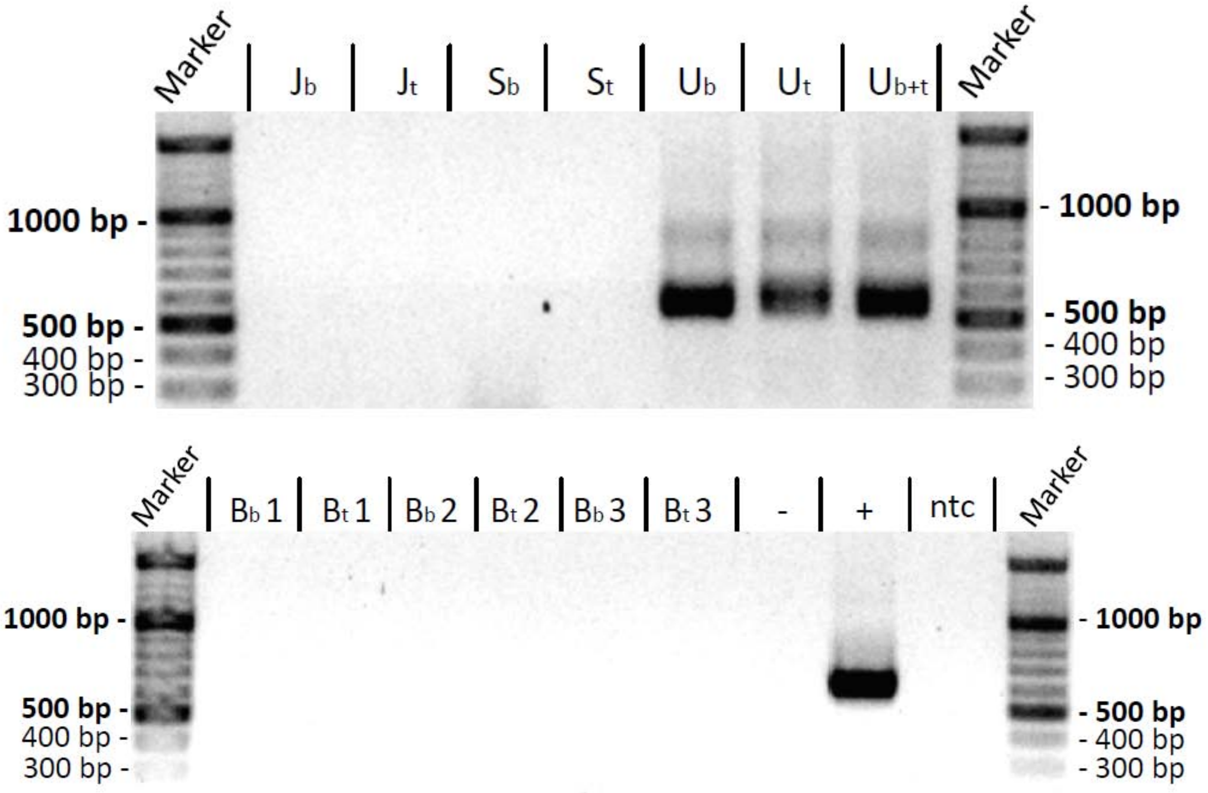

Figure S1. Results of a PCR assay targeting the16S rRNA gene in DNA extracts from five newly bought kitchen sponges (J, S, B; t = top, b = bottom). All new sponges did not yield PCR signals, while a used sponge sample (U) clearly did. Also a DNA extract from a negative extraction (-) yielded no PCR product. *E. coli* DNA was used a positive control. Ntc = no template control.

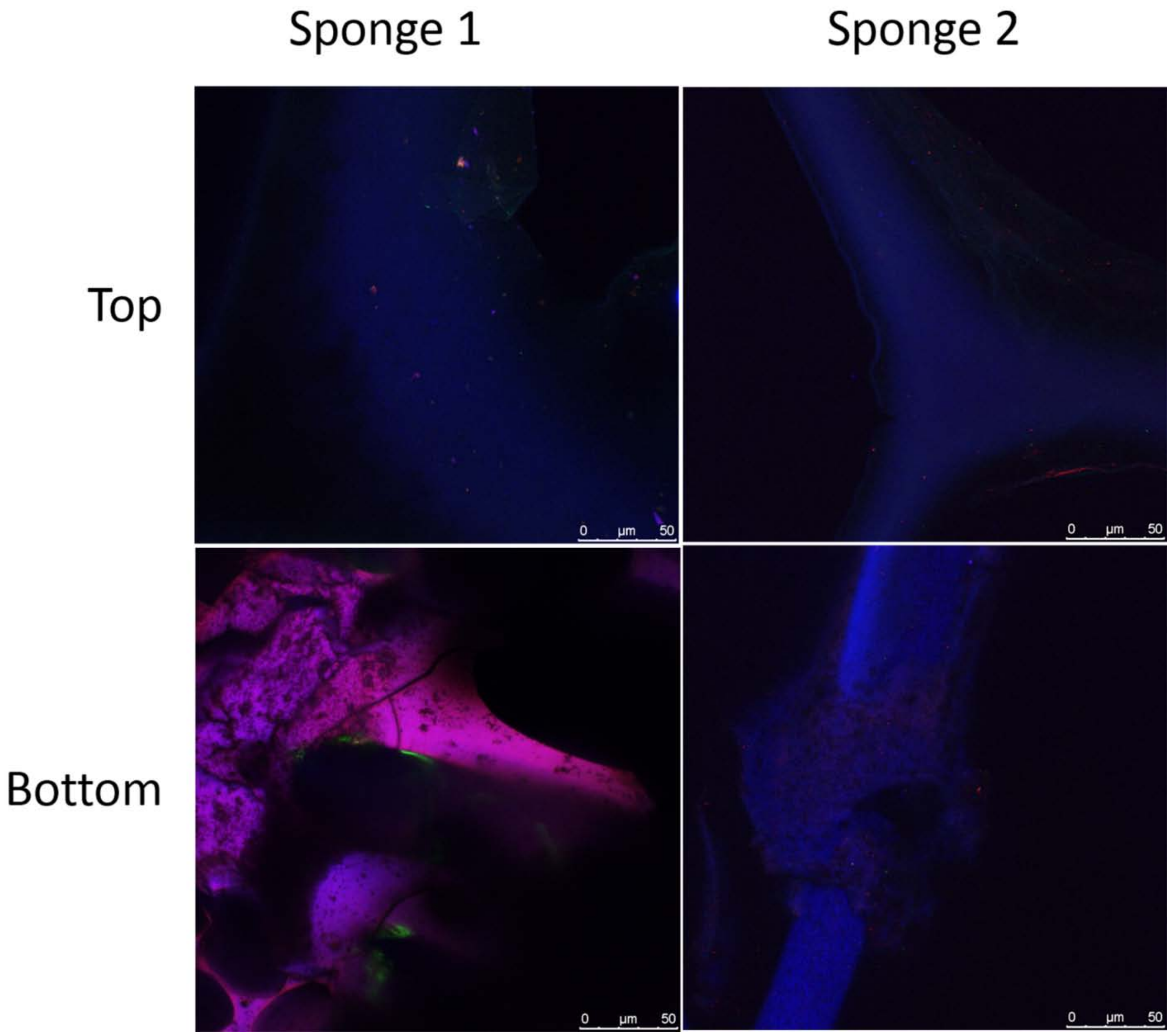

Figure S2 FISH–CLSM analysis of new kitchen sponges using the universal bacterial probe EUB338MIX labeled with Cy3 (expected signal: red) and the Gammaproteobacteria–specific probe Gam42a labelled with Cy5 (expected signal: green); no bacterial signals were detected. Blue= sponge autofluorescence.

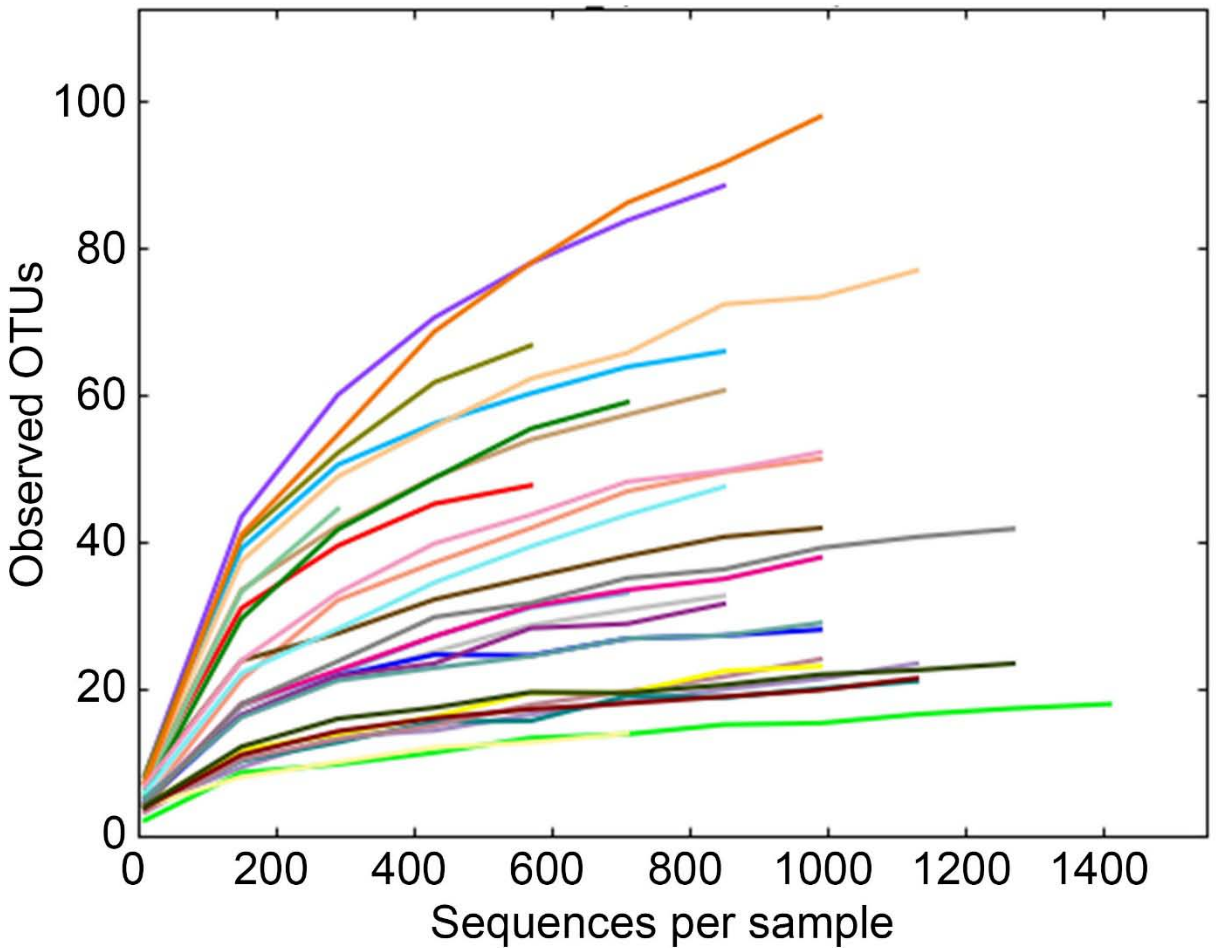

Figure S3 Rarefaction curves of the 28 sponge samples analyzed by pyrosequencing of the 16S rRNA amplicon libraries

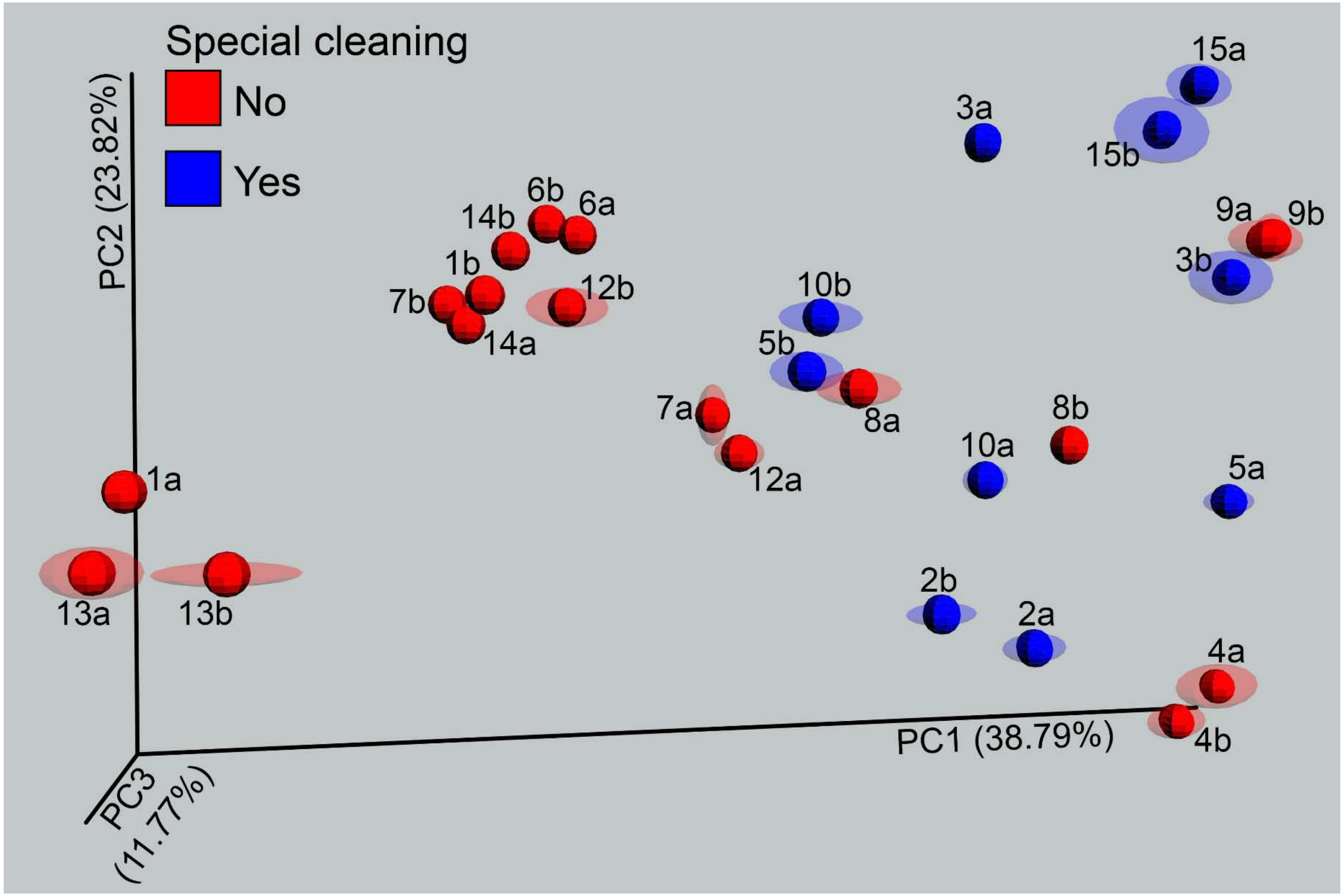

Figure S4 Beta–diversity plot of jacknifed weighted–Unifrac distances between sponge samples, colored by "special cleaning", the only significant factor found (ADONIS, $p = 0.022$), and labeled by sample name (numbers indicate the individual sponges; letters indicate the sponge side, a= bottom, b= top)

.

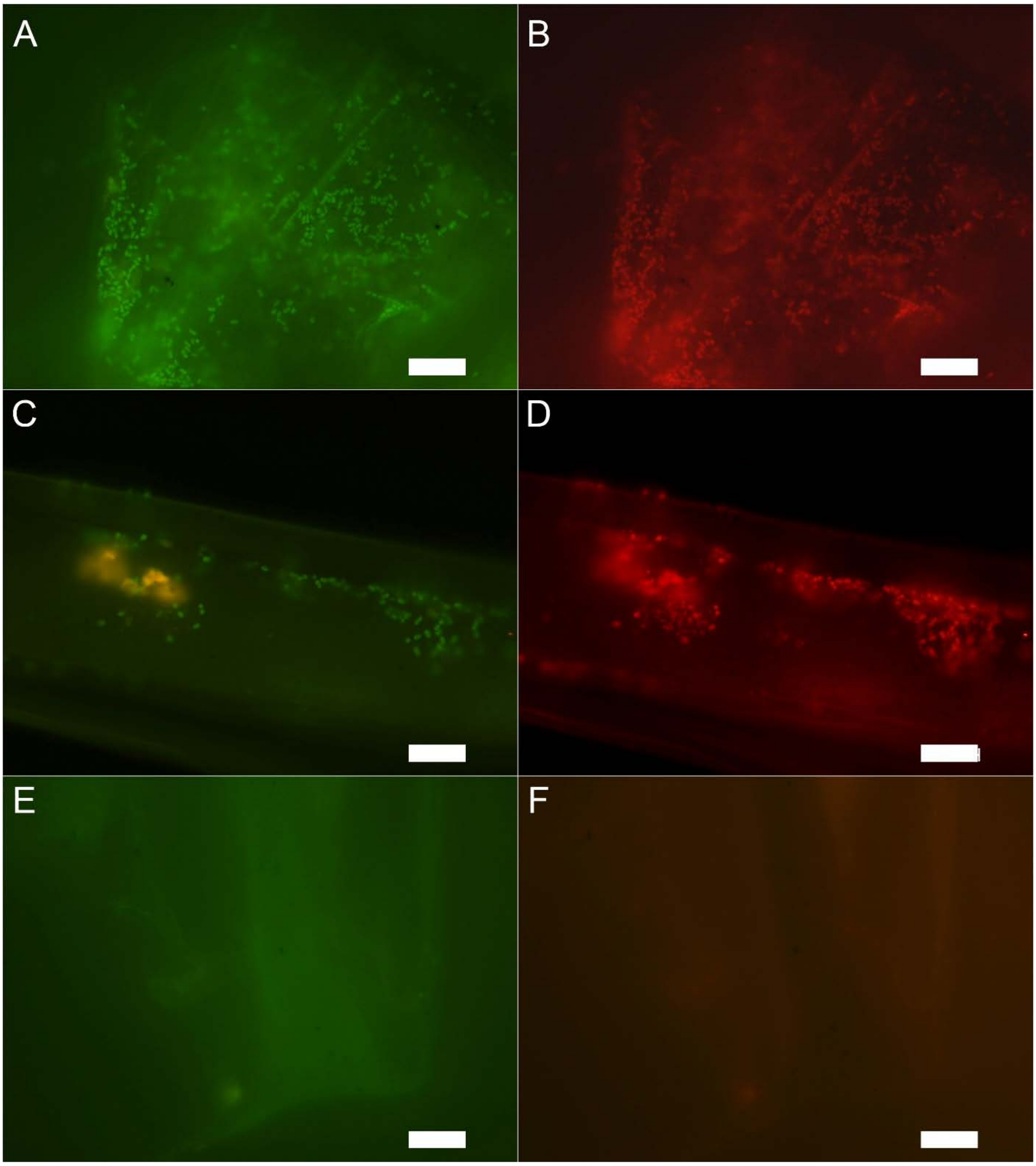

Figure S5 Examples of epifluorescent microscopy images used to assess the relative abundance of *Gammaproteobacteria* in selected FISH–stained sponge samples. A and C) Signal of FITC–labelled FISH probes Gam42a. B and D) Signal of the Cy3–labelled FISH probes EUB338MIX. E and F) FISH negative control, stained with the non–sense probe NONEUB labelled with FITC and Cy3, respectively. Scale bars: 15 µm.

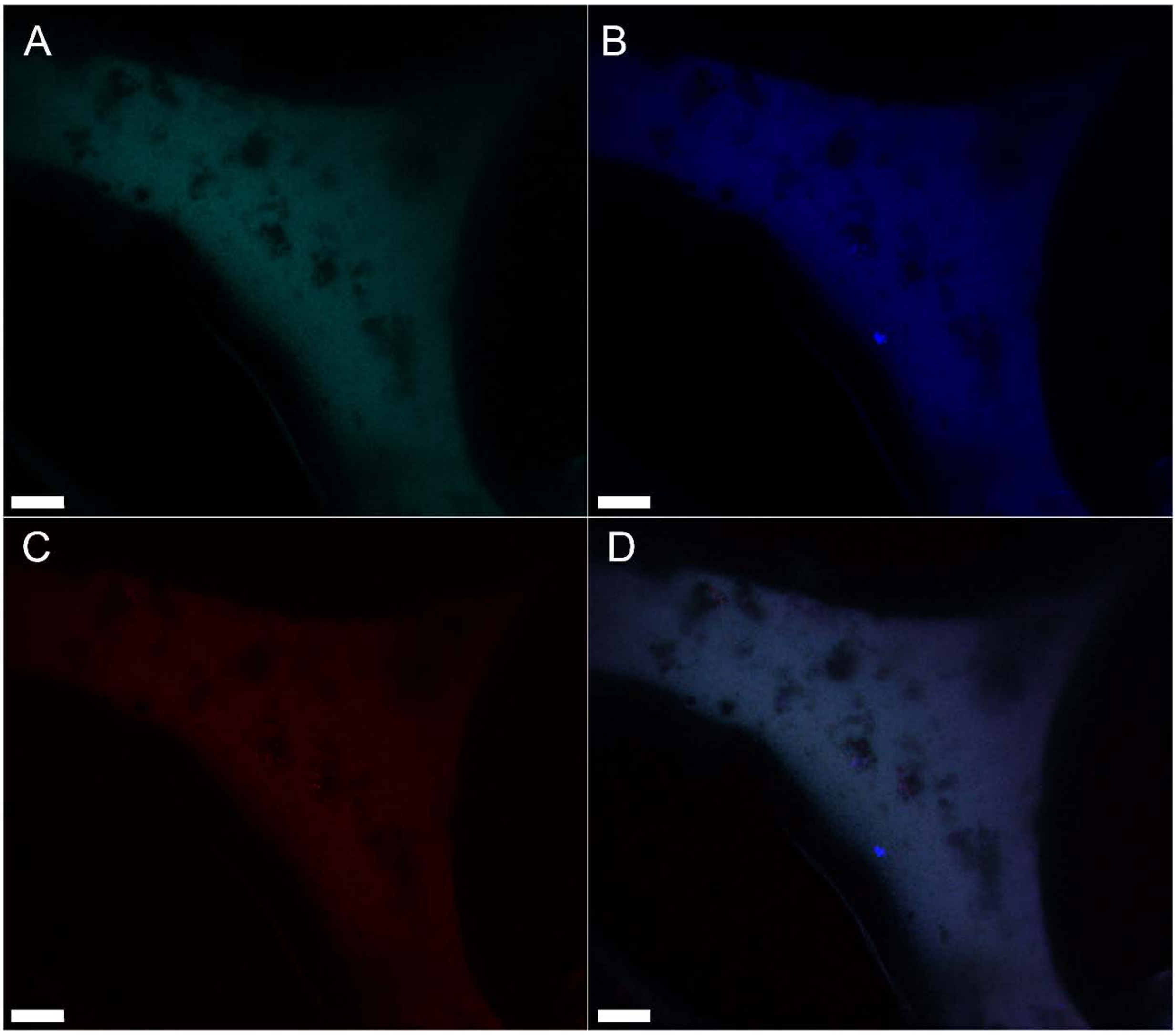

Figure S6 Maximum projection of a confocal laser–scanning microscopy image series, showing the FISH negative control of the kitchen sponge sample 6b. A) Sponge autofluorescence. B) Signal of FITC–labelled NONEUB probe. C) Signal of Cy3–labelled NONEUB probe. D) Overlap of A–C. Scale bars: 20 µm.

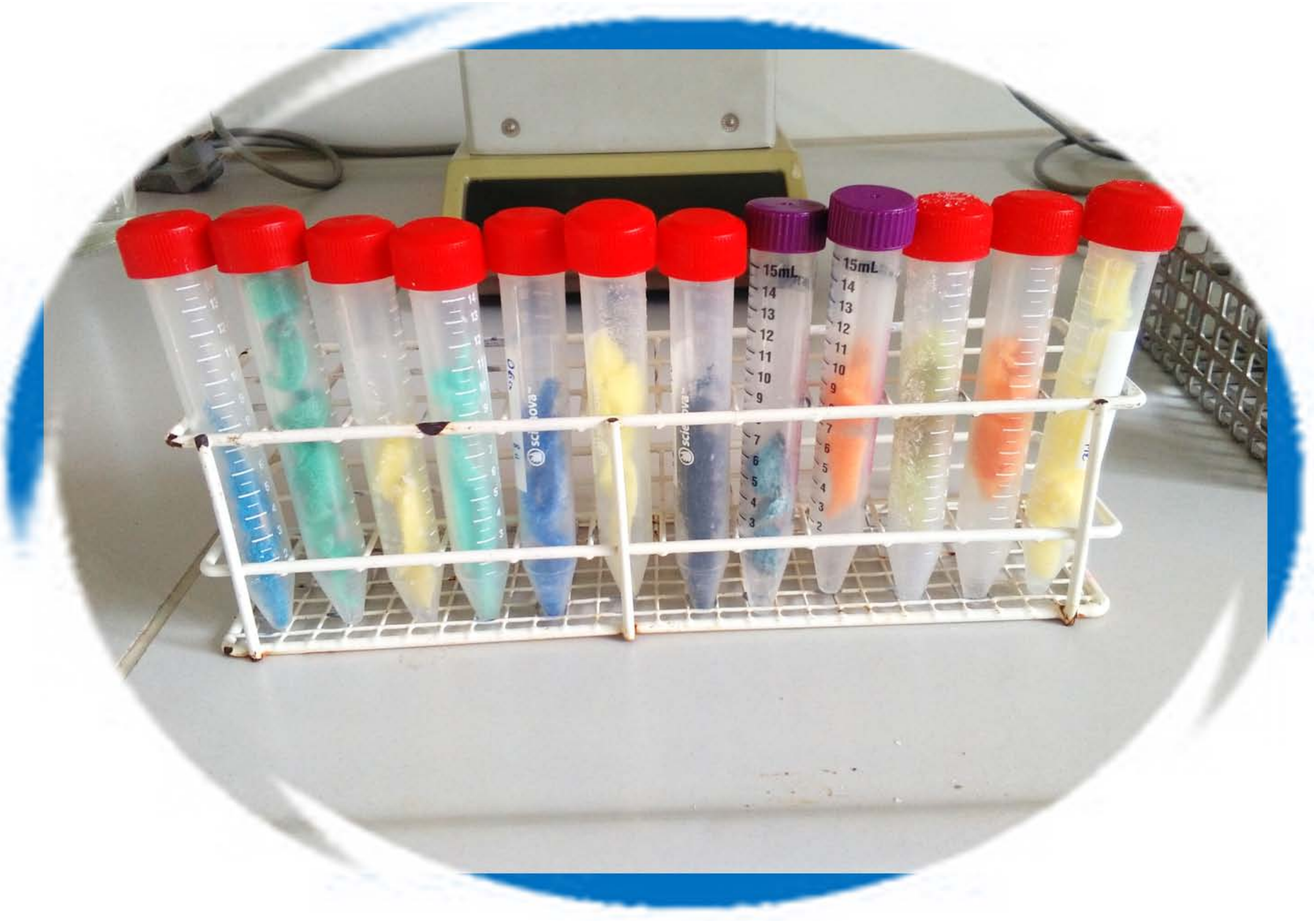

Figure S7 Examples of sponge samples used in this work

Table S1 Sponge samples analyzed in this work and their respective metadata

Sample	Intrinsic factors			Extrinsic factors			
	Sponge	Sponge side	Brand product	Change frequency	Use frequency	Special cleaning	Persons per household
1a	1	Bottom	No	Weekly	Not daily	No	1-3
2a	2	Bottom	No	Monthly	Not daily	Yes	1-3
3a	3	Bottom	Yes	Monthly	Daily	Yes	1-3
4a	4	Bottom	No	Weekly	Daily	No	5-20
5a	5	Bottom	No	Weekly	Daily	Yes	1-3
6a	6	Bottom	Yes	Monthly	Daily	No	1-3
7a	7	Bottom	No	Weekly	Not daily	No	1-3
8a	8	Bottom	No	Weekly	Not daily	No	1-3
9a	9	Bottom	Yes	Monthly	Daily	No	5-20
10a	10	Bottom	No	Monthly	Daily	Yes	1-3
12a	12	Bottom	No	Monthly	Daily	No	5-20
13a	13	Bottom	Yes	Monthly	Daily	No	1-3
14a	14	Bottom	No	Weekly	Not daily	No	1-3
15a	15	Bottom	No	Monthly	Daily	Yes	1-3
1b	1	Top	No	Weekly	Not daily	No	1-3
2b	2	Top	No	Monthly	Not daily	Yes	1-3
3b	3	Top	Yes	Monthly	Daily	Yes	1-3
4b	4	Top	No	Weekly	Daily	No	5-20
5b	5	Top	No	Weekly	Daily	Yes	1-3
6b	6	Top	Yes	Monthly	Daily	No	1-3
7b	7	Top	No	Weekly	Not daily	No	1-3
8b	8	Top	No	Weekly	Not daily	No	1-3
9b	9	Top	Yes	Monthly	Daily	No	5-20
10b	10	Top	No	Monthly	Daily	Yes	1-3
12b	12	Top	No	Monthly	Daily	No	5-20
13b	13	Top	Yes	Monthly	Daily	No	1-3
14b	14	Top	No	Weekly	Not daily	No	1-3
15b	15	Top	No	Monthly	Daily	Yes	1-3

Table S2 Alpha–diversity metrics of the used kitchen sponge samples analyzed in this work, as calculated from the rarefied pyrosequencing dataset at an even depth of 357 sequences per sample. Sample number indicate the individual sponge, while letters indicate the top (a) or bottom (b) site. The last row shows the significance of the factor "sponge" for each index (in red the significant values at $p < 0.05$).

Sample	Good's coverage	Shannon	Equitability	Dominance	Simpson	Simpson reciprocal[#]	Simpson evenness	PD whole tree	Chao1	Number of OTUs
1a	0.972	4.167	0.783	0.086	0.914	11.600	0.290	5.366	44.500	40
2a	0.927	4.385	0.739	0.112	0.888	8.927	0.146	7.253	88.083	61
3a	0.975	2.807	0.604	0.233	0.767	4.283	0.171	3.736	30.143	25
4a	0.944	3.402	0.669	0.146	0.854	6.830	0.201	5.241	97.333	34
5a	0.980	2.889	0.648	0.217	0.783	4.617	0.210	3.060	29.000	22
6a	0.975	2.499	0.532	0.365	0.635	2.742	0.105	4.301	32.000	26
7a	0.950	2.978	0.576	0.224	0.776	4.459	0.124	5.993	66.600	36
8a	0.958	4.565	0.801	0.067	0.933	14.996	0.288	7.457	59.000	52
9a	0.983	2.126	0.558	0.329	0.671	3.037	0.217	2.728	29.000	14
10a	0.969	2.947	0.627	0.197	0.803	5.077	0.195	3.485	37.000	26
12a	0.955	5.044	0.872	0.040	0.960	24.733	0.450	5.132	67.000	55
13a	0.944	4.772	0.815	0.060	0.940	16.563	0.286	7.465	77.000	58
14a	0.983	1.617	0.425	0.531	0.469	1.885	0.135	2.193	19.000	14
15a	0.994	1.879	0.508	0.448	0.552	2.233	0.172	2.657	13.250	13
1b	0.986	2.610	0.604	0.265	0.735	3.778	0.189	3.041	22.500	20
2b	0.947	3.865	0.712	0.133	0.867	7.509	0.175	5.632	71.500	43
3b	0.969	2.286	0.538	0.286	0.714	3.495	0.184	2.961	46.500	19
4b	0.964	3.548	0.692	0.147	0.853	6.790	0.194	5.661	46.143	35
5b	0.980	3.746	0.788	0.103	0.897	9.743	0.361	3.432	37.500	27
6b	0.986	0.927	0.251	0.749	0.251	1.334	0.103	2.513	15.000	13
7b	0.969	2.127	0.464	0.430	0.570	2.327	0.097	3.112	33.167	24
8b	0.952	4.092	0.737	0.110	0.890	9.072	0.193	6.561	64.000	47
9b	0.997	1.638	0.546	0.412	0.588	2.425	0.303	1.918	8.000	8
10b	0.972	2.618	0.551	0.325	0.675	3.074	0.114	3.857	38.250	27

12b	0.955	4.172	0.764	0.098	0.902	10.185	0.231	4.406	57.333	44
13b	0.927	5.067	0.851	0.042	0.958	23.791	0.384	5.867	102.625	62
14b	0.983	2.387	0.627	0.238	0.762	4.201	0.300	1.924	29.000	14
15b	0.989	2.250	0.550	0.378	0.622	2.644	0.156	2.892	18.500	17
ANOVA (between sponges), *p* value	< 0.001	< 0.001	0.005	0.012	0.012	0.045##	0.08	0.001	0.003	< 0.001

Not normally distributed variable

Kruskall–Wallis test

Table S3 Comparison of the relative abundance of major taxa (family level) between our work and the sponge sample analyzed by Flores *et al.*, 2013. Mean (± Std error for this work's samples) is indicated.

Family	Relative abundance (% of microbiome)	
	This work	Flores *et al*., 2013*
Moraxellaceae	36.04 ± 4.86	40.39
Pseudomonadaceae	9.58 ± 3.55	7.48
Rhizobiaceae	4.01 ± 0.78	3.22
Flavobacteriaceae	1.31 ± 0.76	1.98
Staphylococcaceae	0.037 ± 0.022	0.02
[*Weeksellaceae*]	21.90 ± 3.70	n.d.**
Sphingomonadaceae	3.12 ± 1.02	0.42
Xanthomonadaceae	3.21 ± 1.25	16.16
Enterobacteriaceae	1.18 ± 0.24	16.86
Acetobacteraceae	0.18 ± 0.09	4.40

*Flores, G. E., Bates, S. T., Caporaso, J. G., Lauber, C. L., Leff, J. W., Knight, R. & Fierer, N. Diversity, distribution and sources of bacteria in residential kitchens. *Env Microbiol* **15**, 588–596 (2013)
**n.d. = not detected

Table S4 Properties of the FISH probes used in this work

Probe name	Sequence (5'–3')	Fluorochrome	Target	% Formamide (at 42°C)
EUB338[*]	GCTGCCTCCCGTAGGAGT	Cy3	Most bacteria	35
EUB338II[*]	GCAGCCACCCGTAGGTGT	Cy3	*Planctomycetales*	35
EUB338III[*]	GCTGCCACCCGTAGGTGT	Cy3	*Verrucomicrobiales*	35
Gam42a[**]	GCCTTCCCACATCGTTT	FITC	*Gammaproteobacteria*	35
Gam42a–competitor (unlabelled Bet42a probe)[**]	GCCTTCCCACTTCGTTT	/	*Betaproteobacteria*	35
NONEUB[***]	ACTCCTACGGGAGGCAGC	Cy3	/	35
NONEUB[***]	ACTCCTACGGGAGGCAGC	FITC	/	35

*, ** and *** used mixed in equimolar concentration

Log file S1 Results of length/quality filtering of the pyrosequencing reads, obtained with QIIME 1.9.

Number raw input seqs	223741

Length outside bounds of 480 and 540	136675
Num ambiguous bases exceeds limit of 6	197
Missing Qual Score	0
Mean qual score below minimum of 25	5245
Max homopolymer run exceeds limit of 6	363
Num mismatches in primer exceeds limit of 1:	46882

Sequence length details for all sequences passing quality filters:

Raw len min/max/avg	480.0/540.0/516.7
Wrote len min/max/avg	450.0/510.0/486.7

Barcodes corrected/not 281/1198

Uncorrected barcodes will not be written to the output fasta file.

Corrected barcodes will be written with the appropriate barcode category.

Corrected but unassigned sequences will not be written unless --retain_unassigned_reads is enabled.

Total valid barcodes that are not in mapping file 0

Sequences associated with valid barcodes that are not in the mapping file will not be written.

Barcodes in mapping file

Num Samples 28

Sample ct min/max/mean: 386 / 1520 / 1185.04

Sample	Sequence Count	Barcode
15b	1520	AGTACGCTAT
6b	1453	TAGAGACGAG
4b	1417	TACGAGTATG
2a	1402	ACGCTCGACA
13b	1385	AGACTATACT
15a	1383	TCTACGTAGC
9a	1379	TAGTATCAGC
6a	1375	ATATCGCGAG
5b	1351	TACTCTCGTG
1b	1347	CGTGTCTCTA
12b	1275	ACTGTACAGT
12a	1256	ATACGACGTA
14a	1249	CGTCTAGTAC
3a	1230	AGACGCACTC
8a	1222	ACGACTACAG
5a	1216	ATCAGACACG
14b	1206	AGCGTCGTCT
10a	1108	TCTCTATGCG
7a	1098	TGTACTACTC
3b	1098	CGTAGACTAG
4a	1095	AGCACTGTAG
10b	1082	ACTACTATGT

2b	1028	CTCGCGTGTC
1a	1008	ACGAGTGCGT
13a	944	TCACGTACTA
9b	903	ACGCGAGTAT
7b	765	TCGTCGCTCG
8b	386	ACATACGCGT
Total number seqs written		33181

Video file S1: Video animation of the sponge sample 9B. The sponge sample was stained by FISH, using the Cy3–labelled EUB338MIX probe, and observed by confocal microscopy; this confocal series is composed of 52 optical slices and was acquired using a Z–step of 0.63µm. The three–dimensional model was created with the software Imaris 8.3 (Bitplane, Zurich, Switzerland), by converting the sponge autofluorescence signals into iso–surfaces and the bacterial FISH–conferred signals into spheres, respectively.

Partial 16S rRNA gene sequences of the OTUs displayed in Fig. 2 of the main text.

Not aligned

>OTU586 (Sphingobium)
AACGAACGCTGGCGGCATGCCTAATACATGCAAGTCGAACGAGATCTTCGGATCTA
GTGGCGCACGGGTGCGTAACGCGTGGGAATCTGCCCTTGGGTTCGGAATAACTTCT
GGAAACGGAAGCTAATACCGGATGATGACGTAAGTCCAAAGATTTATCGCCCAAG
GATGAGCCCGCGTAGGATTAGCTAGTTGGTGGGGTAAAGGCTCACCAAGGCGACG
ATCCTTAGCTGGTCTGAGAGGATGATCAGCCACACTGGGACTGAGACACGGCCCA
GACTCCTACGGGAGGCAGCAGTAGGGAATATTGGACAATGGGCGAAAGCCTGATC
CAGCAATGCCGCGTGAGTGATGAAGGCCTTAGGGTTGTAAAGCTCTTTTACCCGGG
ATGATAATGACAGTACCGGAGAATAAGCTCCGGCTAACTCCGTGCCAGCAGCCGC
GGTAATATAGAGACACG
>OTU2089 (Agrobacterium)
AACGAACGCTGGCGGCAGGCTTAACACATGCAAGTCGAACGCCCCGCAAGGGGAG
TGGCAGACGGGTGAGTAACGCGTGGGAACATACCCTTTCCTGCGGAATAGCTCCGG
GAAACTGGAATTAATACCGCATACGCCCTACGGGGGAAAGATTTATCGGGGAAGG
ATTGGCCCGCGTTGGATTAGCTAGTTGGTGGGGTAAAGGCCTACCAAGGCGACGAT
CCATAGCTGGTCTGAGAGGATGATCAGCCACATTGGGACTGAGACACGGCCCAAA
CTCCTACGGGAGGCAGCAGTGGGGAATATTGGACAATGGGCGCAAGCCTGATCCA
GCCATGCCGCGTGAGTGATGAAGGCCTTAGGGTTGTAAAGCTCTTTCACCGATGAA
GATAATGACGGTAGTCGGAGAAGAAGCCCCGGCTAACTTCGTGCCAGCCGCCGCG
GTAATACTGTAGTCGT
>OTU1492 (Acinetobacter)
ATTGAACGCTGGCGGCAGGCTTAACACATGCAAGTCGAGCGGAGAGAGGTAGCTT
GCTACTGATCTTAGCGGCGGACGGGTGAGTAATGCTTAGGAATCTGCCTATTAGTG
GGGGACAACATTTCGAAAGGAATGCTAATACCGCATACGTCCTACGGGAGAAAGC
AGGGGATCTTCGGACCTTGCGCTAATAGATGAGCCTAAGTCGGATTAGCTAGTTGG
TGGGTAAAGGCCTACCAAGGCGACGATCTGTAGCGGGTCTGAGAGGATGATCCGC
CACACTGGGACTGAGACACGGCCCAGACTCCTACGGGAGGCAGCAGTGGGGAATA
TTGGACAATGGGCGCAAGCCTGATCCAGCCATGCCGCGTGTGTGAAGAAGGCCTTA
TGGTTGTAAAGCACTTTAAGCGAGGAGGAGGCTACTTTAGTAATACCTAGAGATAG
TGGACGTTACTCGCAGAATAAGCACCGGCTAACTCCGTAGCCG
>OTU47 (Chryseobacterium)
GATGGACGCTAGCGGGAGGCCTAACACATGCAAGCCGAGCGGTAGAGTTTCTTCG
GAAACTTGAGAGCGGCGCACGGGTGCGTAACACGTGTGCAACCTACCTTTATCTGG
GGGATAGCCTTTCGAAAGGAAGATTAATACCCCATAATATAATGATTGGCATCAAT
TATTATTGAAAACTCCGGTGGATAGAGATGGGCACGCGCAAGATTAGATAGTTGGT
GAGGTAACGGCTCACCAAGTCAATGATCTTTAGGGGTCCTGAGAGGGAGATCCCCC
ACACTGGTACTGAGACACGGACCAGACTCCTACGGGAGGCAGCAGTGAGGAATAT
TGGACAATGGGTGAGAGCCTGATCCAGCCATCCCGCGTGAAGGATGACGGTCCTAT
GGATTGTAAACTTCTTTTGTACAGGGATAAACCTACTCTCGTGAGGGTAGCTGAAG
GTACTGTACGAATAAGCACCGGCTAACTCCGTGCCAGCAGCCGCGGTAATACG
>OTU2007 (Pseudomonas)
ATTGAACGCTGGCGGCAGGCCTAACACATGCAAGTCGAGCGGATGATGGGAGCTT
GCTCCTGGATTCAGCGGCGGACGGGTGAGTAATGCCTAGGAATCTGCCTGGTAGTG
GGGGACAACGTTTCGAAAGGAACGCTAATACCGCATACGTCCTACGGGAGAAAGC
AGGGGACCTTCGGGCCTTGCGCTATCAGATGAGCCTAGGTCGGATTAGCTTGTTGG
TGAGGTAATGGCTCACCAAGGCGACGATCCGTAACTGGTCTGAGAGGATGATCAG
TCACACTGGAACTGAGACACGGTCCAGACTCCTACGGGAGGCAGCAGTGGGGAAT

ATTGGACAATGGGCGAAAGCCTGATCCAGCCATGCCGCGTGTGTGAAGAAGGTCTT
CGGATTGTAAAGCACTTTAAGTTGGGAGGAAGGGTTGTAGATTAATACTCTGCAAT
TTTGACGTTTACCGACAGAATAAGCACCGGCTAACTCTGTGCC
>OTU99 (Moraxella)
ATTGAACGCTGGCGGCAGGCTTAACACATGCAAGTCGAACGATGACTCTCTAGCTT
GCTAGAGATGATTAGTGGCGGACGGGTGAGTAACATTTAGGAATCTACCTAGTAGT
GGGGGATAGCTCGGGGAAACTCGAATTAATACCGCATACGACCTACGGGTGAAAG
GGGGCGCAAGCTCTTGCTATTAGATGAGCCTAAATCAGATTAGCTAGTTGGTGGGG
TAAAGGCCCACCAAGGCGACGATCTGTAACTGGTCTGAGAGGATGATCAGTCACA
CCGGAACTGAGACACGGTCCGGACTCCTACGGGAGGCAGCAGTGGGGAATATTGG
ACAATGGGGGCAACCCTGATCCAGCCATGCCGCGTGTGTGAAGAAGGCCTTTTGGT
TGTAAAGCACTTTAAGCAGGGAGGAGAGGCTAATGGTTAATACCCATTAGATTAG
ACGTTACCTGCAGAATAAGCACCGGCTAACTCTGTGCCAGCAGCCGACGG
>OTU3112 (Chryseobacterium)
GATGAACGCTAGCGGGAGGCCTAACACATGCAAGCCGAGCGGTATTTGTTCTTCGG
AACAGAGAGAGCGGCGCACGGGTGCGGAACACGTGTGCAACCTGCCTTTATCTGG
GGGATAGCCTTTCGAAAGGAAGATTAATACCCCATAATATATTGAGTGGCATCATT
TGATATTGAAAACTCCGGTGGATAGAGATGGGCACGCGCAAGATTAGATAGTTGG
TGAGGTAACGGCTCACCAAGTCAATGATCTTTAGGGGGCCTGAGAGGGTGATCCCC
CACACTGGTACTGAGACACGGACCAGACTCCTACGGGAGGCAGCAGTGAGGAATA
TTGGACAATGGGTTAGCGCCTGATCCAGCCATCCCGCGTGAAGGACGACGGCCCTA
TGGGTTGTAAACTTCTTTTGTATAGGGATAAACCTACTCTCGTGAGAGTAGCTGAA
GGTACTATACGAATAAGCACCGGCTAACTCCGTGCCAGCAGCCGACGG
>OTU1844 (Brevundimonas)
AGCGAACGCTGGCGGCAGGCCTAACACATGCAAGTCGAACGGACCCTTCGGGGTT
AGTGGCGGACGGGTGAGTAACACGTGGGAACGTGCCTTTAGGTTCGGAATAGCTC
CTGGAAACGGGTGGTAATGCCGAATGTGCCCTTCGGGGGAAAGATTTATCGCCTTT
AGAGCGGCCCGCGTCTGATTAGCTAGTTGGTGAGGTAACGGCTCACCAAGGCGAC
GATCAGTAGCTGGTCTGAGAGGATGACCAGCCACACTGGGACTGAGACACGGCCC
AGACTCCTACGGGAGGCAGCAGTGGGGAATCTTGCGCAATGGGCGAAAGCCTGAC
GCAGCCATGCCGCGTGAATGATGAAGGTCTTAGGATTGTAAAATTCTTTCACCGGG
GACGATAATGACGGTACCCGGAGAAGAAGCCCCGGCTAACTTCGTGCCAGCAGCC
GCGGTAATACACGAGAGTA
>OTU893 (Acinetobacter)
ATTGAACGCTGGCGGCAGGCTTAACACATGCAAGTCGAGCGGGGAAAGGTAGCTT
GCTACCTGACCTAGCGGCGGACGGGTGAGTAATGCTTAGGAATCTGCCTATTAGTG
GGGGACAACATTCCGAAAGGAATGCTAATACCGCATACGCCCTACGGGGGAAAGC
AGGGGATCTTCGGACCTTGCGCTAATAGATGAGCCTAAGTCAGATTAGCTAGTTGG
TGGGGTAAAGGCCTACCAAGGCGACGATCTGTAGCGGGTCTGAGAGGATGATCCG
CCACACTGGGACTGAGACACGGCCCAGACTCCTACGGGAGGCAGCAGTGGGGAAT
ATTGGACAATGGGCGAAAGCCTGATCCAGCCATGCCGCGTGTGTGAAGAAGGCCT
TTTGGTTGTAAAGCACTTTAAGCGAGGAGGAGGCTACTTAGATTAATACTCTAGGA
TAGTGGACGTTACTCGCAGAATAAGCACCGGCTAACTCTGTGCCAGCCGCCG
>OTU2385 (Acinetobacter)
ATTGAACGCTGGCGGCAGGCTTAACACATGCAAGTCGAGCGGAGAGAAGTAGCTT
GCTACTGATCTTAGCGGCGGACGGGTGAGTAATACTTAGGAATCTGGCTATTAGTG
GGGGACAACATTTCGAAAGGGATGCTAATACCGCATACGTCCTACGGGAGAAAGC
AGGGGATCTTCGGACCTTGCGCTAATAGATGAGCCTAAGTCGGATTAGCTAGTTGG
TGGGGTAAAGGCCTACCAAGGCGACGATCTGTAGCGGGTCTGAGAGGATGATCCG
CCACACTGGGACTGAGACACGGCCCAGACTCCTACGGGAGGCAGCAGTGGGGAAT
ATTGGACAATGGGGGGAACCCTGGATCCAGCCATGCCGCGTGTGTGAAGAAGGCC

TTATGGTTGTAAAGCACTTTAAGCGAGGAGGAGGGTACTGGTATTAATACTACCAG
GTACTGGACGTTACTCGCAGAATAAGCACCGGCTAACTCTGTGCCAGCCGCGAC
>Moraxella osloensis DSM 6998 (NR113392)
GAGTTTGATCCTGGCTCAGATTGAACGCTGGCGGCAGGCTTAACACATGCAAGTCG
AACGATGACTCTCTAGCTTGCTAGAGATGATTAGTGGCGGACGGGTGAGTAACATT
TAGGAATCTACCTAGTAGTGGGGGATAGCTCGGGGAAACTCGAATTAATACCGCAT
ACGACCTACGGGTGAAAGGGGGCGCAAGCTCTTGCTATTAGATGAGCCTAAATCA
GATTAGCTAGTTGGTGGGGTAAAGGCCCACCAAGGCGACGATCTGTAACTGGTCTG
AGAGGATGATCAGTCACACCGGAACTGAGACACGGTCCGGACTCCTACGGGAGGC
AGCAGTGGGGAATATTGGACAATGGGGGCAACCCTGATCCAGCCATGCCGCGTGT
GTGAAGAAGGCCTTTTGGTTGTAAAGCACTTTAAGCAGGGAGGAGAGGCTAATGG
TTAATACCCATTAGATTAGACGTTACCTGCAGAATAAGCACCGGCTAACTCTGTGC
CAGCAGCCGCGGTAATACAGAGGGTGCGAGCGTTAATCGGAATTACTGGGCGTAA
AGCGAGTGTAGGTGGCTCATTAAGTCACATGTGAAATCCCCGGGCTTAACCTGGGA
ACTGCATGTGATACTGGTGGTGCTAGAATATGTGAGAGGGAAGTAGAATTCCAGGT
GTAGCGGTGAAATGCGTAGAGATCTGGAGGAATACCGATGGCGAAGGCAGCTTCC
TGGCATAATATTGACACTGAGATTCGAAAGCGTGGGTAGCAAACAGGATTAGATA
CCCTGGTAGTCCACGCCGTAAACGATGTCTACTAGCCGTTGGGGTCCTTGAGACTT
TAGTGGCGCAGTTAACGCGATAAGTAGACCGCCTGGGGAGTACGGCCGCAAGGTT
AAAACTCAAATGAATTGACGGGGGCCCGCACAAGCGGTGGAGCATGTGGTTTAAT
TCGATGCAACGCGAAGAACCTTACCTGGTCTTGACATAGTGAGAATCTYTCAGAGA
TGAGAGAGTGCCTTCGGGAACTCACATACAGGTGCTGCATGGCTGTCGTCAGCTCG
TGTCGTGAGATGTTGGGTTAAGTCCCGCAACGAGCGCAACCCTTTTCCTTATTTGCC
AGCGGGTTAAGCCGGGAACTTTAAGGATACTGCCAGTGACAAACTGGAGGAAGGC
GGGGACGACGTCAAGTCATCATGGCCCTTACGACCAGGGCTACACACGTGCTACA
ATGGTAGGTACAGAGGGTTGCTACACAGCGATGTGATGCTAATCTCAAAAAGCCTA
TCGTAGTCCGGATTGGAGTCTGCAACTCGACTCCATGAAGTCGGAATCGCTAGTAA
TCGCGGATCAGAATGCCGCGGTGAATACGTTCCCGGGCCTTGTACACACCGCCCGT
CACACCATGGGAGTCTATTGCACCAGAAGTAGGTAGCCTAACGAAAGAGGGCGCT
TACCACGGTGTGGTCGATGACTGGGGTGAAGTCGTAACAAGGTAGCC
>Moraxella osloensis A1920 (NR104936)
TAGAGTTTGATCATGGCTCAGATTGAACGCTGGCGGCAGGCTTAACACATGCAAGT
CGAACGATGACTCTCTAGCTTGCTAGAGATGATTAGTGGCGGACGGGTGAGTAACA
TTTAGGAATCTACCTAGTAGTGGGGGATAGCTCGGGGAAACTCGAATTAATACCGC
ATACGACCTACGGGTGAAAGGGGGCGCAAGCTCTTGCTATTAGATGAGCCTAAATC
AGATTAGCTAGTTGGTGGGGTAAAGGCCCACCAAGGCGACGATCTGTAACTGGTCT
GAGAGGATGATCAGTCACACCGGAACTGAGACACGGTCCGGACTCCTACGGGAGG
CAGCAGTGGGGAATATTGGACAATGGGGGCAACCCTGATCCAGCCATGCCGCGTG
TGTGAAGAAGGCCTTTTGGTTGTAAAGCACTTTAAGCAGGGAGGAGAGGCTAATG
GTTAATACCCATTAGATTAGACGTTACCTGCAGAATAAGCACCGGCTAACTCTGTG
CCAGCAGCCGCGGTAATACAGAGGGTGCGAGCGTTAATCGGAATTACTGGGCGTA
AAGCGAGTGTAGGTGGCTCATTAAGTCACATGTGAAATCCCCGGGCTTAACCTGGG
AACTGCATGTGATACTGGTGGTGCTAGAATATGTGAGAGGGAAGTAGAATTCCAG
GTGTAGCGGTGAAATGCGTAGAGATCTGGAGGAATACCGATGGCGAAGGCAGCTT
CCTGGCATAATATTGACACTGAGATTCGAAAGCGTGGGTAGCAAACAGGATTAGA
TACCCTGGTAGTCCACGCCGTAAACGATGTCTACTAGCCGTTGGGGTCCTTGAGAC
TTTAGTGGCGCAGTTAACGCGATAAGTAGACCGCCTGGGGAGTACGGCCGCAAGG
TTAAAACTCAAATGAATTGACGGGGGCCCGCACAAGCGGTGGAGCATGTGGTTTA
ATTCGATGCAACGCGAAGAACCTTACCTGGTCTTGACATAGTGAGAATCTTTCAGA
GATGAGAGAGTGCCTTCGGGAACTCACATACAGGTGCTGCATGGCTGTCGTCAGCT
CGTGTCGTGAGATGTTGGGTTAAGTCCCGCAACGAGCGCAACCCTTTTCCTTATTTG

CCAGCGGGTTAAGCCGGGAACTTTAAGGATACTGCCAGTGACAAACTGGAGGAAG
GCGGGGACGACGTCAAGTCATCATGGCCCTTACGACCAGGGCTACACACGTGCTAC
AATGGTAGGTACAGAGGGTTGCTACACAGCGATGTGATGCTAATCTCAAAAAGCCT
ATCGTAGTCCGGATTGGAGTCTGCAACTCGACTCCATGAAGTCGGAATCGCTAGTA
ATCGCGGATCAGAATGCCGCGGTGAATACGTTCCCGGGCCTTGTACACACCGCCCG
TCACACCATGGGAGTCTATTGCACCAGAAGTAGGTAGCCTAACGAAAGAGGGCGC
TTACCACGGTGTGGTCGATGACTGGGGTGAAGTCGTAACAAGGTAGCCGTAGGGG
AACCTGCGGTTGGATCACCTC
>Enhydrobacter aerosaccus G (NR029005)
ATTGAACGCTGGCGGCAGGCTTAACACATGCAAGTCGAACGATGACTCTCTAGCTT
GCTAGAGATGATTAGTGGCGGACGGGTGAGTAATATTTAGGAATCTGCCTAGTAGT
GGGGGATAGCTCGGGGAAACTCGAATTAATACCGCATACGACCTACGGGTGAAAG
GGGGCGCAAGMTCTTGCTATTAGATGAGCCTAAATCAGATTAGCTAGTTGGTGGG
GTAAAGGCCCACCAAGGCGACGATCTGTAACTGGTCTGAGAGGATGATCAGTCAC
ACCGGAACTGAGACACGGTCCGGACTCCTACGGGAGGCAGCAGTGGGGAATATTG
GACAATGGGGGCAACCCTGATCCAGCCATGCCGCGTGTGTGAAGAAGGCCTTTTGG
TTGTAAAGCACTTTAAGCAGGGAGGAGAGGCTAATGGTTAATACCCATTAGATTAG
ACGTTACCTGCAGAATAAGCACCGGCTAACTCTGTGCCAGCAGCCGCGGTAATACA
GAGGGTGCGAGCGTTAATCGGAATTACTGGGCGTAAAGCGAGTGTAGGTGGCTCA
TTAAGTCACATGTGAAATCCCCGGGCTTAACCTGGGAACTGCATGTGATACTGGTG
GTGCTAGAATATGTGAGAGGGAAGTAGAATTCCAGGTGTAGCGGTGAAATGCGTA
GAGATCTGGAGGAATACCGATGGCGAAGGCAGCTTCCTGGCATAATATTGACACT
GAGATTCGAAAGCGTGGGTAGCAAACAGGATTAGATACCCTGGTAGTCCACGCCG
TAAACGATGTCTACTAGCCGTTGGGGTCCTTGAGACTTTAGTGGCGCAGTTAACGC
GATAAGTAGACCGCCTGGGGAGTACGGCCGCAAGGTTAAAACTCAAATGAATTGA
CGGGGGCCCGCACAAGCGGTGGAGCATGTGGTTTAATTCGATGCAACGCGAAGAA
CCTTACCTGGTCTTGACATAGTGAGAATCTTTCAGAGATGAGAGAGTGCCTTCGGG
AATTCACATACAGGTGCTGCATGGCTGTCGTCAGCTCGTGTCGTGAGATGTTGGGT
TAAGTCCCGCAACGAGCGCAACCCTTTTCCTTATTTGCCAGCGGGTTAAGCCGGGA
ACTTTAAGGATACTGCCAGTGACAAACTGGAGGAAGGCGGGGACGACGTCAAGTC
ATCATGGCCCTTACGACCAGGGCTACACACGTGCTACAATGGTAGGTACAGAGGGT
TGCTACACAGCGATGTGATGCTAATCTCAAAAAGCCTATCGTAGTCCGGATTGGAG
TCTGCAACTCGACTCCATGAAGTCGGAATCGCTAGTAATCGCAGATCAGAATGCTG
CGGTGAATACGTTCCCGGGCCTTGTACACACCGCCCGTCACACCATGGGAGTCTAT
TGCACCAGAAGTAGGTAGCCTAACGAAAGAGGGCGCTTACCACGGTGTGGTCGAT
GACTGGGGTGAAGTCGTAACAAGGTAGCCGTAGGGGAACC
>Moraxella lincolnii LMG 5127 (NR117693)
ATTGAACGCTGGCGGCAGGCTTAACACATGCAAGTCGAACGAAGAGGTCTAGCTT
GCTAGACTGATTAGTGGCGAACGGGTGAGTAACATTTAGGAATCTACCTTATAGAG
GGGGATAGCTCGGGGAAACTCGAATTAATACCGCATACGACCTACGGGTGAAAGG
GGGCGCAAGCTCTTGCTATAAGATGAGCCTAAACCAGATTAGCTAGTTGGTGGGGT
AAAGGCCTACCAAGGCGACGATCTGTAGCTGGTCTGAGAGGATGATCAGCCACAC
TGGGACTGAGACACGGCCCAGACTCCTACGGGAGGCAGCAGTGGGGAATATTGGA
CAATGGGCGAAAGCCTGATCCAGCCATGCCGCGTGTGTGAAGAAGGCCTTATGGTT
GTAAAGCACTTTAAGTGGGGAGGAAAAGCTTATGGATAACACCCATAGGTTCTGA
CGTTACCCACAGAATAAGCACCGGCTAACTCTGTGCCAGCAGCCGCGGTAATACAG
AGGGTGCAAGCGTTAATCGGAATTACTGGGCGTAAAGCGAGCGTAGGTGGTTTATT
AAGTCAGATGTGAAATCCCTGGGCTTAACCTAGGAACTGCATCTGATACTGATAAA
CTAGAGTAGGTGAGAGGAAAGTAGAATTCCAGGTGTAGCGGTGAAATGCGTAGAG
ATCTGGAGGAATACCGATGGCGAAGGCAGCTTTCTGGCATCATACTGACACTGAGG
TTCGAAAGCGTGGGTAGCAAACAGGATTAGATACCCTGGTAGTCCACGCCGTAAA

CGATGTCTACCAGTCGTTGGGTCTTTTAAAGGCTTAGTGACGCAGTTAACGCGATA
AGTAGACCGCCTGGGGAGTACGGCCGCAAGGTTAAAACTCAAATGAATTGACGGG
GGCCCGCACAAGCGGTGGAGCATGTGGTTTAATTCGATGCAACGCGAAGAACCTT
ACCTGGTCTTGACATAGTGAGAATCTTGCAGAGATGCGAGAGTGCCTTTGGGAATT
CACATACAGGTGCTGCATGGCTGTCGTCAGCTCGTGTCGTGAGATGTTGGGTTAAG
TCCCGCAACGAGCGCAACCCTTTTCCTTAGTTACCAGCGACTTGGTCGGGGACTTT
AAGGACACTGCCAGTGACAAACTGGAGGAAGGCGGGGACGACGTCAAGTCATCAT
GGCCCTTACGACCAGGGCTACACACGTGCTACAATGGTAGGTACAGAGGGCAGCT
ACACGGCGACGTGATGCCAATCTCAAAAAGCCTATCTTAGTCCAGATTGGAGTCTG
CAACTCGACTCCATGAAGTCGGAATCGCTAGTAATCGCGGATCAGAATGCCGCGGT
GAATACGTTCCCGGGCCTTGTACACACCGCCCGTCACACCATGGGAGTTGATTGCA
CCAGAAGTGGTTAGCCTAACTTAGGAGGGCGATCACCACGGTGTGGTTAATGACTG
GGGTGAAGTCGTAACAAGGTAGCCGTAGGGGAACCTGCGGCTGGATCACCTCCTT
A
>Moraxella lincolnii CCUG 9405 (NR117189)
ATTGAACGCTGGCGGCAGGCTTAACACATGCAAGTCGAACGAAGAGGTCTAGCTT
GCTAGACTGATTAGTGGCGAACGGGTGAGTAACATTTAGGAATCTACCTTATAGAG
GGGGATAGCTCGGGGAAACTCGAATTAATACCGCATACGACCTACGGGTGAAAGG
GGGCGCAAGCTCTTGCTATAAGATGAGCCTAAACCAGATTAGCTAGTTGGTGGGGT
AAAGGCCTACCAAGGCGACGATCTGTAGCTGGTCTGAGAGGATGATCAGCCACAC
TGGGACTGAGACACGGCCCAGACTCCTACGGGAGGCAGCAGTGGGGAATATTGGA
CAATGGGCGAAAGCCTGATCCAGCCATGCCGCGTGTGTGAAGAAGGCCTTATGGTT
GTAAAGCACTTTTAAGTGGGGAGAAAAGCTTATGGATAACACCCATAGGTTCTGAC
GTTACCCACAGAATAAGCACCGGCTAACTCTGTGCCAGCAGCCGCGGTAATACAG
AGGGTGCAAGCGTTAATCGGAATTACTGGGCGTAAAGCGAGCGTAGGTGGTTTATT
AAGTCAGATGTGAAATCCCTGGGCTTAACCTAGGAACTGCATCTGATACTGATAAA
CTAGAGTAGGTGAGAGGAAAGTAGAATTCCAGGTGTAGCGGTGAAATGCGTAGAG
ATCTGGAGGAATACCGATGGCGAAGGCAGCTTTCTGGCATCATACTGACACTGAGG
TTCGAAAGCGTGGGTAGCAAACAGGATTAGATACCCTGGTAGTCCACGCCGTAAA
CGATGTCTACCAGTCGTTGGGTCTTTTAAAGGCTTAGTGACGCAGTTAACGCGATA
AGTAGACCGCCTGGGGAGTACGGCCGCAAGGTTAAAACTCAAATGAATTGACGGG
GGCCCGCACAAGCGGTGGAGCATGTGGTTTAATTCGATGCAACGCGAAGAACCTT
ACCTGGTCTTGACATAGTGAGAATCTTGCAGAGATGCGAGAGTGCCTTTGGGAATT
CACATACAGGTGCTGCATGGCTGTCGTCAGCTCGTGTCGTGAGATGTTGGGTTAAG
TCCCGCAACGAGCGCAACCCTTTTCCTTAGTTACCAGCGACTTGGTCGGGGACTTT
AAGGACACTGCCAGTGACAAACTGGAGGAAGGCGGGGACGACGTCAAGTCATCAT
GGCCCTTACGACCAGGGCTACACACGTGCTACAATGGTAGGTACAGAGGGCAGCT
ACACGGCGACGTGATGCCAATCTCAAAAAGCCTATCTTAGTCCAGATTGGAGTCTG
CAACTCGACTCCATGAAGTCGGAATCGCTAGTAATCGCGGATCAGAATGCCGCGGT
GAATACGTTCCCGGGCCTTGTACACACCGCCCGTCACACCATGGGAGTTGATTGCA
CCAGAAGTGGTTAGCCTAACTTAGGAGGGCGATCACCACGGTGTGGTTAATGACTG
GGGTGAAGTCGTAACAAGGTAGCCGTAGGGGAACCTGCGGCTGGATCACCTCCTT
A
>Chryseobacterium hominis NF802 (NR042517.2)
TTTGATCCCTGGCTCAGGATGAACGCTAGCGGGAGGCCTAACACATGCAAGCCGA
GCGGTATTTGTTCTTCGGAACAGAGAGAGCGGCGCACGGGTGCGGAACACGTGTG
CAACCTGCCTTTATCTGGGGGATAGCCTTTCGAAAGGAAGATTAATACCCCATAAT
ATATTGAGTGGCATCATTTGATATTGAAAACTCCGGTGGATAGAGATGGGCACGCG
CAAGATTAGATAGTTGGTGAGGTAACGGCTCACCAAGTCAATGATCTTTAGGGGGC
CTGAGAGGGTGATCCCCCACACTGGTACTGAGACACGGACCAGACTCCTACGGGA
GGCAGCAGTGAGGAATATTGGACAATGGGTTAGCGCCTGATCCAGCCATCCCGCGT

GAAGGACGACGGCCCTATGGGTTGTAAACTTCTTTTGTATAGGGATAAACCTACTC
TCGTGAGAGTAGCTGAAGGTACTATACGAATAAGCACCGGCTAACTCCGTGCCAGC
AGCCGCGGTAATACGGAGGGTGCAAGCGTTATCCGGATTTATTGGGTTTAAAGGGT
CCGTAGGCGGATCTGTAAGTCAGTGGTGAAATCTCACAGCTTAACTGTGAAACTGC
CATTGATACTGCAGGTCTTGAGTAAATTTGAAGTGGCTGGAATAAGTAGTGTAGCG
GTGAAATGCATAGATATTACTTAGAACACCAATTGCGAAGGCAGGTCACTAAGATT
TAACTGACGCTGATGGACGAAAGCGTGGGGAGCGAACAGGATTAGATACCCTGGT
AGTCCACGCCGTAAACGATGCTAACTCGTTTTTGGGGYTTCGGCTTCAGAGACTAA
GCGAAAGTGATAAGTTAGCCACCTGGGGAGTACGTTCGCAAGAATGAAACTCAAA
GGAATTGACGGGGGCCCGCACAAGCGGTGGATTATGTGGTTTAATTCGATGATACG
CGAGGAACCTTACCAAGACTTAAATGGGAAATGACAGATTTAGAAATAGATCCTTC
TTCGGACATTTTTCAAGGTGCTGCATGGTTGTCGTCAGCTCGTGCCGTGAGGTGTTA
GGTTAAGTCCTGCAACGAGCGCAACCCCTGTCACTAGTTGCTAGCATTAAGTTGAG
GACTCTAGTGAGACTGCCTACGCAAGTAGAGAGGAAGGTGGGGATGACGTCAAAT
CATCACGGCCCTTACGTCTTGGGCCACACACGTAATACAATGGCCGGTACAGAGGG
CAGCTACACAGCGATGTGATGCAAATCTCGAAAGCCGGTCTCAGTTCGGATTGGAG
TCTGCAACTCGACTCTATGAAGCTGGAATCGCTAGTAATCGCGCATCAGCCATGGC
GCGGTGAATACGTTCCCGGGCCTTGTACACACCGCCCGTCAAGCCATGGAAGTCTG
GGGTACCTGAAGTCGGTGACCGTAAAAGGAGCTGCCTAGGGTAAAACAGGTAACT
AGGGCTAAGTCGTAACAAGGTAGCCGTACCCGA
>Chryseobacterium hagamense RHA2-9 (NR115852)
CATGGCTCAGGATGAACGCTAGCGGGAGGCCTAACACATGCAAGCTGAGCGGTAT
TTGTCTTTCGGGACAGAGAGAGCGGCGTACGGGTGCGGAACACGTGTGCAACCTG
CCTTTATCTGGGGGATAGCCTTTCGAAAGGAAGATTAATACCCCATAACATATTGA
GTGGCATCACTTGATATTGAAAACTCCGGTGGATAGAGATGGGCACGCGCAAGATT
AGATAGTTGGTGAGGTAACGGCTCACCAAGTCGATGATCTTTAGGGGGCCTGAGA
GGGTGATCCCCCACACTGGTACTGAGACACGGACCAGACTCCTACGGGAGGCAGC
AGTGAGGAATATTGGACAATGGGTTAGCGCCTGATCCAGCCATCCCGCGTGAAGG
ACGACGGCCCTATGGGTTGTAAACTTCTTTTGTACAGGGATAAACCTACCCTCGTG
AGGGTAGCTGAAGGTACTGTACGAATAAGCACCGGCTAACTCCGTGCCAGCAGCC
GCGGTAATACGGAGGGTGCAAGCGTTATCCGGATTTATTGGGTTTAAAGGGTCCGT
AGGCGGATCTGTAAGTCAGTGGTGAAATCTCACAGCTTAACTGTGAAACTGCCATT
GATACTGCAGGTCTTGAGTAAGGTAGAAGTGGCTGGAATAAGTAGTGTAGCGGTG
AAATGCATAGATATTACTTAGAACACCAATTGCGAAGGCAGGTCACTATGTCTTAA
CTGACGCTGATGGACGAAAGCGTGGGGAGCGAACAGGATTAGATACCCTGGTAGT
CCACGCCGTAAACGATGCTAACTCGTTTTTGGGCTTTAGGGTTCAGAGACTAAGCG
AAAGTGATAAGTTAGCCACCTGGGGAGTACGTTCGCAAGAATGAAACTCAAAGGA
ATTGACGGGGGCCCGCACAAGCGGTGGATTATGTGGTTTAATTCGATGATACGCGA
GGAACCTTACCAAGGCTTAAATGGGAATTGATTGGTTTAGAAATAGACCGTCCTTC
GGGCAATTTTCAAGGTGCTGCATGGTTGTCGTCAGCTCGTGCCGTGAGGTGTTAGG
TTAAGTCCTGCAACGAGCGCAACCCCTGTTACTAGTTGCTACCATTAAGTTGAGGA
CTCTAGTAAGACTGCCTACGCAAGTAGAGAGGAAGGTGGGGATGACGTCAAATCA
TCACGGCCCTTACGCCTTGGGCCACACACGTAATACAATGGCCGGTACAGAGGGCA
GCTACCTAGCGATAGGATGCGAATCTCGAAAGCCGGTCTCAGTTCGGATTGGAGTC
TGCAACTCGACTCTATGAAGCTGGAATCGCTAGTAATCGCGCATCAGCCATGGCGC
GGTGAATACGTTCCCGGGCCTTGTACACACCGCCCGTCAAGCCATGGAAGTCTGGG
GTACCTGAAGTCGGTGACTCGTAAACAGGAGCT
>Chryseobacterium bovis H9 (NR044166)
TAGAGGTTTTATCCTGGCTCAGGATGAACGCTAGCGGGAGGCCTAACACATGCAAG
CCGAGCGGTAGAGTATCTTCGGATACTTGAGAGCGGCGCACGGGTGCGGAACACG
TGTGCAACCTGCCTTTATCTGGGGGATAGCCTTTCGAAAGGAAGATTAATACCCCA

TAATATATTTGATGGCATCATTAGATATTGAAAACTCCGGTGGATAGAGATGGGCA
CGCGCAAGATTAGTTAGTTGGTGAGGTAACGGCTCACCAAGGCGATGATCTTTAGG
GGGCCTGAGAGGGTGATCCCCCACACTGGTACTGAGACACGGACCAGACTCCTAC
GGGAGGCAGCAGTGAGGAATATTGGACAATGGGTTAGCGCCTGATCCAGCCATCC
CGCGTGAAGGACGACGGCCCTATGGGTTGTAAACTTCTTTTGTATAGGGATAAACC
TACTCTCGTGAGAGTAGCTGAAGGTACTATACGAATAAGCACCGGCTAACTCCGTG
CCAGCAGCCGCGGTAATACGGAGGGTGCAAGCGTTATCCGGATTTATTGGGTTTAA
AGGGTCCGTAGGCGGATCTGTAAGTCAGTGGTGAAATCTCATAGCTTAACTATGAA
ACTGCCATTGATACTGCAGGTCTTGAGTAAATTTGAAGTGGCTGGAATAAGTAGTG
TAGCGGTGAAATGCATAGATATTACTTAGAACACCAATTGCGAAGGCAGGTCACTA
AGATTTAACTGACGCTGATGGACGAAAGCGTGGGGAGCGAACAGGATTAGATACC
CTGGTAGTCCACGCCGTAAACGATGCTAACTCGTTTTTGTGATTCGTCATGAGAG
ACTAAGCGAAAGTGATAAGTTAGCCACCTGGGGAGTACGTTCGCAGGAATGAAAC
TCAAAGGAATTGACGGGGGCCCGCACAAGCGGTGGATTATGTGGTTTAATTCGATG
ATACGCGAGGAACCTTACCAAGACTTAAATGGGAGTGGACAGGCTTAGAAATAGG
CCCTCCTTCGGGCCGCTTCCAAGGTGCTGCATGGTTGTCGTCAGCTCGTGCCGTGAG
GTGTTAGGTTAAGTCCTGCAACGAGCGCAACCCCTGTCGCCAGTTGCTACCATTCA
GTTGAGGACTCTGGCGAGACTGCCTACGCAAGTAGAGAGGAAGGTGGGGATGACG
TCAAATCATCACGGCCCTTACGTCTTGGGCCACACACGTAATACAATGGCCGGTAC
AGAGGGCAGCTACACAGCGATGTGATGCAAATCTCGAAAGCCGGTCTCAGTTCGG
ATTGGAGTCTGCAACTCGACTCTATGAAGCTGGAATCGCTAGTAATCGCGCATCAG
CCATGGCGCGGTGAATACGTTCCCGGGCCTTGTACACACCGCCCGTCAAGCCATGG
AAGTCTGGGGTACCTGAAGTCGGTGACCGTAAAAGGAGCTGCCTAGGGTAAAACA
GGTAACTAGGGCTAAGTCGTAACAAGGTAGCCGATAT
>Chryseobacterium arthrosphaerae CC-VM-7 (NR116977)
GATGAACGCTAGCGGGAGGCCTAACACATGCAAGCCGAGCGGTAGAGATCTTTCG
GGATCTTGAGAGCGGCGTACGGGTGCGGAACACGTGTGCAACCTGCCTTTATCAGG
GGGATAGCCTTTCGAAAGGAAGATTAATACCCCATAATATATCGAGTGGCATCACT
TGATATTGAAAACTCCGGTGGATAGAGATGGGCACGCGCAAGATTAGATAGTTGG
TGAGGTAACGGCTCACCAAGTCTGCGATCTTTAGGGGGCCTGAGAGGGTGATCCCC
CACACTGGTACTGAGACACGGACCAGACTCCTACGGGAGGCAGCAGTGAGGAATA
TTGGACAATGGGTGCGAGCCTGATCCAGCCATCCCGCGTGAAGGATGACGGCCCTA
TGGGTTGTAAACTTCTTTTGTATAGGGATAAACCTACCCTCGTGAGGGTAGCTGAA
GGTACTATACGAATAAGCACCGGCTAACTCCGTGCCAGCAGCCGCGGTAATACGG
AGGGTGCAAGCGTTATCCGGATTTATTGGGTTTAAAGGGTCCGTAGGCGGATCTGT
AAGTCAGTGGTGAAATCTCACAGCTTAACTGTGAAACTGCCATTGATACTGCAGGT
CTTGAGTGTTGTTGAAGTAGCTGGAATAAGTAGTGTAGCGGTGAAATGCATAGATA
TTACTTAGAACACCAATTGCGAAGGCAGGTTACTAAGCAACAACTGACGCTGATGG
ACGAAAGCGTGGGGAGCGAACAGGATTAGATACCCTGGTAGTCCACGCCGTAAAC
GATGCTAACTCGTTTTTGGTATTTCGGTATCAGAGACTAAGCGAAAGTGATAAGTT
AGCCACCTGGGGAGTACGAACGCAAGTTTGAAACTCAAAGGAATTGACGGGGGCC
CGCACAAGCGGTGGATTATGTGGTTTAATTCGATGATACGCGAGGAACCTTACCAA
GGCTTAAATGGGAAATGACAGGCTTAGAAATAGGCTTTTCTTCGGACATTTTTCAA
GGTGCTGCATGGTTGTCGTCAGCTCGTGCCGTGAGGTGTTAGGTTAAGTCCTGCAA
CGAGCGCAACCCCTGTCACTAGTTGCCATCATTAAGTTGGGGACTCTAGTGAGACT
GCCTACGCAAGTAGAGAGGAAGGTGGGGATGACGTCAAATCATCACGGCCCTTAC
GCCTTGGGCCACACACGTAATACAATGGCCGGTACAGAGGGCAGCTACACAGCGA
TGTGATGCAAATCTCGAAAGCCGGTCTCAGTTCGGATTGGAGTCTGCAACTCGACT
CTATGAAGCTGGAATCGCTAGTAATCGCGCATCAGCCATGGCGCGGTGAATACGTT
CCCGGGCCTTGTACACACCGCCCGTCAA
>Acinetobacter johnsonii ATCC 17909 (NR117624)

AGAGTTTGATCATGGCTCAGATTGAACGCTGGCGGCAGGCTTAACACATGCAAGTC
GAGCGGGGAAAGGTAGCTTGCTACCTAACCTAGCGGCGGACGGGTGAGTAATGCT
TAGGAATCTGCCTATTAGTGGGGGACAACATTCCGAAAGGAATGCTAATACCGCAT
ACGCCCTACGGGGGAAAGCAGGGGATCTTCGGACCTTGCGCTAATAGATGAGCCT
AAGTCAGATTAGCTAGTTGGTGGGGTAAAGGCCTACCAAGGCGACGATCTGTAGC
GGGTCTGAGAGGATGATCCGCCACACTGGGACTGAGACACGGCCCAGACTCCTAC
GGGAGGCAGCAGTGGGGAATATTGGACAATGGGCGAAAGCCTGATCCAGCCATGC
CGCGTGTGTGAAGAAGGCCTTTTGGTTGTAAAGCACTTTAAGCGAGGAGGAGGCTA
CTTGGATTAATACTCTAGGATAGTGGACGTTACTCGCAGAATAAGCACCGGCTAAC
TCTGTGCCAGCAGCCGCGGTAATACAGAGGGTGCGAGCGTTAATCGGATTTACTGG
GCGTAAAGCGTGCGTAGGCGGCTTTTTAAGTCGGATGTGAAATCCCTGAGCTTAAC
TTAGGAATTGCATTCGATACTGGGAAGCTAGAGTATGGGAGAGGATGGTAGAATT
CCAGGTGTAGCGGTGAAATGCGTAGAGATCTGGAGGAATACCGATGGCGAAGGCA
GCCATCTGGCCTAATACTGACGCTGAGGTACGAAAGCATGGGGAGCAAACAGGAT
TAGATACCCTGGTAGTCCATGCCGTAAACGATGTCTACTAGCCGTTGGGGCCTTTG
AGGCTTTAGTGGCGCAGCTAACGCGATAAGTAGACCGCCTGGGGAGTACGGTCGC
AAGACTAAAACTCAAATGAATTGACGGGGGCCCGCACAAGCGGTGGAGCATGTGG
TTTAATTCGATGCAACGCGAAGAACCTTACCTGGTCTTGACATAGTAAGAACTTTC
CAGAGATGGATTGGTGCCTTCGGGAACTTACATACAGGTGCTGCATGGCTGTCGTC
AGCTCGTGTCGTGAGATGTTGGGTTAAGTCCCGCAACGAGCGCAACCCTTTTCCTT
ATTTGCCAGCGGGTTAAGCCGGGAACTTTAAGGATACTGCCAGTGACAAACTGGA
GGAAGGCGGGGACGACGTCAAGTCATCATGGCCCTTACGACCAGGGCTACACACG
TGCTACAATGGTCGGTACAAAGGGTTGCTACCTAGCGATAGGATGCTAATCTCAAA
AAGCCGATCGTAGTCCGGATTGGAGTCTGCAACTCGACTCCATGAAGTCGGAATCG
CTAGTAATCGCGGATCAGAATGCCGCGGTGAATACGTTCCCGGGCCTTGTACACAC
CGCCCGTCACACCATGGGAATTTGTTGCACCAGAAGTAGGTAGTCTAACCGCAAGG
AGGACGCTTACCACGGTGTGGCCGATGACTGGGGTGAAGTCGTAACAAGGTAGCC
GTAGGGGAACCTGCGGCTGGATCACCTCCTT
>Acinetobacter johnsonii Mannheim 3865/60 (NR044975)
CCTGGCTCAGATTGAACGCTGGCGGCAGGCTTAACACATGCAAGTCGAGCGGGGA
GAGGTAGCTTGCTACCTAACCTAGCGGCGGACGGGTGAGTAATGCTTAGGAATCTG
CCTATTAGTGGGGGACAACATTCCGAAAGGAATGCTAATACCGCATACGTCCTACG
GGNGAAAGCAGGGGATCTTCGGACCTTGCGCTAATAGATGAGCCTAAGTCAGATT
AGCTAGTTGGTGGGGTAAAGGCCTACCAAGGCGACGATCTGTAGCGGGTCTGAGA
GGATGATCCGCCACACTGGGACTGAGACACGGCCCAGACTCCTACGGGAGGCAGC
AGTGGGGAATATTGGACAATGGGCGGAAGCCTGATCCAGCCATGCCGCGTGTGTG
AAGAAGGCCTTTTGGTTGTAAAGCACTTTAAGCGAGGAGGAGGCTACTTGGATTAA
TACTCTAGGATAGTGGACGTTACTCGCAGAATAAGCACCGGCTAACTCTGTGCCAG
CAGCCGCGGTAATACAGAGGGTGCGAGCGTTAATCGGATTTACTGGGCGTAAAGC
GTGCGTAGGCGGCTTTTTAAGTCGGATGTGAAATCCCTGAGCTTAACTTAGGAATT
GCATTCGATACTGGGAAGCTAGAGTATGGGAGAGGATGGTAGAATTCCAGGTGTA
GCGGTGAAATGCGTAGAGATCTGGAGGAATACCGATGGCGAAGGCAGCCATCTGG
CCTAATACTGACGCTGAGGTACGAAAGCATGGGGAGCAAACAGGATTAGATACCC
TGGTAGTCCATGCCGTAAACGATGTCTACTAGCCGTTGGGGCCTTTGAGGCTTTAG
TGGCGCAGCTAACGCGATAAGTAGACCGCCTGGGGAGTACGGTCGCAAGACTAAA
ACTCAAATGAATTGACGGGGGCCCGCACAAGCGGTGGAGCATGTGGTTTAATTCG
ATGCAACGCGAAGAACCTTACCTGGTCTTGACATAGTAAGAACTTTCCAGAGATGG
ATTGGTGCCTTCGGGAACTTACATACAGGTGCTGCATGGCTGTCGTCAGCTCGTGT
CGTGAGATGTTGGGTTAAGTCCCGCAACGAGCGCAACCCTTTTCCTTATTTGCCAG
CGGGTTAAGCCGGGAACTTTAAGGATACTGCCAGTGACAAACTGGAGGAAGGCGG
GGACGACGTCAAGTCATCATGGCCCTTACGACCAGGGCTACACACGTGCTACAATG

GTCGGTACAAAGGGTTGCTACCTAGCGATAGGATGCTAATCTCAAAAAGCCGATCG
TAGTCCGGATTGGAGTCTGCAACTCGACTCCATGAAGTCGGAATCGCTAGTAATCG
CGGATCAGAATGCCGCGGTGAATACGTTCCCGGGNCTTGTACACACCGCCCGTCAC
ACCATGGGAGTTTGTTGCACCAGAAGTAG
>Acinetobacter haemolyticus ATCC 17906 (NR117622)
AGAGTTTGTTCATGGCTCAGATTGAACGCTGGCGGCAGGCTTAACACATGCAAGTC
GAGCGGGGAAGGGTAGCTTGCTACCTAACCTAGCGGCGGACGGGTGAGTAATGCT
TAGGAATCTGCCTATTAGTGGGGGACAACATTCCGAAAGGAATGCTAATACCGCAT
ACGTCCTACGGGAGAAAGCAGGGGATCTTCGGACCTTGCGCTAATAGATGAGCCT
AAGTCGGATTAGCTAGTTGGTGGGGTAAAGGCCTACCAAGGCGACGATCTGTAGC
GGGTCTGAGAGGATGATCCGCCACACTGGGACTGAGACACGGCCCAGACTCCTAC
GGGAGGCAGCAGTGGGGAATATTGGACAATGGGCGGAAGCCTGATCCAGCCATGC
CGCGTGTGTGAAGAAGGCCTTTTGGTTGTAAAGCACTTTAAGCGAGGAGGAGGCTA
CTCTAGTTAATACCTAGAGATAGTGGACGTTACTCGCAGAATAAGCACCGGCTAAC
TCTGTGCCAGCAGCCGCGGTAATACAGAGGGTGCGAGCGTTAATCGGATTTACTGG
GCGTAAAGCGTGCGTAGGCGGCTGATTAAGTCGGATGTGAAATCCCTGAGCTTAAC
TTAGGAATTGCATTCGATACTGGTCAGCTAGAGTATGGGAGAGGATGGTAGAATTC
CAGGTGTAGCGGTGAAATGCGTAGAGATCTGGAGGAATACCGATGGCGAAGGCAG
CCATCTGGCCTAATACTGACGCTGAGGTACGAAAGCATGGGGAGCAAACAGGATT
AGATACCCTGGTAGTCCATGCCGTAAACGATGTCTACTAGCCGTTGGGGCCTTTGA
GGCTTTAGTGGCGCAGCTAACGCGATAAGTAGACCGCCTGGGGAGTACGGTCGCA
AGACTAAAACTCAAATGAATTGACGGGGGCCCGCACAAGCGGTGGAGCATGTGGT
TTAATTCGATGCAACGCGAAGAACCTTACCTGGTCTTGACATAGTAAGAACTTTCC
AGAGATGGATTGGTGCCTTCGGGAACTTACATACAGGTGCTGCATGGCTGTCGTCA
GCTCGTGTCGTGAGATGTTGGGTTAAGTCCCGCAACGAGCGCAACCCTTTTCCTTAT
TTGCCAGCGGGTTAAGCCGGGAACTTTAAGGATACTGCCAGTGACAAACTGGAGG
AAGGCGGGGACGACGTCAAGTCATCATGGCCCTTACGACCAGGGCTACACACGTG
CTACAATGGTCGGTACAAAGGGTTGCTACCTAGCGATAGGATGCTAATCTCAAAAA
GCCGATCGTAGTCCGGATTGGAGTCTGCAACTCGACTCCATGAAGTCGGAATCGCT
AGTAATCGCGGATCAGAATGCCGCGGTGAATACGTTCCCGGGCCTTGTACACACCG
CCCGTCACACCATGGGAGTTTGTTGCACCAGAAGTAGGTAGTCTAACCGTAAGGAG
GACGCTTACCACGGTGTGGCCGATGACTGGGGTGAAGTCGTAACAAGGTAGCCGT
AGGGGAACCTGCGGCTGGATCACCTCCTT
>Acinetobacter oleivorans DR1 (NR102814)
AACTGAAGAGTTTGATCATGGCTCAGATTGAACGCTGGCGGCAGGCTTAACACATG
CAAGTCGAGCGGAGAGAGGTAGCTTGCTACTGATCTTAGCGGCGGACGGGTGAGT
AATGCTTAGGAATCTGCCTATTAGTGGGGGACAACATTTCGAAAGGAATGCTAATA
CCGCATACGTCCTACGGGAGAAAGCAGGGGATCTTCGGACCTTGCGCTAATAGATG
AGCCTAAGTCGGATTAGCTAGTTGGTGGGGTAAAGGCCTACCAAGGCGACGATCT
GTAGCGGGTCTGAGAGGATGATCCGCCACACTGGGACTGAGACACGGCCCAGACT
CCTACGGGAGGCAGCAGTGGGGAATATTGGACAATGGGCGGAAGCCTGATCCAGC
CATGCCGCGTGTGTGAAGAAGGCCTTATGGTTGTAAAGCACTTTAAGCGAGGAGG
AGGCTACTTTAGTTAATACCTAGAGATAGTGGACGTTACTCGCAGAATAAGCACCG
GCTAACTCTGTGCCAGCAGCCGCGGTAATACAGAGGGTGCAAGCGTTAATCGGATT
TACTGGGCGTAAAGCGCGCGTAGGCGGCTAATTAAGTCAAATGTGAAATCCCCGA
GCTTAACTTGGGAATTGCATTCGATACTGGTTAGCTAGAGTGTGGGAGAGGATGGT
AGAATTCCAGGTGTAGCGGTGAAATGCGTAGAGATCTGGAGGAATACCGATGGCG
AAGGCAGCCATCTGGCCTAACACTGACGCTGAGGTGCGAAAGCATGGGGAGCAAA
CAGGATTAGATACCCTGGTAGTCCATGCCGTAAACGATGTCTACTAGCCGTTGGGG
CCTTTGAGGCTTTAGTGGCGCAGCTAACGCGATAAGTAGACCGCCTGGGGAGTACG
GTCGCAAGACTAAAACTCAAATGAATTGACGGGGGCCCGCACAAGCGGTGGAGCA

TGTGGTTTAATTCGATGCAACGCGAAGAACCTTACCTGGCCTTGACATAGTAAGAA
CTTTCCAGAGATGGATTGGTGCCTTCGGGAACTTACATACAGGTGCTGCATGGCTG
TCGTCAGCTCGTGTCGTGAGATGTTGGGTTAAGTCCCGCAACGAGCGCAACCCTTT
TCCTTATTTGCCAGCGAGTAATGTCGGGAACTTTAAGGATACTGCCAGTGACAAAC
TGGAGGAAGGCGGGGACGACGTCAAGTCATCATGGCCCTTACGGCCAGGGCTACA
CACGTGCTACAATGGTCGGTACAAAGGGTTGCTACCTAGCGATAGGATGCTAATCT
CAAAAAGCCGATCGTAGTCCGGATTGGAGTCTGCAACTCGACTCCATGAAGTCGGA
ATCGCTAGTAATCGCGGATCAGAATGCCGCGGTGAATACGTTCCCGGGCCTTGTAC
ACACCGCCCGTCACACCATGGGAGTTTGTTGCACCAGAAGTAGCTAGCCTAACTGC
AAAGAGGGCGGTTACCACGGTGTGGCCGATGACTGGGGTGAAGTCGTAACAAGGT
AGCCGTAGGGGAACCTGCGGCTGGATCACCTCCTTA
>Acinetobacter pittii ATCC 19004 (NR117621)
AGAGTTTGATCATGGCTCAGATTGAACGCTGGCGGCAGGCTTAACACATGCAAGTC
GAGCGGAGAGAGGTAGCTTGCTACTGATCTTAGCGGCGGACGGGTGAGTAATGCT
TAGGAATCTGCCTATTAGTGGGGGACAACATTTCGAAAGGAATGCTAATACCGCAT
ACGTCCTACGGGAGAAAGCAGGGGATCTTCGGACCTTGCGCTAATAGATGAGCCT
AAGTCGGATTAGCTAGTTGGTGGGGTAAAGGCCTACCAAGGCGACGATCTGTAGC
GGGTCTGAGAGGATGATCCGCCACACTGGGACTGAGACACGGCCCAGACTCCTAC
GGGAGGCAGCAGTGGGGAATATTGGACAATGGGCGCAAGCCTGATCCAGCCATGC
CGCGTGTGTGAAGAAGGCCTTATGGTTGTAAAGCACTTTAAGCGAGGAGGAGGCT
ACTTTAGTTAATACCTAGAGATAGTGGACGTTACTCGCAGAATAAGCACCGGCTAA
CTCTGTGCCAGCAGCCGCGGTAATACAGAGGGTGCAAGCGTTAATCGGATTTACTG
GGCGTAAAGCGCGCGTAGGCGGCTAATTAAGTCAAATGTGAAATCCCCGAGCTTA
ACTTGGGAATTGCATTCGATACTGGTTAGCTAGAGTGTGGGAGAGGATGGTAGAAT
TCCAGGTGTAGCGGTGAAATGCGTAGAGATCTGGAGGAATACCGATGGCGAAGGC
AGCCATCTGGCCTAACACTGACGCTGAGGTGCGAAAGCATGGGGAGCAAACAGGA
TTAGATACCCTGGTAGTCCATGCCGTAAACGATGTCTACTAGCCGTTGGGGCCTTT
GAGGCTTTAGTGGCGCAGCTAACGCGATAAGTAGACCGCCTGGGGAGTACGGTCG
CAAGACTAAAACTCAAATGAATTGACGGGGGCCCGCACAAGCGGTGGAGCATGTG
GTTTAATTCGATGCAACGCGAAGAACCTTACCTGGCCTTGACATAGTAAGAACTTT
CCAGAGATGGATTGGTGCCTTCGGGAACTTACATACAGGTGCTGCATGGCTGTCGT
CAGCTCGTGTCGTGAGATGTTGGGTTAAGTCCCGCAACGAGCGCAACCCTTTTCCT
TATTTGCCAGCGAGTAATGTCGGGAACTTTAAGGATACTGCCAGTGACAAACTGGA
GGAAGGCGGGGACGACGTCAAGTCATCATGGCCCTTACGGCCAGGGCTACACACG
TGCTACAATGGTCGGTACAAAGGGTTGCTACCTAGCGATAGGATGCTAATCTCAAA
AAGCCGATCGTAGTCCGGATTGGAGTCTGCAACTCGACTCCATGAAGTCGGAATCG
CTAGTAATCGCGGATCAGAATGCCGCGGTGAATACGTTCCCGGGCCTTGTACACAC
CGCCCGTCACACCATGGGAGTTTGTTGCACCAGAAGTAGCTAGCCTAACTGCAAAG
AGGGCGGTTACCACGGTGTGGCCGATGACTGGGGTGAAGTCGTAACAAGGTAGCC
GTAGGGGAACCTGCGGCTGGATCACCTCCTT
>Acinetobacter baumannii DSM 30007 (NR117677)
ATTGAACGCTGGCGGCAGGCTTAACACATGCAAGTCGAGCGGGGGAAGGTAGCTT
GCTACCGGACCTAGCGGCGGACGGGTGAGTAATGCTTAGGAATCTGCCTATTAGTG
GGGGACAACATCTCGAAAGGGATGCTAATACCGCATACGTCCTACGGGAGAAAGC
AGGGGATCTTCGGACCTTGCGCTAATAGATGAGCCTAAGTCGGATTAGCTAGTTGG
TGGGGTAAAGGCCTACCAAGGCGACGATCTGTAGCGGGTCTGAGAGGATGATCCG
CCACACTGGGACTGAGACACGGCCCAGACTCCTACGGGAGGCAGCAGTGGGGAAT
ATTGGACAATGGGGGGAACCCTGATCCAGCCATGCCGCGTGTGTGAAGAAGGCCT
TATGGTTGTAAAGCACTTTAAGCGAGGAGGAGGCTACTTTAGTTAATACCTAGAGA
TAGTGGACGTTACTCGCAGAATAAGCACCGGCTAACTCTGTGCCAGCAGCCGCGGT
AATACAGAGGGTGCGAGCGTTAATCGGATTTACTGGGCGTAAAGCGTGCGTAGGC

GGCTTATTAAGTCGGATGTGAAATCCCCGAGCTTAACTTGGGAATTGCATTCGATA
CTGGTGAGCTAGAGTATGGGAGAGGATGGTAGAATTCCAGGTGTAGCGGTGAAAT
GCGTAGAGATCTGGAGGAATACCGATGGCGAAGGCAGCCATCTGGCCTAATACTG
ACGCTGAGGTACGAAAGCATGGGGAGCAAACAGGATTAGATACCCTGGTAGTCCA
TGCCGTAAACGATGTCTACTAGCCGTTGGGGCCTTTGAGGCTTTAGTGGCGCAGCT
AACGCGATAAGTAGACCGCCTGGGGAGTACGGTCGCAAGACTAAAACTCAAATGA
ATTGACGGGGGCCCGCACAAGCGGTGGAGCATGTGGTTTAATTCGATGCAACGCG
AAGAACCTTACCTGGCCTTGACATACTAGAAACTTTCCAGAGATGGATTGGTGCCT
TCGGGAATCTAGATACAGGTGCTGCATGGCTGTCGTCAGCTCGTGTCGTGAGATGT
TGGGTTAAGTCCCGCAACGAGCGCAACCCTTTTCCTTACTTGCCAGCATTTCGGATG
GGAACTTTAAGGATACTGCCAGTGACAAACTGGAGGAAGGCGGGGACGACGTCAA
GTCATCATGGCCCTTACGGCCAGGGCTACACACGTGCTACAATGGTCGGTACAAAG
GGTTGCTACACAGCGATGTGATGCTAATCTCAAAAAGCCGATCGTAGTCCGGATTG
GAGTCTGCAACTCGACTCCATGAAGTCGGAATCGCTAGTAATCGCGGATCAGAATG
CCGCGGTGAATACGTTCCCGGGCCTTGTACACACCGCCCGTCACACCATGGGAGTT
TGTTGCACCAGAAGTAGCTAGCCTAACTGCAAAGAGGGCGGTTACCACGGTGTGG
CCGATGACTGGGGTGAAGTCGTAACAAGGTAGCCGTAGGGGAACCTGCGGCTGGA
TCACCTCCTTTCTATA
>Acinetobacter junii Mannheim 2723/59 (NR119360)
CCTGGCTCAGATTGAACGCTGGCGGCAGGCTTAACACATGCAAGTCGAGCGGAGA
TGAGGTGCTTGCACCTTATCTTAGCGGCGGACGGGTGAGTAATGCTTAGGAATCTG
CCTATTAGTGGGGGACAACATTCCGAAAGGAATGCTAATACCGCATACGTCCTACG
GGAGAAAGCAGGGGATCTTCGGACCTTGCGCTAATAGATGAGCCTAAGTCGGATT
AGCTAGTTGGTGGGGTAAAGGCCTACCAAGGCGACGATCTGTAGCGGGTCTGAGA
GGATGATCCGCCACACTGGGACTGAGACACGGCCCAGACTCCTACGGGAGGCAGC
AGTGGGGAATATTGGACAATGGGGGGAACCCTGATCCANCCATGCCGCGTGTGTG
AAGAAGGCCTTATGGTTGTAAAGCACTTTAAGCGAGGAGGAGGCTACTGAGACTA
ATACTCTTGGATAGTGGACGTTACTCGCAGAATAAGCACCGGCTAACTCTGTGCCA
GCAGCCGCGGTAATACAGAGGGTGCGAGCGTTAATCGGATTTACTGGGCGTAAAG
CGTGCGTAGGCGGCTTTTTAAGTCGGATGTGAAATCCCCGAGCTTAACTTGGGAAT
TGCATTCGATACTGGGAAGCTAGAGTATGGGAGAGGATGGTAGAATTCCAGGTGT
AGCGGTGAAATGCGTAGAGATCTGGAGGAATACCGATGGCGAAGGCAGCCATCTG
GCCTAATACTGACGCTGAGGTACGAAAGCATGGGGAGCAAACAGGATTAGATACC
CTGGTAGTCCATGCCGTAAACGATGTCTACTAGCCGTTGGGGCCTTTGAGGCTTTA
GTGGCGCAGCTAACGCGATAAGTAGACCGCCTGGGGAGTACGGTCGCAAGACTAA
AACTCAAATGAATTGACGGGGGCCCGCACAAGCGGTGGAGCATGTGGTTTAATTC
GATGCAACGCGAAGAACCTTACCTGGCCTTGACATACTAGAAACTTTCCAGAGATG
GATTGGTGCCTTCGGGAATCTAGATACAGGTGCTGCATGGCTGTCGTCAGCTCGTG
TCGTGAGATGTTGGGTTAAGTCCCGCAACGAGCGCAACCCTTTTCCTTACTTGCCA
GCATTTCGGATGGGAACTTTAAGGATACTGCCAGTGACAAACTGGAGGAAGGCGG
GGACGACGTCAAGTCATCATGGCCCTTACGGCCAGGGCTACACACGTGCTACAATG
GTCGGTACAAAGGGTTGCTACACAGCGATGTGATGCTAATCTCAAAAAGCCGATCG
TAGTCCGGATTGGAGTCTGCAACTCGACTCCATGAAGTCGGAATCGCTAGTAATCG
CGGATCAGAATGCCGCGGTGAATACGTTCCCGGGCCTTGTACACACCGCCCGTCAC
ACCATGGGAGTTTGTTGCACCAGAAGTAG
>Acinetobacter septicus AK001 (NR116071)
AGAGTTTGATCCTGGCTCAGATTGAACGCTGGCGGCAGGCTTAACACATGCAAGTC
GAGCGGAGATGAGGTGCTTGCACCTTATCTTAGCGGCGGACGGGTGAGTAATACTT
AGGAATCTGGCTATTAGTGGGGGACAACATTTCGAAAGGGATGCTAATACCGCAT
ACGTCCTACGGGAGAAAGCAGGGGATCTTCGGACCTTGCGCTAATAGATGAGCCT
AAGTCGGATTAGCTAGTTGGTGGGGTAAAGGCCTACCAAGGCGACGATCTGTAGC

GGGTCTGAGAGGATGATCCGCCACACTGGGACTGAGACACGGCCCAGACTCCTAC
GGGAGGCAGCAGTGGGGAATATTGGACAATGGGGGAACCCTGATCCAGCCATGC
CGCGTGTGTGAAGAAGGCCTTATGGTTGTAAAGCACTTTAAGCGAGGAGGAGGGT
ACTGGTATTAATACTACCAGCTACTGGACGTTACTCGCAGAATAAGCACCGGCTAA
CTCTGTGCCAGCAGCCGCGGTAATACAGAGGGTGCGAGCGTTAATCGGATTTACTG
GGCGTAAAGCGTGCGTAGGCGGCTAATTGAGTCGGATGTGAAATCCCCGAGCTTA
ACTTGGGAATTGCATTCGATACTGGTTAGCTAGAGTGTGGGAGAGGATGGTAGAAT
TCCAGGTGTAGCGGTGAAATGCGTAGAGATCTGGAGGAATACCGATGGCGAAGGC
AGCCATCTGGCCTAACACTGACGCTGAGGTACGAAAGCATGGGGAGCAAACAGGA
TTAGATACCCTGGTAGTCCATGCCGTAAACGATGTCTACTAGCCGTTGGTCTCTTTG
AGGGATTAGTGGCGCAGCTAACGCGATAAGTAGACCGCCTGGGGAGTACGGTCGC
AAGACTAAAACTCAAATGAATTGACGGGGGCCCGCACAAGCGGTGGAGCATGTGG
TTTAATTCGATGCAACGCGAAGAACCTTACCTGGTCTTGACATAGTAAGAACTTTC
CAGAGATGGATTGGTGCCTTCGGGAACTTACATACAGGTGCTGCATGGCTGTCGTC
AGCTCGTGTCGTGAGATGTTGGGTTAAGTCCCGCAACGAGCGCAACCCTTTTCCTT
ATTTGCCAGCACTTTGGGTGGGAACTTTAAGGATACTGCCAGTGACAAACTGGAGG
AAGGCGGGGACGACGTCAAGTCATCATGGCCCTTACGACCAGGGCTACACACGTG
CTACAATGGTCGGTACAAAGGGTTGCTACCTAGCGATAGGATGCTAATCTCAAAAA
GCCGATCGTAGTCCGGATTGGAGTCTGCAACTCGACTCCATGAAGTCGGAATCGCT
AGTAATCGCGGATCAGAATGCCGCGGTGAATACGTTCCCGGGCCTTGTACACACCG
CCCGTCACACCATGGGAGTTTGTTGCACCAGAAGTGGATAGTCTAACTGCAAAGAG
GACGTTCACCACGGTGTGGCCGATGACTGGGGTGAAGTCGTAACAAGGTAGCCGT
AGGGGAACCTGCGGCTGGATCACCTCCTT
>Brevundimonas diminuta NBRC 12697 (NR113602)
AGCGAACGCTGGCGGCAGGCCTAACACATGCAAGTCGAACGGACCCTTCGGGGTT
AGTGGCGGACGGGTGAGTAACACGTGGGAACGTGCCTTTAGGTTCGGAATAGCTC
CTGGAAACGGGTGGTAATGCCGAATGTGCCCTTCGGGGGAAAGATTTATCGCCTTT
AGAGCGGCCCGCGTCTGATTAGCTAGTTGGTGAGGTAACGGCTCACCAAGGCGAC
GATCAGTAGCTGGTCTGAGAGGATGACCAGCCACACTGGGACTGAGACACGGCCC
AGACTCCTACGGGAGGCAGCAGTGGGGAATCTTGCGCAATGGGCGAAAGCCTGAC
GCAGCCATGCCGCGTGAATGATGAAGGTCTTAGGATTGTAAAATTCTTTCACCGGN
GACGATAATGACGGTACCCGGAGAAGAAGCCCCGGCTAACTTCGTGCCAGCAGCC
GCGGTAATACGAAGGGGGCTAGCGTTGCTCGGAATTACTGGGCGTAAAGGGCGCG
TAGGCGGATCGTTAAGTCAGAGGTGAAATCCCAGGGCTCAACCCTGGAACTGCCTT
TGATACTGGCGATCTTGAGTATGAGAGAGGTATGTGGAACTCCGAGTGTAGAGGTG
AAATTCGTAGATATTCGGAAGAACACCAGTGGCGAAGGCGACATACTGGCTCATT
ACTGACGCTGAGGCGCGAAAGCGTGGGGAGCAAACAGGATTAGATACCCTGGTAG
TCCACGCCGTAAACGATGATTGCTAGTTGTCGGGCTGCATGCAGTTCGGTGACGCA
GCTAACGCATTAAGCAATCCGCCTGGGGAGTACGGTCGCAAGATTAAAACTCAAA
GGAATTGACGGGGGCCCGCACAAGCGGTGGAGCATGTGGTTTAATTCGAAGCAAC
GCGCAGAACCTTACCACCTTTTGACATGCCTGGACCGCCACGGAGACGTGGCTTTC
CCTTCGGGGACTAGGACACAGGTGCTGCATGGCTGTCGTCAGCTCGTGTCGTGAGA
TGTTGGGTTAAGTCCCGCAACGAGCGCAACCCTCGCCATTAGTTGCCATCATTTAG
TTGGGAACTCTAATGGGACTGCCGGTGCTAAGCCGGAGGAAGGTGGGGATGACGT
CAAGTCCTCATGGCCCTTACAGGGTGGGCTACACACGTGCTACAATGGCAACTACA
GAGGGTTAATCCTTAAAAGTTGTCTCAGTTCGGATTGTCCTCTGCAACTCGAGGGC
ATGAAGTTGGAATCGCTAGTAATCGCGGATCAGCATGCCGCGGTGAATACGTTCCC
GGGCCTTGTACACACCGCCCGTCACACCATGGGAGTTGGTTCTACCCGAAGGCGGT
GCGCTAACCAGCAATGGAGGCAGCCGACCACGGTAGGGTCAGCGACTGGGGTGAA
G
>Brevundimonas diminuta LMG 2089 (NR114708)

AGCGAACGCTGGCGGCAGGCCTAACACATGCAAGTCGAACGGACCCTTCGGGGTT
AGTGGCGGACGGGTGAGTAACACGTGGGAACGTGCCTTTAGGTTCGGAATAGCTC
CTGGAAACGGGTGGTAATGCCGAATGTGCCCTTCGGGGGAAAGATTTATCGCCTTT
AGAGCGGCCCGCGTCTGATTAGCTAGTTGGTGAGGTAACGGCTCACCAAGGCGAC
GATCAGTAGCTGGTCTGAGAGGATGACCAGCCACACTGGGACTGAGACACGGCCC
AGACTCCTACGGGAGGCAGCAGTGGGGAATCTTGCGCAATGGGCGAAAGCCTGAC
GCAGCCATGCGGCGTGAATGATGAAGGTCTTAGGATTGTAAAATTCTTTCACCGGG
GACGATAATGACGGTACCCGGAGAAGAAGCCCCGGCTAACTTCGTGCCAGCAGCC
GCGGTAATACGAAGGGGGCTAGCGTTGCTCGGAATTACTGGGCGTAAAGGGCGCG
TAGGCGGATCGTTAAGTCAGAGGTGAAATCCCAGGGCTCAACCCTGGAACTGCCTT
TGATACTGGCGATCTTGAGTATGAGAGAGGTATGTGGAACTCCGAGTGTAGAGGTG
AAATTCGTAGATATTCGGAAGAACACCAGTGGCGAAGGCGACATACTGGCTCATT
ACTGACGCTGAGGCGCGAAAGCGTGGGGAGCAAACAGGATTAGATACCCTGGTAG
TCCACGCCGTAAACGATGATTGCTAGTTGTCGGGCTGCATGCAGTTCGGTGACGCA
GCTAACGCATTAAGCAATCCGCCTGGGGAGTACGGTCGCAAGATTAAAACTCAAA
GGAATTGACGGGGGCCCGCACAAGCGGTGGAGCATGTGGTTTAATTCGAAGCAAC
GCGCAGAACCTTACCACCTTTTGACATGCCTGGACCGCCACGGAGACGTGGCTTTC
CCTTCGGGGACTAGGACACAGGTGCTGCATGGCTGTCGTCAGCTCGTGTCGTGAGA
TGTTGGGTTAAGTCCCGCAACGAGCGCAACCCTCGCCATTAGTTGCCATCATTTAG
TTGGGAACTCTAATGGGACTGCCGGTGCTAAGCCGGAGGAAGGTGGGGATGACGT
CAAGTCCTCATGGCCCTTACAGGGTGGGCTACACACGTGCTACAATGGCAACTACA
GAGGGTTAATCCTTAAAAGTTGTCTCAGTTCGGATTGTCCTCTGCAACTCGAGGGC
ATGAAGTTGGAATCGCTAGTAATCGCGGATCAGCATGCCGCGGTGAATACGTTCCC
GGGCCTTGTACACACCGCCCGTCACACCATGGGAGTTGGTTCTACCCGAAGGCGGT
GCGCTAACCAGCAATGGAGGCAGCCGACCACCGTAGGGTCAGCGACTGGGGTGAA
GTCGTAACAAGGTAGCCGTAGGGGAACCTGC
>Brevundimonas staleyi FWC43 (NR114710)
AGCGAACGCTGGCGGCAGGCCTAACACATGCAAGTCGAACGGACCCTTCGGGGTT
AGTGGCGGACGGGTGAGTAACACGTGGGAACGTGCCTTTAGGTTCGGAATAGCTC
CTGGAAACGGGTGGTAATGCCGAATGTGCCCTTCGGGGGAAAGATTTATCGCCTTT
AGAGCGGCCCGCGTCTGATTAGCTAGTTGGTGAGGTAAAGGCTCACCAAGGCGAC
GATCAGTAGCTGGTCTGAGAGGATGACCAGCCACATTGGGACTGAGACACGGCCC
AAACTCCTACGGGAGGCAGCAGTGGGGAATCTTGCGCAATGGGCGAAAGCCTGAC
GCAGCCATGCCGCGTGAATGATGAAGGTCTTAGGATTGTAAAATTCTTTCACCGGG
GACGATAATGACGGTACCCGGAGAAGAAGCCCCGGCTAACTTCGTGCCAGCAGCC
GCGGTAATACGAAGGGGGCTAGCGTTGCTCGGAATTACTGGGCGTAAAGGGAGCG
TAGGCGGACATTTAAGTCAGGGGTGAAATCCCAGAGCTCAACTCTGGAACTGCCTT
TGATACTGGGTGTCTTGAGTGTGATAGAGGTAAGTGGAACTCCGAGTGTAGAGGTG
AAATTCGTAGATATTCGGAAGAACACCAGTGGCGAAGGCGACTTACTGGATCATTA
CTGACGCTGAGGCTCGAAAGCGTGGGGAGCAAACAGGATTAGATACCCTGGTAGT
CCACGCCGTAAACGATGATTGCTAGTTGTTGGGGTGTTTACACCTCGGTGACGCAG
CTAACGCATTAAGCAATCCGCCTGGGGAGTACGGTCGCAAGATTAAAACTCAAAG
GAATTGACGGGGGCCCGCACAAGCGGTGGAGCATGTGGTTTAATTCGAAGCAACG
CGCAGAACCTTACCACCTTTTGACATGCCTGGACCGCCAGAGAGATCTGGCTTTCC
CTTCGGGGACTAGGACACAGGTGCTGCATGGCTGTCGTCAGCTCGTGTCGTGAGAT
GTTGGGTTAAGTCCCGCAACGAGCGCAACCCTCGCCATTAGTTGCCATCATTTAGT
TGGGAACTCTAATGGGACTGCCGGTGCTAAGCCGGAGGAAGGTGGGGATGACGTC
AAGTCCTCATGGCCCTTACAGGGTGGGCTACACACGTGCTACAATGGCAACTACAG
AGGGTTAATCCTTAAAAGTTGTCTCAGTTCGGATTGTCCTCTGCAACTCGAGGGCA
TGAAGTTGGAATCGCTAGTAATCGCGGATCAGCATGCCGCGGTGAATACGTTCCCG
GGCCTTGTACACACCGCCCGTCACACCATGGGAGTTGGTTCTACCCGAAGGCGATG

CGCTAACCGCAAGGAGGCAGTCGACCACGGTAGGGTCAGCGACTGGGGTGAAGTC
GTAACAAGGTAGCCGTAGGGGAACCTGC
>Brevundimonas bullata IAM 13153 (NR025831)
GAGCGAACGCTGGCGGCAGGCCTAACACATGCAAGTCGAACGGACCCTTCGGGGT
TAGTGGCGGACGGGTGAGTAACACGTGGGAACGTGCCTTTAGGTTCGGAATAGCTC
CTGGAAACGGGTGGTAATGCCGAATGTGCCCTTCGGGGGAAAGATTTATCGCCTTT
AGAGCGGCCCGCGTCTGATTAGCTAGTTGGTGAGGTAATGGCTCACCAAGGCGAC
GATCAGTAGCTGGTCTGAGAGGATGACCAGCCACATTGGGACTGAGACACGGCCC
AAACTCCTACGGGAGGCAGCAGTGGGGAATCTTGCGCAATGGGCGAAAGCCTGAC
GCAGCCATGCCGCGTGAATNATGAAGGTCTTAGGATTGTAAAATTCTTTCACCGGG
GACGATAATGACGGTACCCGGAGAAGAAGCCCCGGCTAACTTCGTGCCAGCAGCC
GCGGTAATACGAAGGGGGCTAGCGTTGCTCGGAATTACTGGGCGTAAAGGGCGCG
TAGGCGGACATTTAAGTCAGGGGTGAAATCCCAGAGCTCAACTCTGGAACTGCCTT
TGATACTGGGTGTCTTGAGTGTGAGAGAGGTATGTGGAACTCCGAGTGTAGAGGTG
AAATTCGTAGATATTCGGAAGAACACCAGTGGCGAAGGCGACATACTGGCTCATT
ACTGACGCTGAGGCGCGAAANNNTGGGGAGCAAACAGGATTAGATACCCTGGTAG
TCCACGCCGTAAACGATGATTGCTAGTTGTCGGGCTGCATGCAGTTCGGTGACGCA
GCTAACGCATTAAGCAATCCGCCTGGGGAGTACGGTCGCAAGATTAAAACTCAAA
GGAATTGACGGGGGCCCGCACAAGCGGTGGAGCATGTGGTTTAATTCGAAGCAAC
GCGCAGAACCTTACCACCTTTTGACATGCCTGGACCGCCAGAGAGATCTGGCTTTC
TCTTCGGAGACTAGGACACAGGTGCTGCATGGCTGTCGTCAGCTCGTGTCGTGAGA
TGTTGGGTTAAGTCCCGCAACGAGCGCAACCCTCGCCATTAGTTGCCATCATTTAG
TTGGGAACTCTAATGGGACTGCCGGTGCTAAGCCGGAGGAAGGTGGGGATGACGT
CAAGTCCTCATGGCCCTTACAGGGTGGGCTACACACGTGCTACAATGGCGACTACA
GAGGGTTAATCCTTAAAAGTCGTCTCAGTTCGGATTGTCCTCTGCAACTCGAGGGC
ATGAAGTTGGAATCGCTAGTAATCGCGGATCAGCATGCCGCGGTGAATACGTTCCC
GGGCCTTGTACACACCGCCCGTCACACCATGGGAGTTGGTTCTACCCGAAGGCGAT
GCGCTAACCCGCAAGGAGGCAGTCGACCACGGTAGGGTCAGCGACTGGGGTGAAG
TCGTAACAAGGTAGCCNTAGGGGAACC
>Brevundimonas lenta DS-18 (NR044186)
AGCGAACGCTGGCGGCAGGCCTAACACATGCAAGTCGAACGGACCTTTCGGGGTT
AGTGGCGGACGGGTGAGTAACACGTGGGAACGTGCCTTTAGGTTCGGAATAGCTC
CTGGAAACGGGTGGTAATGCCGAATGTGCCCTTCGGGGGAAAGATTTATCGCCTTT
AGAGCGGCCCGCGTCTGATTAGCTAGTTGGTGAGGTAAAGGCTCACCAAGGCTAC
GATCAGTAGCTGGTCTGAGAGGATGACCAGCCACATTGGGACTGAGACACGGCCC
AAACTCCTACGGGAGGCAGCAGTGGGGAATCTTGCGCAATGGGCGAAAGCCTGAC
GCAGCCATGCCGCGTGGATGATGAAGGTCTTAGGATTGTAAAATCCTTTCACCGGT
GAAGATAATGACTGTAGCCGGAGAAGAAGCCCCGGCTAACTTCGTGCCAGCAGCC
GCGGTAATACGAAGGGGGCTAGCGTTGCTCGGAATTACTGGGCGTAAAGGGAGCG
TAGGCGGACATTTAAGTCAGGGGTGAAATCCCAGAGCTCAACTCTGGAACTGCCTT
TGATACTGGGTGTCTTGAGTGTGAGAGAGGTATGTGGAACTCCGAGTGTAGAGGTG
AAATTCGTAGATATTCGGAAGAACACCAGTGGCGAAGGCGACATACTGGCTCATT
ACTGACGCTGAGGCTCGAAAGCGTGGGGAGCAAACAGGATTAGATACCCTGGTAG
TCCACGCCGTAAACGATGATTGCTAGTTGTCGGGAAGCTTGCTTCTCGGTGACGCA
GCTAACGCATTAAGCAATCCGCCTGGGGAGTACGGTCGCAAGATTAAAACTCAAA
GGAATTGACGGGGGCCCGCACAAGCGGTGGAGCATGTGGTTTAATTCGAAGCAAC
GCGCAGAACCTTACCACCTTTTGACATGCCCGGACCGCCACAGAGATGTGGCTTTC
CCTTCGGGGACTGGGACACAGGTGCTGCATGGCTGTCGTCAGCTCGTGTCGTGAGA
TGTTGGGTTAAGTCCCGCAACGAGCGCAACCCTCGCCATTAGTTGCCATCATTCAG
TTGGGAACTCTAATGGGACTGCCGGTGCTAAGCCGGAGGAAGGTGGGGATGACGT
CAAGTCCTCATGGCCCTTACAGGGTGGGCTACACACGTGCTACAATGGCGACTACA

GAGGGTTAATCCTTAAAAGTCGTCTCAGTTCGGATTGTCCTCTGCAACTCGAGGGC
ATGAAGTTGGAATCGCTAGTAATCGCGGATCAGCATGCCGCGGTGAATACGTTCCC
GGGCCTTGTACACACCGCCCGTCACACCATGGGAGTTGGTTCTACCCGAAGGCGGT
GCGCTAACCAGCAATGGAGGCAGCCGACCACGGTAGGGTCAGCGACTGGGGTGAA
GTCGTAACAAGGTAGCCGTAGGGGAACCTGC
>Acinetobacter ursingii DSM 16037(AIEA01000080)
AGAGTTTGATCATGGCTCAGATTGAACGCTGGCGGCAGGCTTAACACATGCAAGTC
GAGCGGAGAGAGGTAGCTTGCTACTGATCTTAGCGGCGGACGGGTGAGTAATACT
TAGGAATCTGGCTATTAGTGGGGGACAACATTTCGAAAGGGATGCTAATACCGCAT
ACGTCCTACGGGAGAAAGCAGGGGATCTTCGGACCTTGCGCTAATAGATGAGCCT
AAGTCGGATTAGCTAGTTGGTGGGGTAAAGGCCTACCAAGGCGACGATCTGTAGC
GGGTCTGAGAGGATGATCCGCCACACTGGGACTGAGACACGGCCCAGACTCCTAC
GGGAGGCAGCAGTGGGGAATATTGGACAATGGGGGGAACCCTGATCCAGCCATGC
CGCGTGTGTGAAGAAGGCCTTATGGTTGTAAAGCACTTTAAGCGAGGAGGAGGGT
ACTGGTATTAATACTACCAGGTACTGGACGTTACTCGCAGAATAAGCACCGGCTAA
CTCTGTGCCAGCAGCCGCGGTAATACAGAGGGTGCGAGCGTTAATCGGATTTACTG
GGCGTAAAGCGTGCGTAGGCGGCTAATTGAGTCGGATGTGAAATCCCCGAGCTTA
ACTTGGGAATTGCATTCGATACTGGTTAGCTAGAGTGTGGGAGAGGATGGTAGAAT
TCCAGGTGTAGCGGTGAAATGCGTAGAGATCTGGAGGAATACCGATGGCGAAGGC
AGCCATCTGGCCTAACACTGACGCTGAGGTACGAAAGCATGGGGAGCAAACAGGA
TTAGATACCCTGGTAGTCCATGCCGTAAACGATGTCTACTAGCCGTTGGTCTCTTTG
AGGGATTAGTGGCGCAGCTAACGCGATAAGTAGACCGCCTGGGGAGTACGGTCGC
AAGACTAAAACTCAAATGAATTGACGGGGGCCCGCACAAGCGGTGGAGCATGTGG
TTTAATTCGATGCAACGCGAAGAACCTTACCTGGTCTTGACATAGTAAGAACTTTC
CAGAGATGGATTGGTGCCTTCGGGAACTTACATACAGGTGCTGCATGGCTGTCGTC
AGCTCGTGTCGTGAGATGTTGGGTTAAGTCCCGCAACGAGCGCAACCCTTTTCCTT
ATTTGCCAGCACTTTGGGTGGGAACTTTAAGGATACTGCCAGTGACAAACTGGAGG
AAGGCGGGGACGACGTCAAGTCATCATGGCCCTTACGACCAGGGCTACACACGTG
CTACAATGGTCGGTACAAAGGGTTGCTACCTAGCGATAGGATGCTAATCTCAAAAA
GCCGATCGTAGTCCGGATTGGAGTCTGCAACTCGACTCCATGAAGTCGGAATCGCT
AGTAATCGCGGATCAGAATGCCGCGGTGAATACGTTCCCGGGCCTTGTACACACCG
CCCGTCACACCATGGGAGTTTGTTGCACCAGAAGTGGATAGTCTAACTGCAAAGAG
GACGTTCACCACGGTGTGGCCGATGACTGGGGTGAAGTCGTAACAAGGTA
>Acinetobacter seifertii LUH 1472 (NR134684)
ATTGAACGCTGGCGGCAGGCTTAACACATGCAAGTCGAGCGGAGAGAGGTAGCTT
GCTACTGATCTTAGCGGCGGACGGGTGAGTAATGCTTAGGAATCTGCCTATTAGTG
GGGGACAACATCTCGAAAGGGATGCTAATACCGCATACGTCCTACGGGAGAAAGC
AGGGGATCTTCGGACCTTGCGCTAATAGATGAGCCTAAGTCGGATTAGCTAGTTGG
TGGGGTAAAGGCCTACCAAGGCGACGATCTGTAGCGGGTCTGAGAGGATGATCCG
CCACACTGGGACTGAGACACGGCCCAGACTCCTACGGGAGGCAGCAGTGGGGAAT
ATTGGACAATGGGGGGAACCCTGATCCAGCCATGCCGCGTGTGTGAAGAAGGCCT
TATGGTTGTAAAGCACTTTAAGCGAGGAGGAGGCTACTTTAGTTAATACCTAGAGA
TAGTGGACGTTACTCGCAGAATAAGCACCGGCTAACTCTGTGCCAGCAGCCGCGGT
AATACAGAGGGTGCAAGCGTTAATCGGATTTACTGGGCGTAAAGCGCGCGTAGGC
GGCTAATTAAGTCAAATGTGAAATCCCCGAGCTTAACTTGGGAATTGCATTCGATA
CTGGTTAGCTAGAGTGTGGGAGAGGATGGTAGAATTCCAGGTGTAGCGGTGAAAT
GCGTAGAGATCTGGAGGAATACCGATGGCGAAGGCAGCCATCTGGCCTAACACTG
ACGCTGAGGTGCGAAAGCATGGGGAGCAAACAGGATTAGATACCCTGGTAGTCCA
TGCCGTAAACGATGTCTACTAGCCGTTGGGGCCTTTGAGGCTTTAGTGGCGCAGCT
AACGCGATAAGTAGACCGCCTGGGGAGTACGGTCGCAAGACTAAAACTCAAATGA
ATTGACGGGGGCCCGCACAAGCGGTGGAGCATGTGGTTTAATTCGATGCAACGCG

AAGAACCTTACCTGGCCTTGACATAGTAAGAACTTTCCAGAGATGGATTGGTGCCT
TCGGGAACTTACATACAGGTGCTGCATGGCTGTCGTCAGCTCGTGTCGTGAGATGT
TGGGTTAAGTCCCGCAACGAGCGCAACCCTTTTCCTTATTTGCCAGCGAGTAATGT
CGGGAACTTTAAGGATACTGCCAGTGACAAACTGGAGGAAGGCGGGGACGACGTC
AAGTCATCATGGCCCTTACGGCCAGGGCTACACACGTGCTACAATGGTCGGTACAA
AGGGTTGCTACACAGCGATGTGATGCTAATCTCAAAAAGCCGATCGTAGTCCGGAT
TGGAGTCTGCAACTCGACTCCATGAAGTCGGAATCGCTAGTAATCGCGGATCAGAA
TGCCGCGGTGAATACGTTCCCGGGCCTTGTACACACCGCCCGTCACACCATGGGAG
TTTGTTGCACCAGAAGTAGCTAGCCTAACTGCAAAGAGGGCGGTTACCACGGTGTG
GCCGATGACTGGGGTG
>Pseudomonas parafulva NBRC 16636 (NR113856)
ATTGAACGCTGGCGGCAGGCCTAACACATGCAAGTCGAGCGGATGACGGGAGCTT
GCTCCTNGATTCAGCGGCGGACGGGTGAGTAATGCCTAGGAATCTGCCTGGTAGTG
GGGGACAACGTTTCGAAAGGAACGCTAATACCGCATACGTCCTACGGGAGAAAGC
AGGGGACCTTCGGGCCTTGCGCTATCAGATGAGCCTAGGTCGGATTAGCTTGTTGG
TGAGGTAATGGCTCACCAAGGCGACGATCCGTAACTGGTCTGAGAGGATGATCAG
TCACACTGGAACTGAGACACGGTCCAGACTCCTACGGGAGGCAGCAGTGGGGAAT
ATTGGACAATGGGCGAAAGCCTGATCCAGCCATGCCGCGTGTGTGAAGAAGGTCTT
CGGATTGTAAAGCACTTTAAGTTGGGAGGAAGGGTTGTAGATTAATACTCTGCAAT
TTTGACGTTACCGACAGAATAAGCACCGGCTAACTCTGTGCCAGCAGCCGCGGTAA
TACAGAGGGTGCAAGCGTTAATCGGAATTACTGGGCGTAAAGCGCGCGTAGGTGG
TTTGTTAAGTTGGATGTGAAAGCCCCGGGCTCAACCTGGGAACTGCATCCAAAACT
GGCAAGCTAGAGTACGGTAGAGGGTGGTGGAATTTCCTGTGTAGCGGTGAAATGC
GTAGATATAGGAAGGAACACCAGTGGCGAAGGCGACCACCTGGACTGATACTGAC
ACTGAGGTGCGAAAGCGTGGGGAGCAAACAGGATTAGATACCCTGGTAGTCCACG
CCGTAAACGATGTCAACTAGCCGTTGGAATCCTTGAGATTTTAGTGGCGCAGCTAA
CGCATTAAGTTGACCGCCTGGGGAGTACGGCCGCAAGGTTAAAACTCAAATGAATT
GACGGGGGCCCGCACAAGCGGTGGAGCATGTGGTTTAATTCGAAGCAACGCGAAG
AACCTTACCAGGCCTTGACATGCAGAGAACTTTCCAGAGATGGATTGGTGCCTTCG
GGAACTCTGACACAGGTGCTGCATGGCTGTCGTCAGCTCGTGTCGTGAGATGTTGG
GTTAAGTCCCGTAACGAGCGCAACCCTTGTCCTTAGTTACCAGCACGTTATGGTGG
GCACTCTAAGGAGACTGCCGGTGACAAACCGGAGGAAGGTGGGGATGACGTCAAG
TCATCATGGCCCTTACGGCCTGGGCTACACACGTGCTACAATGGTCGGTACAGAGG
GTTGCCAAGCCGCGAGGTGGAGCTAATCTCACAAAACCGATCGTAGTCCGGATCGC
AGTCTGCAACTCGACTGCGTGAAGTCGGAATCGCTAGTAATCGCGAATCAGAATGT
CGCGGTGAATACGTTCCCGGGCCTTGTACACACCGCCCGTCACACCATGGGAGTGG
GTTGCACCAGAAGTAGCTAGTCTAACCTTCGGGAGGACGGTTACCACGGTGTGATT
CATGACTGGGGTGAAG
>Pseudomonas cremoricolorata NBRC 16634 (NR113855)
ATTGAACGCTGGCGGCAGGCCTAACACATGCAAGTCGAGCGGATGACAGGAGCTT
GCTCCTGGATTCAGCGGCGGACGGGTGAGTAATGCCTAGGAATCTGCCTGGTAGTG
GGGGACAACGTTTCGAAAGGAACGCTAATACCGCATACGTCCTACGGGAGAAAGC
AGGGGACCTTCGGGCCTTGCGCTATCAGATGAGCCTAGGTCGGATTAGCTTGTTGG
TGAGGTAATGGCTCACCAAGGCGACGATCCGTAACTGGTCTGAGAGGATGATCAG
TCACACTGGAACTGAGACACGGTCCAGACTCCTACGGGAGGCAGCAGTGGGGAAT
ATTGGACAATGGGCGAAAGCCTGATCCAGCCATGCCGCGTGTGTGAAGAAGGTCTT
CGGATTGTAAAGCACTTTAAGTTGGGAGGAAGGGTTGTAGATTAATACTCTGCAAT
TTTGACGTTACCGACAGAATAAGCACCGGCTAACTCTGTGCCAGCAGCCGCGGTAA
TACAGAGGGTGCAAGCGTTAATCGGAATTACTGGGCGTAAAGCGCGCGTAGGTGG
TTTGTTAAGTTGGATGTGAAAGCCCCGGGCTCAACCTGGGAACTGCATCCAAAACT
GGCAAGCTAGAGTACGGTAGAGGGTGGTGGAATTTCCTGTGTAGCGGTGAAATGC

GTAGATATAGGAAGGAACACCAGTGGCGAAGGCGACCACCTGGACTGATACTGAC
ACTGAGGTGCGAAAGCGTGGGGAGCAAACAGGATTAGATACCCTGGTAGTCCACG
CCGTAAACGATGTCAACTAGCCGTTGGAATCCTTGAGATTTTAGTGGCGCAGCTAA
CGCATTAAGTTGACCGCCTGGGGAGTACGGCCGCAAGGTTAAAACTCAAATGAATT
GACGGGGGCCCGCACAAGCGGTGGAGCATGTGGTTTAATTCGAAGCAACGCGAAG
AACCTTACCAGGCCTTGACATGCAGAGAACTTTCCAGAGATGGATTGGTGCCTTCG
GGAACTCTGACACAGGTGCTGCATGGCTGTCGTCAGCTCGTGTCGTGAGATGTTGG
GTTAAGTCCCGTAACGAGCGCAACCCTTGTCCTTAGTTACCAGCACGTTATGGTGG
GCACTCTAAGGAGACTGCCGGTGACAAACCGGAGGAAGGTGGGGATGACGTCAAG
TCATCATGGCCCTTACGGCCTGGGCTACACACGTGCTACAATGGTCGGTACAGAGG
GTTGCCAAGCCGCGAGGTGGAGCTAATCTCACAAAACCGATCGTAGTCCGGATCGC
AGTCTGCAACTCGACTGCGTGAAGTCGGAATCGCTAGTAATCGCGAATCAGAATGT
CGCGGTGAATACGTTCCCGGGCCTTGTACACACCGCCCGTCACACCATGGGAGTGG
GTTGCACCAGAAGTAGCTAGTCTAACCTTCGGGNGGACGGTTACCACGGTGTGATT
CATGACTGGGGTGAAG
>Pseudomonas parafulva AJ 2129 (NR040859)
ATCCTGGCTCAGATTGAACGCTGGCGGCAGGCCTAACACATGCAAGTCGAGCGGA
TGACGGGAGCTTGCTCCTTGATTCAGCGGCGGACGGGTGAGTAATGCCTAGGAATC
TGCCTGGTAGTGGGGGACAACGTTTCGAAAGGAACGCTAATACCGCATACGTCCTA
CGGGAGAAAGCAGGGGACCTTCGGGCCTTGCGCTATCAGATGAGCCTAGGTCGGA
TTAGCTTGTTGGTGAGGTAATGGCTCACCAAGGCGACGATCCGTAACTGGTCTGAG
AGGATGATCAGTCACACTGGAACTGAGACACGGTCCAGACTCCTACGGGAGGCAG
CAGTGGGGAATATTGGACAATGGGCGAAAGCCTGATCCAGCCATGCCGCGTGTGT
GAAGAAGGTCTTCGGATTGTAAAGCACTTTAAGTTGGGAGGAAGGGTTGTAGATTA
ATACTCTGCAATTTTGACGTTACCGACAGAATAAGCACCGGCTAACTCTGTGCCAG
CAGCCGCGGTAATACAGAGGGTGCAAGCGTTAATCGGAATTACTGGGCGTAAAGC
GCGCGTAGGTGGTTTGTTAAGTTGGATGTGAAAGCCCCGGGCTCAACCTGGGAACT
GCATCCAAAACTGGCAAGCTAGAGTACGGTAGAGGGTGGTGGAATTTCCTGTGTA
GCGGTGAAATGCGTAGATATAGGAAGGAACACCAGTGGCGAAGGCGACCACCTGG
ACTGATACTGACACTGAGGTGCGAAAGCGTGGGGAGCAAACAGGATTAGATACCC
TGGTAGTCCACGCCGTAAACGATGTCAACTAGCCGTTGGAATCCTTGAGATTTTAG
TGGCGCAGCTAACGCATTAAGTTGACCGCCTGGGGAGTACGGCCGCAAGGTTAAA
ACTCAAATGAATTGACGGGGGCCCGCACAAGCGGTGGAGCATGTGGTTTAATTCG
AAGCAACGCGAAGAACCTTACCAGGCCTTGACATGCAGAGAACTTTCCAGAGATG
GATTGGTGCCTTCGGGAACTCTGACACAGGTGCTGCATGGCTGTCGTCAGCTCGTG
TCGTGAGATGTTGGGTTAAGTCCCGTAACGAGCGCAACCCTTGTCCTTAGTTACCA
GCACGTTATGGTGGGCACTCTAAGGAGACTGCCGGTGACAAACCGGAGGAAGGTG
GGGATGACGTCAAGTCATCATGGCCCTTACGGCCTGGGCTACACACGTGCTACAAT
GGTCGGTACAGAGGGTTGCCAAGCCGCGAGGTGGAGCTAATCTCACAAAACCGAT
CGTAGTCCGGATCGCAGTCTGCAACTCGACTGCGTGAAGTCGGAATCGCTAGTAAT
CGCGAATCAGAATGTCGCGGTGAATACGTTCCCGGGCCTTGTACACACCGCCCGTC
ACACCATGGGAGTGGGTTGCACCAGAAGTAGCTAGTCTAACCTTCGGGAGGACGG
TTACCACGGTGTGATTCATGACTGGGGTGAAGTCGTAACAAG
>Pseudomonas fulva IAM1529 (NR115610)
AGTTTGATCCTGGCTCAGATTGAACGCTGGCGGCAGGCCTAACACATGCAAGTCGA
GCGGATGACAGGAGCTTGCTCCTGGATTCAGCGGCGGACGGGTGAGTAATGCCTA
GGAATCTGCCTGGTAGTGGGGGACAACGTTTCGAAAGGAACGCTAATACCGCATA
CGTCCTACGGGAGAAAGCAGGGGACCTTCGGGCCTTGCGCTATCAGATGAGCCTA
GGTCGGATTAGCTTGTTGGTGAGGTAATGGCTCACCAAGGCGACGACCTGTAACTG
GTCTGAGAGGATGATCAGTCACACTGGAACTGAGACACGGTCCAGACTCCTACGG
GAGGCAGCAGTGGGGAATATTGGACAATGGGCGAAAGCCTGATCCAGCCATGCCG

CGTGTGTGAAGAAGGTCTTCGGATTGTAAAGCACTTTAAGTTGGGAGGAAGGGTTG
TAGATTAATACTCTGCAATTTTGACGTTACCGACAGAATAAGCACCGGCTAACTCT
GTGCCAGCAGCCGCGGTAATACAGAGGGTGCAAGCGTTAATCGGAATTACTGGGC
GTAAAGCGCGCGTAGGTGGTTTGTTAAGTTGGATGTGAAAGCCCCGGGCTCAACCT
GGGAACTGCATCCAAAACTGGCAAGCTAGAGTACGGTAGAGGGTGGTGGAATTTC
CTGTGTAGCGGTGAAATGCGTAGATATAGGAAGGAACACCAGTGGCGAAGGCGAC
CACCTGGACTGATACTGACACTGAGGTGCGAAAGCGTGGGGAGCAAACAGGATTA
GATACCCTGGTAGTCCACGCCGTAAACGATGTCAACTAGCCGTTGGAATCCTTGAG
ATTTTAGTGGCGCAGCTAACGCATTAAGTTGACCGCCTGGGGAGTACGGCCGCAAG
GTTAAAACTCAAATGAATTGACGGGGGCCCGCACAAGCGGTGGAGCATGTGGTTT
AATTCGAAGCAACGNGAAGAACCTTACCAGGCCTTNACATGCAGAGAACTTTTCA
GNGATGGTATTGTGCCTTCGGGAACTCTACACAGGTGCTGCATGNCTGTCGTCAGC
TCGTGTCGTGAGATGTTGGGTTAAGTCCCGTAACGAGCGCAACCCTTGTCCTTAGTT
ACCAGCACATTATGGTGGGCACTCTAAGGAGACTGCCGGTGACAAACCGGAGGAA
GGTGGGGATGACGTCAAGTCATCATGGCCCTTACGGCCTGGGCTACACACGTGCTA
CAATGGTCGGTACAGAGGGTTGCCAAGCCGCGAGGTGGAGCTAATCTCACAAAAC
CGATCGTAGTCCGGATCGCAGTCTGCAACTCGACTGCGTGAAGTCGGAATCGCTAG
TAATCGCGAATCAGAATGTCGCGGTGAATACGTTCCCGGGCCTTGTACACACCGCC
CGTCACACCATGGGAGTGGGTTGCACCAGAAGTAGCTAGTCTAACCTTCGGGGGG
ACGGTTACCACGGTGTGATTCATGACTGGGGTGAAGTCGTAACAAGGTAGCCGTAG
GGGAACCTGCGGCTGGATCACCTCCTT
>Pseudomonas reinekei MT1 (NR042541)
ATTGAACGCTGGCGGCAGGCCTAACACATGCAAGTCGAGCGGATGACAGGAGCTT
GCTCCTGAATTCAGCGGCGGACGGGTGAGTAATGCCTAGGAATCTGCCTGRTAGTG
GGGGACAACGTTTCGAAAGGAACGCTAATACCGCATACGTCCTACGGGAGAAAGC
AGGGGACCTTCGGGCCTTGCGCTATCAGATGAGCCTAGGTCGGATTAGCTAGTTGG
TGAGGTAATGGCTCACCAAGGCGACGATCCGTAACTGGTCTGAGAGGATGATCAG
TCACACTGGAACTGAGACACGGTCCAGACTCCTACGGGAGGCAGCAGTGGGGAAT
ATTGGACAATGGGCGAAAGCCTGATCCAGCCATGCCGCGTGTGTGAAGAAGGTCTT
CGGATTGTAAAGCACTTTAAGTTGGGAGGAAGGGTTGTAGATTAATACTCTGCAAT
TTTGACGTTACCGACAGAATAAGCACCGGCTAACTCTGTGCCAGCAGCCGCGGTAA
TACAGAGGGTGCAAGCGTTAATCGGAATTACTGGGCGTAAAGCGCGCGTAGGTGG
TTTGTTAAGTTGGATGTGAAATCCCCGGGCTCAACCTGGGAACTGCATTCAAAACT
GACAAGCTAGAGTATGGTAGAGGGTGGTGGAATTTCCTGTGTAGCGGTGAAATGC
GTAGATATAGGAAGGAACACCAGTGGCGAAGGCGACCACCTGGACTGATACTGAC
ACTGAGGTGCGAAAGCGTGGGGAGCAAACAGGATTAGATACCCTGGTAGTCCACG
CCGTAAACGATGTCAACTAGCCGTTGGGAGCCTTGAGCTCTTAGTGGCGCAGCTAA
CGCATTAAGTTGACCGCCTGGGGAGTACGGCCGCAAGGTTAAAACTCAAATGAATT
GACGGGGGCCCGCACAAGCGGTGGAGCATGTGGTTTAATTCGAAGCAACGCGAAG
AACCTTACCAGGCCTTGACATCCAATGAACTTTCCAGAGATGGATTGGTGCCTTCG
GGAACATTGAGACAGGTGCTGCATGGCTGTCGTCAGCTCGTGTCGTGAGATGTTGG
GTTAAGTCCCGTAACGAGCGCAACCCTTGTCCTTAGTTACCAGCACGTTATGGTGG
GCACTCTAAGGAGACTGCCGGTGACAAACCGGAGGAAGGTGGGGATGACGTCAAG
TCATCATGGCCCTTACGGCCTGGGCTACACACGTGCTACAATGGTCGGTACAGAGG
GTTGCCAAGCCGCGAGGTGGAGCTAATCCCATAAAACCGATCGTAGTCCGGATCGC
AGTCTGCAACTCGACTGCGTGAAGTCGGAATCGCTAGTAATCGCGAATCAGAATGT
CGCGGTGAATACGTTCCCGGGCCTTGTACACACCGCCCGTCACACCATGGGAGTGG
GTTGCACCAGAAGTAGCTAGTCTAACCTTCGGGAGGACGGTTACCACGGTGTGATT
CATGACTGGGGTGAAGTCGTAACAAGGTAGCCGTAGGGGAACCTGC
>Pseudomonas alcaligenes ATCC 14909 (NR114472)

TGGCCTCAGATTGAACGCTGGCGGCAGGCCTAACACATGCAAGTCGAGCGGATGA
GTGGAGCTTGCTCCATGATTCAGCGGCGGACGGGTGAGTAATGCCTAGGAATCTGC
CTGGTAGTGGGGGACAACGTTTCGAAAGGAACGCTAATACCGCATACGTCCTACG
GGAGAAAGCAGGGGACCTTCGGGCCTTGCGCTATCAGATGAGCCTAGGTCGGATT
AGCTAGTAGGTGAGGTAATGGCTCACCTAGGCGACGATCCGTAACTGGTCTGAGA
GGATGATCAGTCACACTGGAACTGAGACACGGTCCAGACTCCTACGGGAGGCAGC
AGTGGGGAATATTGGACAATGGGCGAAAGCCTGATCCAGCCATGCCGCGTGTGTG
AAGAAGGTCTTCGGATTGTAAAGCACTTTAAGTTGGGAGGAAGGGCAGTAAGTTA
ATACCTTGCTGTTTTGACGTTACCAACAGAATAAGCACCGGCTAACTTCGTGCCAG
CAGCCGCGGTAATACGAAGGGTGCAAGCGTTAATCGGAATTACTGGGCGTAAAGC
GCGCGTAGGTGGTTCAGCAAGTTGGAGGTGAAATCCCCGGGCTCAACCTGGGAAC
TGCCTCCAAAACTACTGAGCTAGAGTACGGTAGAGGGTAGTGGAATTTCCTGTGTA
GCGGTGAAATGCGTAGATATAGGAAGGAACACCAGTGGCGAAGGCGACTACCTGG
ACTGATACTGACACTGAGGTGCGAAAGCGTGGGGASCAAACAGGATTAGATACCC
TGGTAGTCCACGCCGTAAACGATGTCGACTAGCCGTTGGGATCCTTGAGATCTTAG
TGGCGCAGCTAACGCATTAAGTCGACCGCCTGGGGAGTACGGCCGCAAGGTTAAA
ACTCAAATGAATTGACGGGGGCCCGCACAAGCGGTGGAGCATGTGGTTTAATTCG
AAGCAACGCGAAGAACCTTACCTGGCCTTGACATGCTGAGAACTTTCCAGAGATGG
ATTGGTGCCTTCGGGAACTCAGACACAGGTGCTGCATGGCTGTCGTCAGCTCGTGT
CGTGAGATGTTGGGTTAAGTCCCGTAACGAGCGCAACCCTTGTCCTTAGTTACCAG
CACGTTATGGTGGGCACTCTAAGGAGACTGCCGGTGACAAACCGGAGGAAGGTGG
GGATGACGTCAAGTCATCATGGCCCTTACGGCCAGGGCTACACACGTGCTACAATG
GTCGGTACAAAGGGTTGCCAAGCCGCGAGGTGGAGCTAATCCCATAAAACCGATC
GTAGTCCGGATCGCAGTCTGCAACTCGACTGCGTGAAGTCGGAATCGCTAGTAATC
GTGAATCAGAATGTCACGGTGAATACGTTCCCGGGCCTTGTACACACCGCCCGTCA
CACCATGGGAGTGGGTTGCTCCAGAAGTAGCTAGTCTAACCGCAAGGGGGACGGT
TACCACGGAGTGATTCATG
>Chryseobacterium haifense H38 (NR044167)
ATGGCTCAGGATGAACGCTAGCGGGAGGCCTAACACATGCAAGCCGAGCGGTAGA
GTTNCTTCGGNAACTTGAGAGCGGCGCACGGGTGCGTAACACGTGTGCAACCTACC
TTTATCTGGGGGATAGCCTTTCGAAAGGAAGATTAATACCCCATAATATAATGATT
GGCATCAATTATTATTGAAAACTCCGGTGGATAGAGATGGGCACGCGCAAGATTA
GATAGTTGGTGAGGTAACGGCTCACCAAGTCAATGATCTTTAGGGGTCCTGAGAGG
GAGATCCCCCACACTGGTACTGAGACACGGACCAGACTCCTACGGGAGGCAGCAG
TGAGGAATATTGGACAATGGGTGAGAGCCTGATCCAGCCATCCCGCGTGAAGGAT
GACGGTCCTATGGATTGTAAACTTCTTTTGTACAGGGATAAACCTACTCTCGTGAG
GGTAGCTGAAGGTACTGTACGAATAAGCACCGGCTAACTCCGTGCCAGCAGCCGC
GGTAATACGGAGGGTGCAAGCGTTATCCGGATTTATTGGGTTTAAAGGGTCCGTAG
GCGGACTTGTAAGTCAGTGGTGAAATCTCATAGCTTAACTATGAAACTGCCATTGA
TACTGCAGGTCTTGAGTAAATTTGAAGTGGCTGGAATAAGTAGTGTAGCGGTGAAA
TGCATAGATATTACTTAGAACACCAATTGCGAAGGCAGGTCACTAAGATTTAACTG
ACGCTGATGGACGAAAGCGTGGGGAGCGAACAGGATTAGATACCCTGGTAGTCCA
CGCCGTAAACGATGCTAACTCGTTTTTGGATTTTCGGATTCAGAGACCAAGCGAAA
GTGATAAGTTAGCCACCTGGGGAGTACGTCCGCAAGGATGAAACTCAAAGGAATT
GACGGGGGCCCGCACAAGCGGTGGATTATGTGGTTTAATTCGATGATACGCGAGG
AACCTTACCAAGACTTAAATGGGAATTGACAGGTCTAGAAATAGACCCTCCTTCGG
GCAATTTCCAAGGTGCTGCATGGTTGTCGTCAGCTCGTGCCGTGAGGTGTTAGGTT
AAGTCCTGCAACGAGCGCAACCCCTGTCACTAGTTGCTAACATTTAGTTGAGGACT
CTAGTGAGACTGCCTACGCAAGTAGAGAGGAAGGTGGGGATGACGTCAAATCATC
ACGGCCCTTACGTCTTGGGCCACACACGTAATACAATGGCCGGTACAGAGGGCAG
CTACACAGCGATGTGATGCAAATCTCGAAAGCCGGTCTCAGTTCGGATTGGAGTCT

GCAACTCGACTCTATGAAGCTGGAATCGCTAGTAATCGCGCATCAGCCATGGCGCG
GTGAATACGTTCCCGGGCCTTGTACACACCGCCCGTCAAGCCATGGAAGTCTGGGG
TACCTGAAGTCGGTGACCGTAAAAGGAGCTGCCTAGGGTAAAACAGGTAACTAGG
GCTAAGTCGTAACAAGCTAACCGTAAGCTTTCACA
>Chryseobacterium lactis KC1864 (NR126256)
GATGAACGCTAGCGGGAGGCCTAACACATGCAAGCCGAGCGGTAGAGATTCTTCG
GAATCTTGAGAGCGGCGTACGGGTGCGGAACACGTGTGCAACCTGCCTTTATCTGG
GGGATAGCCTTTCGAAAGGAAGATTAATACCCCATAATATATTGAATGGCATCATT
TGATATTGAAAACTCCGGTGGATAGAGATGGGCACGCGCAAGATTAGATAGTTGG
TGAGGTAACGGCTCACCAAGTCTACGATCTTTAGGGGGCCTGAGAGGGTGATCCCC
CACACTGGTACTGAGACACGGACCAGACTCCTACGGGAGGCAGCAGTGAGGAATA
TTGGACAATGGGTGAGAGCCTGATCCAGCCATCCCGCGTGAAGGACGACGGCCCT
ATGGGTTGTAAACTTCTTTTGTATAGGGATAAACCTACTCTCGTGAGAGTAGCTGA
AGGTACTATACGAATAAGCACCGGCTAACTCCGTGCCAGCAGCCGCGGTAATACG
GAGGGTGCAAGCGTTATCCGGATTTATTGGGTTTAAAGGGTCCGTAGGCGGATCTG
TAAGTCAGTGGTGAAATCTCACAGCTTAACTGTGAAACTGCCATTGATACTGCAGG
TCTTGAGTGTTGTTGAAGTAGCTGGAATAAGTAGTGTAGCGGTGAAATGCATAGAT
ATTACTTAGAACACCAATTGCGAAGGCAGGTTACTAAGCAACAACTGACGCTGATG
GACGAAAGCGTGGGGAGCGAACAGGATTAGATACCCTGGTAGTCCACGCCGTAAA
CGATGCTAACTCGTTTTTGGGCTTTCGGGTTCAGAGACTAAGCGAAAGTGATAAGT
TAGCCACCTGGGGAGTACGTTCGCAAGAATGAAACTCAAAGGAATTGACGGGGGC
CCGCACAAGCGGTGGATTATGTGGTTTAATTCGATGATACGCGAGGAACCTTACCA
AGGCTTAAATGGGAAATGACAGGTTTAGAAATAGACTTTTCTTCGGACATTTTTCA
AGGTGCTGCATGGTTGTCGTCAGCTCGTGCCGTGAGGTGTTAGGTTAAGTCCTGCA
ACGAGCGCAACCCCTGTCACTAGTTGCCATCATTAAGTTGGGGACTCTAGTGAGAC
TGCCTACGCAAGTAGAGAGGAAGGTGGGGATGACGTCAAATCATCACGGCCCTTA
CGCCTTGGGCCACACACGTAATACAATGGCCAGTACAGAGGGCAGCTACACAGCG
ATGTGATGCAAATCTCGAAAGCTGGTCTCAGTTCGGATTGGAGTCTGCAACTCGAC
TCTATGAAGCTGGAATCGCTAGTAATCGCGCATCAGCCATGGCGCGGTGAATACGT
TCCCGGGCCTTGTACACACCGCCCGTCAAGCCATGGAAGTCTGGGGTACCTGAAGT
CGGTGACCGTAATAGGAGCTGCCTAGGGTAAAACAGGTAACTAGGGCT
>Acinetobacter pittii CIP 70.29 (NR116774)
TAGAGTTTGATCCTGGCTCAGATTGAACGCTGGCGGCAGGCTTAACACATGCAAGT
CGAGCGGAGAGAGGTAGCTTGCTACTGATCTTAGCGGCGGACGGGTGAGTAATGC
TTAGGAATCTGCCTATTAGTGGGGGACAACATTTCGAAAGGAATGCTAATACCGCA
TACGTCCTACGGGAGAAAGCAGGGGATCTTCGGACCTTGCGCTAATAGATGAGCCT
AAGTCGGATTAGCTAGTTGGTGGGGTAAAGGCCTACCAAGGCGACGATCTGTAGC
GGGTCTGAGAGGATGATCCGCCACACTGGGACTGAGACACGGCCCAGACTCCTAC
GGGAGGCAGCAGTGGGGAATATTGGACAATGGGCGCAAGCCTGATCCAGCCATGC
CGCGTGTGTGAAGAAGGCCTTATGGTTGTAAAGCACTTTAAGCGAGGAGGAGGCT
ACTTTAGTTAATACCTAGAGATAGTGGACGTTACTCGCAGAATAAGCACCGGCTAA
CTCTGTGCCAGCAGCCGCGGTAATACAGAGGGTGCAAGCGTTAATCGGATTTACTG
GGCGTAAAGCGCGCGTAGGCGGCTAATTAAGTCAAATGTGAAATCCCCGAGCTTA
ACTTGGGAATTGCATTCGATACTGGTTAGCTAGAGTGTGGGAGAGGATGGTAGAAT
TCCAGGTGTAGCGGTGAAATGCGTAGAGATCTGGAGGAATACCGATGGCGAAGGC
AGCCATCTGGCCTAACACTGACGCTGAGGTGCGAAAGCATGGGGAGCAAACAGGA
TTAGATACCCTGGTAGTCCATGCCGTAAACGATGTCTACTAGCCGTTGGGGCCTTT
GAGGCTTTAGTGGCGCAGCTAACGCGATAAGTAGACCGCCTGGGGAGTACGGTCG
CAAGACTAAAACTCAAATGAATTGACGGGGGCCCGCACAAGCGGTGGAACCATGT
GGTTTAATTCGATGCAACGCGAAGAACCTTACCTGGCCTTGACATAGTAAGAACTT
TCCAGAGATGGATTGGTGCCTTCGGGAACTTACATACAGGTGCTGCATGGCTGTCG

TCAGCTCGTGTCGTGAGATGTTGGGTTAAGTCCCGCAACGAGCGCAACCCTTTTCC
TTATTTGCCAGCGAGTAATGTCGGGAACTTTAAGGATACTGCCAGTGACAAACTGG
AGGAAGGCGGGGACGACGTCAAGTCATCATGGCCCTTACGGCCAGGGCTACACAC
GTGCTACAATGGTCGGTACAAAGGGTTGCTACCTAGCGATAGGATGCTAATCTCAA
AAAGCCGATCGTAGTCCGGATTGGAGTCTGCAACTCGACTCCATGAAGTCGGAATC
GCTAGTAATCGCGGATCAGAATGCCGCGGTGAATACGTTCCCGGGCCTTGTACACA
CCGCCCGTCACACCATGGGAGTTTGTTGCACCAGAAGTAGCTAGCCTAACTGCAAA
GAGGGCGGTTACCACGGTGTGGCCGATGACTGGGGTGAAGTCGTAACAAGGTAGC
CGTAGGGGAACCTGNGGATGGATCACCTCCTTA
>Beijerinckia fluminensis UQM 1685 (Agrobacterium radiobacter) (NR116306)
AGTTTGATCCTGGCTCAGAACGAACGCTGGCGGCAGGCTTAACACATGCAAGTCGA
ACGCCCCGCAAGGGGAGTGGCAGACGGGTGAGTAACGCGTGGGAACATACCCTTT
CCTGCGGAATAGCTCCGGGAAACTGGAATTAATACCGCATACGCCCTACGGGGGA
AAGATTTATCGGGGAAGGATTGGCCCGCGTTGGATTAGCTAGTTGGTGGGGTAAAG
GCCTACCAAGGCGACGATCCATAGCTGGTCTGAGAGGATGATCAGCCACATTGGG
ACTGAGACACGGCCCAAACTCCTACGGGAGGCAGCAGTGGGGAATATTGGACAAT
GGGCGCAAGCCTGATCCAGCCATGCCGCGTGAGTGATGAAGGCCTTAGGGTTGTA
AAGCTCTTTCACCGGAGAAGATAATGACGGTATCCGGAGAAGAAGCCCCGGCTAA
CTTCGTGCCAGCAGCCGCGGTAATACGAAGGGGGCTAGCGTTGTTCGGAATTACTG
GGCGTAAAGCGCACGTAGGCGGATATTTAAGTCAGGGGTGAAATCCCAGAGCTCA
ACTCTGGAACTGCCTTTGATACTGGGTATCTTGAGTATGGAAGAGGTAAGTGGAAT
TCCGAGTGTAGAGGTGAAATTCGTAGATATTCGGAGGAACACCAGTGGCGAAGGC
GGCTTACTGGTCCATTACTGACGCTGAGGTGCGAAAGCGTGGGGAGCAAACAGGA
TTAGATACCCTGGTAGTCCACGCCGTAAACGATGAATGTTAGCCGTCGGGCAGTAT
ACTGTTCGGTGGCGCAGCTAACGCATTAAACATTCCGCCTGGGGAGTACGGTCGCA
AGATTAAAACTCAAAGGAATTGACGGGGGCCCGCACAAGCGGTGGAGCATGTGGT
TTAATTCGAAGCAACGCGCAGAACCTTACCAGCTCTTGACATTCGGGGTATGGGCA
TTGGAGACGATGTCCTTCAGTTAGGCTGGCCCCAGAACAGGTGCTGCATGGCTGTC
GTCAGCTCGTGTCGTGAGATGTTGGGTTAAGTCCCGCAACGAGCGCAACCCTCGCC
CTTAGTTGCCAGCATTTAGTTGGGCACTCTAAGGGGACTGCCGGTGATAAGCCGAG
AGGAAGGTGGGGATGACGTCAAGTCCTCATGGCCCTTACGGGCTGGGCTACACAC
GTGCTACAATGGTGGTGACAGTGGGCAGCGAGACAGCGATGTCGAGCTAATCTCC
AAAAGCCATCTCAGTTCGGATTGCACTCTGCAACTCGAGTGCATGAAGTTGGAATC
GCTAGTAATCGCAGATCAGCATGCTGCGGTGAATACGTTCCCGGGCCTTGTACACA
CCGCCCGTCACACCATGGGAGTTGGTTTTACCCGAAGGTAGTGCGCTAACCGCAAG
GAGGCAGCTAACCACGGTAGGGTCAGCGACTGGGGTGAAGTCGTAACAAGGTAGC
CGTAA
>Agrobacterium tumefaciens NCPPB2437 (NR115516)
GTTTGATCCTGGCTCAGAACGAACGCTGGCGGCAGGCTTAACACATGCAAGTCGAA
CGCCCCGCAAGGGGAGTGGCAGACGGGTGAGTAACGCGTGGGAACATACCCTTTC
CTGCGGAATAGCTCCGGGAAACTGGAATTAATACCGCATACGCCCTACGGGGGAA
AGATTTATCGGGGAAGGATTGGCCCGCGTTGGATTAGCTAGTTGGTGGGGTAAAGG
CCTACCAAGGCGACGATCCATAGCTGGTCTGAGAGGATGATCAGCCACATTGGGA
CTGAGACACGGCCCAAACTCCTACGGGAGGCAGCAGTGGGGAATATTGGACAATG
GGCGCAAGCCTGATCCAGCCATGCCGCGTGAGTGATGAAGGCCTTAGGGTTGTAA
AGCTCTTTCACCGGAGAAGATAATGACGGTATCCGGAGAAGAAGCCCCGGCTAAC
TTCGTGCCAGCAGCCGCGGTAATACGAAGGGGGCTAGCGTTGTTCGGAATTACTGG
GCGTAAAGCGCACGTAGGCGGATATTTAAGTCAGGGGTGAAATCCCAGAGCTCAA
CTCTGGAACTGCCTTTGATACTGGGTATCTTGAGTATGGAAGAGGTAAGTGGAATT
CCGAGTGTAGAGGTGAAATTCGTAGATATTCGGAGGAACACCAGTGGCGAAGGCG
GCTTACTGGTCCATTACTGACGCTGAGGTGCGAAAGCGTGGGGAGCAAACAGGAT

TAGATACCCTGGTAGTCCACGCCGTAAACGATGAATGTTAGCCGTCGGGCAGTATA
CTGTTCGGTGGCGCACGTAACGCATTAAACATTCCGCCTGGGGAGTACGGTCGCAA
GATTAAAACTCAAAGGAATTGACGGGGGCCCGCACAAGCGGTGGAGCATGTGGTT
TAATTCGAAGCAACGCGCAGAACCTTACCAGCTCTTGACATTCGGGGTTTGGGCAG
TGGAGACATTGTCCTTCAGTTAGGCTGGCCCCAGAACAGGTGCTGCATGGCTGTCG
TCAGCTCGTGTCGTGAGATGTTGGGTTAAGTCCCGCAACGAGCGCAACCCTCGCCC
TTAGTTGCCAGCATTTAGTTGGGCACTCTAAGGGGACTGCCGGTGATAAGCCGAGA
GGAAGGTGGGGATGACGTCAAGTCCTCATGGCCCTTACGGGCTGGGCTACACACGT
GCTACAATGGTGGTGACAGTGGGCAGCGAGACAGCGATGTCGAGCTAATCTCCAA
AAGCCATCTCAGTTCGGATTGCACTCTGCAACTCGAGTGCATGAAGTTGGAATCGC
TAGTAATCGCAGATCAGCATGCTGCGGTGAATACGTTCCCGGGCCTTGTACACACC
GCCCGTCACACCATGGGAGTTGGTTTTACCCGAAGGTAGTGCGCTAACCGCAAGGA
GGCAGCTAACCACGGTAGGGTCAGCGACTGGGGTGAAGTCGTAACAAGGTAGCCG
TAGGGGAACCTGCGGCTGGATCACCTCC
>Agrobacterium larrymoorei AF3.10 (NR026519)
AACGAACGCTGGCGGCAGGCTTAACACATGCAAGTCGAACGCATCGCAAGATGAG
TGGCAGACGGGTGAGTAACGCGTGGGAACATACCCTTTTCTACGGAATAGCTCTGG
GAAACTGGAATTAATACCGTATACGCCCTACGGGGGAAAGATTTATCGGGGAAGG
ATTGGCCCGCGTTGGATTAGCTAGTTGGTGGGGTAAAGGCCTACCAAGGCGACGAT
CCATAGCTGGTCTGAGAGGATGATCAGCCACATTGGGACTGAGACACGGCCCAAA
CTCCTACGGGAGGCAGCAGTGGGGAATATTGGACAATGGGCGCAAGCCTGATCCA
GCCATGCCGCGTGAGTGATGAAGGCCTTAGGGTTGTAAAGCTCTTTCACCGATGAA
GATAATGACGGTAGTCGGAGAAGAAGCCCCGGCTAACTTCGTGCCAGCAGCCGCG
GTAATACGAAGGGGGCTAGCGTTGTTCGGAATTACTGGGCGTAAAGCGCACGTAG
GCGGATATTTAAGTCAGGGGTGAAATCCCAGAGCTCAACTCTGGAACTGCCTTTGA
TACTGGGTATCTTGAGTATGGAAGAGGTAAGTGGAATTGCGAGTGTAGAGGTGAA
ATTCGTAGATATTCGCAGGAACACCAGTGGCGAAGGCGGCTTACTGGTCCATTACT
GACGCTGAGGTGCGAAAGCGTGGGGAGCAAACAGGATTAGATACCCTGGTAGTCC
ACGCCGTAAACGATGAATGTTAGCCGTCGGGCAGTATACTGTTCGGTGGCGCAGNT
AACGCATTAAACATTCCGCCTGGGGAGTACGGTCGCAAGATTAAAACTCAAAGGA
ATTGACGGGGGCCCGCACAAGCGGTGGAGCATGTGGTTTAATTCGAAGCAACGCG
CAGAACCTTACCAGCTCTTGACATTCGGGGTATGGTCATTGGAGACGATGACCTTC
AGTTCGGCTGGCCCTAGAACAGGTGCTGCATGGCTGTCGTCAGCTCGTGTCGTGAG
ATGTTGGGTTAAGTCCCGCAACGAGCGCAACCCTCGCCCTTAGTTGCCAGCATTTG
GTTGGGCACTCTAAGGGGACTGCCGGTGATAAGCCGAGAGGAAGGTGGGGATGAC
GTCAAGTCCTCATGGCCCTTACGGGCTGGGCTACACACGTGCTACAATGGTGGTGA
CAGTGGGCAGCGAGACAGCGATGTCGAGCTAATCTCCAAAAGCCATCTCAGTTCG
GATTGCACTCTGCAACTCGAGTGCATGAAGTTGGAATCGCTAGTAATCGCAGATCA
GCATGCTGCGGTGAATACGTTCCCGGGCCTTGTACACACCGCCCGTCACACCATGG
GAGTTGGTTTTACCCGAAGGCGCTGCGCTAACCGCAAGGGGGCAGGCGACCACGG
TAGGGTCAGCGACTGGGGTGAAGTCGTAACAAGGTAGCCGTAGGGGAACC
>Agrobacterium fabrum C58 (NR074266)
TTGAGAGTTTGATCCTGGCTCAGAACGAACGCTGGCGGCAGGCTTAACACATGCAA
GTCGAACGCCCCGCAAGGGGAGTGGCAGACGGGTGAGTAACGCGTGGGAATCTAC
CCATCTCTGCGGAATAGCTCTGGGAAACTGGAATTAATACCGCATACGCCCTACGG
GGGAAAGATTTATCGGGGATGGATGAGCCCGCGTTGGATTAGCTAGTTGGTGGGGT
AAAGGCCTACCAAGGCGACGATCCATAGCTGGTCTGAGAGGATGATCAGCCACAT
TGGGACTGAGACACGGCCCAAACTCCTACGGGAGGCAGCAGTGGGGAATATTGGA
CAATGGGCGCAAGCCTGATCCAGCCATGCCGCGTGAGTGATGAAGGCCTTAGGGTT
GTAAAGCTCTTTCACCGATGAAGATAATGACGGTAGTCGGAGAAGAAGCCCCGGC
TAACTTCGTGCCAGCAGCCGCGGTAATACGAAGGGGGCTAGCGTTGTTCGGAATTA

CTGGGCGTAAAGCGCACGTAGGCGGATATTTAAGTCAGGGGTGAAATCCCGCAGC
TCAACTGCGGAACTGCCTTTGATACTGGGTATCTTGAGTATGGAAGAGGTAAGTGG
AATTCCGAGTGTAGAGGTGAAATTCGTAGATATTCGGAGGAACACCAGTGGCGAA
GGCGGCTTACTGGTCCATTACTGACGCTGAGGTGCGAAAGCGTGGGGAGCAAACA
GGATTAGATACCCTGGTAGTCCACGCCGTAAACGATGAATGTTAGCCGTCGGGCAG
TATACTGTTCGGTGGCGCAGCTAACGCATTAAACATTCCGCCTGGGGAGTACGGTC
GCAAGATTAAAACTCAAAGGAATTGACGGGGGCCCGCACAAGCGGTGGAGCATGT
GGTTTAATTCGAAGCAACGCGCAGAACCTTACCAGCTCTTGACATTCGGGGTATGG
GCATTGGAGACGATGTCCTTCAGTTAGGCTGGCCCCAGAACAGGTGCTGCATGGCT
GTCGTCAGCTCGTGTCGTGAGATGTTGGGTTAAGTCCCGCAACGAGCGCAACCCTC
GCCCTTAGTTGCCAGCATTTAGTTGGGCACTCTAAGGGGACTGCCGGTGATAAGCC
GAGAGGAAGGTGGGGATGACGTCAAGTCCTCATGGCCCTTACGGGCTGGGCTACA
CACGTGCTACAATGGTGGTGACAGTGGGCAGCGAGACAGCGATGTCGAGCTAATC
TCCAAAAGCCATCTCAGTTCGGATTGCACTCTGCAACTCGAGTGCATGAAGTTGGA
ATCGCTAGTAATCGCAGATCAGCATGCTGCGGTGAATACGTTCCCGGGCCTTGTAC
ACACCGCCCGTCACACCATGGGAGTTGGTTTACCCGAAGGTAGTGCGCTAACCGC
AAGGAGGCAGCTAACCACGGTAGGGTCAGCGACTGGGGTGAAGTCGTAACAAGGT
AGCCGTAGGGGAACCTGCGGCTGGATCACCTCCTT
>Sphingobium yanoikuyae NBRC 15102 (NR113730)
AACGAACGCTGGCGGCATGCCTAATACATGCAAGTCGAACGAGATCTTCGGATCTA
GTGGCGCACGGGTGCGTAACGCGTGGGAATCTGCCCTTGGGTTCGGAATAACTTCT
GGAAACGGAAGCTAATACCGGATGATGACGTAAGTCCAAAGATTTATCGCCCAAG
GATGAGCCCGCGTAGGATTAGCTAGTTGGTGAGGTAAAGGCTCACCAAGGCGACG
ATCCTTAGCTGGTCTGAGAGGATGATCAGCCACACTGGGACTGAGACACGGCCCA
GACTCCTACGGGAGGCAGCAGTAGGGAATATTGGACAATGGGCGAAAGCCTGATC
CAGCAATGCCGCGTGAGTGATGAAGGCCTTAGGGTTGTAAAGCTCTTTTACCCGGG
ATGATAATGACAGTACCGGGAGAATAAGCTCCGGCTAACTCCGTGCCAGCAGCCG
CGGTAATACGGAGGGAGCTAGCGTTGTTCGGAATTACTGGGCGTAAAGCGCACGT
AGGCGGCTATTCAAGTCAGAGGTGAAAGCCCGGGGCTCAACCCCGGAACTGCCTTT
GAAACTAGATAGCTTGAATCCAGGAGAGGTGAGTGGAATTCCGAGTGTAGAGGTG
AAATTCGTAGATATTCGGAAGAACACCAGTGGCGAAGGCGGCTCACTGGACTGGT
ATTGACGCTGAGGTGCGAAAGCGTGGGGAGCAAACAGGATTAGATACCCTGGTAG
TCCACGCCGTAAACGATGATAACTAGCTGTCAGGGCACATGGTGTTTTGGTGGCGC
AGCTAACGCATTAAGTTATCCGCCTGGGGAGTACGGTCGCAAGATTAAAACTCAAA
GGAATTGACGGGGGCCTGCACAAGCGGTGGAGCATGTGGTTTAATTCGAAGCAAC
GCGCAGAACCTTACCAACGTTTGACATCCCTATCGCGGATCGTGGAGACACTTTCC
TTCAGTTCGGCTGGATAGGTGACAGGTGCTGCATGGCTGTCGTCAGCTCGTGTCGT
GAGATGTTGGGTTAAGTCCCGCAACGAGCGCAACCCTCGCCTTTAGTTGCCAGCAT
TTAGTTGGGTACTCTAAAGGAACCGCCGGTGATAAGCCGGAGGAAGGTGGGGATG
ACGTCAAGTCCTCATGGCCCTTACGCGTTGGGCTACACACGTGCTACAATGGCGAC
TACAGTGGGCAGCCACCTCGCGAGAGGGAGCTAATCTCCAAAAGTCGTCTCAGTTC
GGATCGTTCTCTGCAACTCGAGAGCGTGAAGGCGGAATCGCTAGTAATCGCGGATC
AGCATGCCGCGGTGAATACGTTCCCAGGCCTTGTACACACCGCCCGTCACACCATG
GGAGTTGGATTCACTCGAAGGCGTTGAGCTAACCGTAAGGAGGCAGGCGACCACA
GTGGGTTTAGCGACTGGGGTGAAG
>Sphingobium yanoikuyae GIFU 9882 (NR115524)
GAACGAACGCTGGCGGCATGCCTAATACATGCAAGTCGAACGAGATCTTCGGATCT
AGTGGCGCACGGGTGCGTAACGCGTGGGAATCTGCCCTTGGGTTCGGAATAACTTC
TGGAAACGGAAGCTAATACCGGATGATGACGTAAGTCCAAAGATTTATCGCCCAA
GGATGAGCCCGCGTAGGATTAGCTAGTTGGTGAGGTAAAGGCTCACCAAGGCGAC
GATCCTTAGCTGGTCTGAGAGGATGATCAGCCACACTGGGACTGAGACACGGCCC

AGACTCCTACGGGAGGCAGCAGTAGGGAATATTGGACAATGGGCGAAAGCCTGAT
CCAGCAATGCCGCGTGAGTGATGAAGGCCTTAGGGTTGTAAAGCTCTTTTACCCGG
GATGATAATGACAGTACCGGGAGAATAAGCTCCGGCTAACTCCGTGCCAGCAGCC
GCGGTAATACGGAGGGAGCTAGCGTTGTTCGGAATTACTGGGCGTAAAGCGCACG
TAGGCGGCTATTCAAGTCAGAGGTGAAAGCCCGGGGCTCAACCCCGGAACTGCCTT
TGAAACTAGATAGCTTGAATCCAGGAGAGGTGAGTGGAATTCCGAGTGTAGAGGT
GAAATTCGTAGATATTCGGAAGAACACCAGTGGCGAAGGCGGCTCACTGGACTGG
TATTGACGCTGAGGTGCGAAAGCGTGGGGAGCAAACAGGATTAGATACCCTGGTA
GTCCACGCCGTAAACGATGATAACTAGCTGTCAGGGCACATGGTGTTTTGGTGGCG
CACGTAACGCATTAAGTTATCCGCCTGGGGAGTACGGTCGCAAGATTAAAACTCAA
AGGAATTGACGGGGGCCTGCACAAGCGGTGGAGCATGTGGTTTAATTCGAAGCAA
CGCGCAGAACCTTACCAACGTTTGACATCCCTATCGCGGATCGTGGAGACACTTTC
CTTCAGTTCGGCTGGATAGGTGACAGGTGCTGCATGGCTGTCGTCAGCTCGTGTCG
TGAGATGTTGGGTTAAGTCCCGCAACGAGCGCAACCCTCGCCTTTAGTTGCCAGCA
TTTAGTTGGGTACTCTAAAGGANACCGCCGGTGATAAGCCGGAGGAAGGTGGGGA
TGACGTCAAGTCCTCATGGCCCTTACGCGTTGGGCTACACACGTGCTACAATGGCG
ACTACAGTGGGCAGCCACCTCGCGAGGAGGGAGCTAATCTCCAAAAGTCGTCTCA
GTTCGGATCGTTCTCTGCAACTCGAGAGCGTGAAGGCGGAATCGCTAGTAATCGCG
GATCAGCATGCCGCGGTGAATACGTTTCCAGGCCTTGTACACACCGCCCGTCACAC
CATGGGAGTTGGATTCACTCGAAGGCGTTGAGCTAACCGTAAGGAGGCAGGCGAC
CACAGTGGGTTTAGCGACTGGGGTGAAGTCGTAACAAGGTAGCCGTAGGGGAACC
TGCGG
>Sphingobium yanoikuyae IFO 15102 (NR036767)
AGTTTGATCCTGGCTCAGAACGAACGCTGGCGGCATGCCTAATACATGCAAGTCGA
ACGAGATCTTCGGATCGAGTGGCCCACGGGTGCGTAACGCGTGGGAATCTGCCCTT
GGGTTCGGAATAACTTCTGGAAACGGAAGCTAATACCGGATGATGACGTAGGTCC
AAAGATTTATCGCCCAAGGATGAGCCCGCGTAGGATTAGCTAGTTGGTGAGGTAA
AGGCTCACCAAGGCGACGATCCTTAGCTGGTCTGAGAGGATGATCAGCCACACTG
GGACTGAGACACGGCCCAGACTCCTACGGGAGGCAGCAGTGGGGAATATTGGACA
ATGGGCGAAAGCCTGATCCAGCAATGCCGCGTGAGTGATGAAGGCCTTAGGGTTG
TAAAGCTCTTTTACCCGGGATGATAATGACAGTACCGGGAGAATAAGCTCCGGCTA
ACTCCGTGCCAGCAGCCGCGGTAATACGGAGGGAGCTAGCGTTGTTCGGAATTACT
GGGCGTAAAGCGCACGTAGGCGGCTATTCAAGTCAGAGGTGAAAGCCCGGGGCTC
AACCCCGGAACTGCCTTTGAAACTAGATAGCTTGAATCCAGGAGAGGTGAGTGGA
ATTCCGAGTGTAGAGGTGAAATTCGTAGATATTCGGAAGAACACCAGTTGCGAAG
GCGGCTCACTGGACTGGTATTGACGCTGAGGTGCGAAAGCGTGGGGAGCAAACAG
GATTAGATACCCTGGTAGTCCACGCCGTAAACGATGATAACTAGCTGTCAGGGCAC
ATGGTGTTTTGGTGGCGCACGTAACGCATTAAGTTATCCGCCTGGGGAGTACGGTC
GCAAGATTAAAACTCAAAGGAATTGACGGGGGCCTGCACAAGCGGTGGAGCATGT
GGTTTAATTCGAAGCAACGCGCAGAACCTTACCAACGTTTGACATCCCTATCGCGG
ATCGTGGAGACACTTTCCTTCAGTTCGGCTGGATAGGTGACAGGTGCTGCATGGCT
GTCGTCAGCTCGTGTCGTGAGATGTTGGGTTAAGTCCCGCAACGAGCGCAACCCTC
GCCTTTAGTTGCCTGCATTTAGTTGGGTACTCTAAAGGAACCGCCGGTGATAAGCC
GGAGGAAGGTGGGGATGACGTCAAGTCCTCATGGCCCTTACGCGTTGGGCTACAC
ACGTGCTACAATGGCGACTACAGTGGGCAGCCACCTCGCGAGAGGGAGCTAATCT
CCAAAAGTCGTCTCAGTTCGGATCGTTCTCTGCAACTCGAGAGCGTGAAGGCGGAA
TCGCTAGTAATCGCGGATCAGCATGCCGCGGTGAATACGTTCCCAGGCCTTGTACA
CACCGCCCGTCACACCATGGGAGTTGGATTCACTCGAAGGCGTTGAGCTAACCGTA
AGGAGGCAGGCGACCACACAGTGGGTC
>Sphingobium herbicidovorans NBRC 16415 (NR113843)

AACGAACGCTGGCGGCATGCCTAATACATGCAAGTCGAACGAGATCTTCGGATCTA
GTGGCGCACGGGTGCGTAACGCGTGGGAATCTGCCCTTGGGTTCGGAATAACAGTT
GGAAACGACTGCTAATACCGGATGATGACGTAAGTCCAAAGATTTATCGCCCAAG
GATGAGCCCGCGTAGGATTAGCTAGTTGGTGAGGTAAAGGCTCACCAAGGCTACG
ATCCTTAGCTGGTCTGAGAGGATGATCAGCCACACTGGGACTGAGACACGGCCCA
GACTCCTACGGGAGGCAGCAGTAGGGAATATTGGACAATGGGCGAAAGCCTGATC
CAGCAATGCCGCGTGAGTGATGAAGGCCTTAGGGTTGTAAAGCTCTTTTACCCGGG
ATGATAATGACAGTACCGGGAGAATAAGCCCCGGCTAACTCCGTGCCAGCAGCCG
CGGTAATACGGAGGGGGCTAGCGTTGTTCGGAATTACTGGGCGTAAAGCGCACGT
AGGCGGCGATTTAAGTCAGAGGTGAAAGCCCGGGGCTCAACCCCGGAATAGCCTT
TGAGACTGGATTGCTTGAACATCGGAGAGGTGAGTGGAATTCCGAGTGTAGAGGT
GAAATTCGTAGATATTCGGAAGAACACCAGTGGCGAAGGCGGCTCACTGGACGAT
TGTTGACGCTGAGGTGCGAAAGCGTGGGGAGCAAACAGGATTAGATACCCTGGTA
GTCCACGCCGTAAACGATGATAACTAGCTGCTGGGGTGCATGGCATTTCAGTGGCG
CAGCTAACGCATTAAGTTATCCGCCTGGGGAGTACGGTCGCAAGATTAAAACTCAA
AGGAATTGACGGGGGCCTGCACAAGCGGTGGAGCATGTGGTTTAATTCGAAGCAA
CGCGCAGAACCTTACCAACGTTTGACATCCCTATCGCGGATCGTGGAGACACTTTC
CTTCAGTTCGGCTGGATAGGTGACAGGTGCTGCATGGCTGTCGTCAGCTCGTGTCG
TGAGATGTTGGGTTAAGTCCCGCAACGAGCGCAACCCTCGCCTTTAGTTGCCATCA
TTTAGTTGGGTACTCTAAAGGAACCGCCGGTGATAAGCCGGAGGAAGGTGGGGAT
GACGTCAAGTCCTCATGGCCCTTACGCGTTGGGCTACACACGTGCTACAATGGCGA
CTACAGTGGGCAGCCACTCCGCGAGGAGGAGCTAATCTCCAAAAGTCGTCTCAGTT
CGGATCGTTCTCTGCAACTCGAGAGCGTGAAGGCGGAATCGCTAGTAATCGCGGAT
CAGCATGCCGCGGTGAATACGTTCCCAGGCCTTGTACACACCGCCCGTCACACCAT
GGGAGTTGGTTTCACCCGAAGGCAGTGCGCTAACCGCAAGGAGGCAGCTGACCAC
GGTGGGATCAGCGACTGGGGTGAAG
>Sphingobium ummariense RL-3 (NR044171)
CAGAACGAACGCTGGCGGCATGCCTAATACATGCAAGTCGAACGAAGGCTTCGGC
CTTAGTGGCGCACGGGTGCGTAACGCGTGGGAATCTGCCCTTGGGTTCGGAATAAC
GTCTGGAAACGGACGCTAATACCGGATGATGACGTAAGTCCAAAGATTTATCGCCC
AGGGATGAGCCCGCGTAGGATTAGCTAGTTGGTGAGGTAAAGGCTCACCAAGGCG
ACGATCCTTAGCTGGTCTGAGAGGATGATCAGCCACACTGGGACTGAGACACGGC
CCAGACTCCTACGGGAGGCAGCAGTAGGGAATATTGGACAATGGGCGAAAGCCTG
ATCCAGCAATGCCGCGTGAGTGATGAAGGCCTTAGGGTTGTAAAGCTCTTTTACCC
GGGATGATAATGACAGTACCGGGAGAATAAGCCCCGGCTAACTCCGTGCCAGCAG
CCGCGGTAATACGGAGGGGGCTAGCGTTGTTCGGAATTACTGGGCGTAAAGCGCA
CGTAGGCGGCGATTTAAGTCAGAGGTGAAAGCCCGGGGCTCAACCCCGGAATAGC
CTTTGAGACTGGATTGCTTGAATCCGGGAGAGGTGAGTGGAATTCCGAGTGTAGAG
GTGAAATTCGTAGATATTCGGAAGAACACCAGTGGCGAAGGCGGCTCACTGGACC
GGCATTGACGCTGAGGTGCGAAAGCGTGGGGAGCAAACAGGATTAGATACCCTGG
TAGTCCACGCCGTAAACGATGATAACTAGCTGCTGGGGCTCATGGAGTTTCAGTGG
CGCAGCTAACGCATTAAGTTATCCGCCTGGGGAGTACGGTCGCAAGATTAAAACTC
AAAGGAATTGACGGGGGCCTGCACAAGCGGTGGAGCATGTGGTTTAATTCGAAGC
AACGCGCAGAACCTTACCAACGTTTGACATCCCTAGTATGGTTACCAGAGATGGTT
TCCTTCAGTTCGGCTGGCTAGGTGACAGGTGCTGCATGGCTGTCGTCAGCTCGTGTC
GTGAGATGTTGGGTTAAGTCCCGCAACGAGCGCAACCCTCGCCTTTAGTTGCCATC
ATTCAGTTGGGTACTCTAAAGGAACCGCCGGTGATAAGCCGGAGGAAGGTGGGGA
TGACGTCAAGTCCTCATGGCCCTTACGCGTTGGGCTACACACGTGCTACAATGGCG
ACTACAGTGGGCAGCTATCTCGCGAGAGTGCGCTAATCTCCAAAAGTCGTCTCAGT
TCGGATCGTTCTCTGCAACTCGAGAGCGTGAAGGCGGAATCGCTAGTAATCGCGGA
TCAGCATGCCGCGGTGAATACGTTCCCAGGCCTTGTACACACCGCCCGTCACACCA

TGGGAGTTGGTTTCACCCGAAGGCGCTGCGCTAACTCGCAAGAGAGGCAGGCGAC
CACGGTGGGATCAGCGACTGGGGTGAAGTC

Aligned and cut

>OTU586 (Sphingobium yanoikuyae)
AACGAACGCTGGCGGCATGCCTAATACATGCAAGTCGAACGAGA--------------
TCTTCGGATCTAGTGGCGCACGGGTGCGTAACGCGTGGG-
AATCTGCCCTTGGGTTCGGAATAACTTCTGGAAACGGAAGCTAATACCGGATG-
ATGACGTAAGTCCAAAG--------------
ATTTATCGCCCAAGGATGAGCCCGCGTAGGATTAGCTAGTTGGTGGGGTAAAGGCT
CACCAAGGCGACGATCCTTAGCTGGTCTGAGAGGATGATCAGCCACACTGGGACT
GAGACACGGCCCAGACTCCTACGGGAGGCAGCAGTAGGGAATATTGGACAATGGG
CGAAAGCCTG-ATCCAGCAATGCCGCGTGAGTGATGAAGGCCTTAGGG-
TTGTAAAGCTCTTTTACCCGGGATGA------------TAATG--------------ACAGT-ACCGG-
AGAATAAGCTCCGGCTAACTCCGTGCC
>OTU2089 (Agrobacterium radiobacter)
AACGAACGCTGGCGGCAGGCTTAACACATGCAAGTCGAACG----------------
CCCCGCAAGGGGAGTGGCAGACGGGTGAGTAACGCGTGGG-
AACATACCCTTTCCTGCGGAATAGCTCCGGGAAACTGGAATTAATACCGCATA-
CGCCCTACGGGGGAAAG--------------
ATTTATCGGGGAAGGATTGGCCCGCGTTGGATTAGCTAGTTGGTGGGGTAAAGGCC
TACCAAGGCGACGATCCATAGCTGGTCTGAGAGGATGATCAGCCACATTGGGACT
GAGACACGGCCCAAACTCCTACGGGAGGCAGCAGTGGGGAATATTGGACAATGGG
CGCAAGCCTG-ATCCAGCCATGCCGCGTGAGTGATGAAGGCCTTAGGG-
TTGTAAAGCTCTTTCACCGATGAAGA------------TAATG--------------ACGGT-
AGTCGGAGAAGAAGCCCCGGCTAACTTCGTGCC
>OTU1492 (Acinetobacter)
ATTGAACGCTGGCGGCAGGCTTAACACATGCAAGTCGAGCGGAGAGA-
GGTAGCTTGCTACTG-ATCTTAGCGGCGGACGGGTGAGTAATGCTTAGG-
AATCTGCCTATTAGTGGGGGACAACATTTCGAAAGGAATGCTAATACCGCATA-
CGTCCTACGGGAGAAAGCAGGGGATCTTCGGACCTTGCGCTAATAGATGAGCCTA
AGTCGGATTAGCTAGTTGGTGGG-
TAAAGGCCTACCAAGGCGACGATCTGTAGCGGGTCTGAGAGGATGATCCGCCACA
CTGGGACTGAGACACGGCCCAGACTCCTACGGGAGGCAGCAGTGGGGAATATTGG
ACAATGGGCGCAAGCCTG-
ATCCAGCCATGCCGCGTGTGTGAAGAAGGCCTTATGG-
TTGTAAAGCACTTTAAGCGAGGAGGAGGCTACTTTAG-
TAATACCTAGAGATAGTGGACGTT-
ACTCGCAGAATAAGCACCGGCTAACTCCGTAGC
>OTU47 (Chryseobacterium)
GATGGACGCTAGCGGGAGGCCTAACACATGCAAGCCGAGCGGTAGAG----
TTTCTTCGGAAACTTGAGAGCGGCGCACGGGTGCGTAACACGTGTGCAACCTACCT
TTATCTGGGGGATAGCCTTTCGAAAGGAAGATTAATACCCCATAATATAATGATTG
GCATCAATTATTATTGAAAACTCCGGTGGATAGAGATGGGCACGCGCAAGATTAG
ATAGTTGGTGAGGTAACGGCTCACCAAGTCAATGATCTTTAGGGGTCCTGAGAGGG
AGATCCCCCACACTGGTACTGAGACACGGACCAGACTCCTACGGGAGGCAGCAGT
GAGGAATATTGGACAATGGGTGAGAGCCTG-
ATCCAGCCATCCCGCGTGAAGGATGACGGTCCTATGGATTGTAAACTTCTTTTGTA

CAGGGATAAACCTACTCTCGTGAGG-------GTAGCTGAAGGT-
ACTGTACGAATAAGCACCGGCTAACTCCGTGCC
>Chryseobacterium haifense H38 (NR_044167)
GATGAACGCTAGCGGGAGGCCTAACACATGCAAGCCGAGCGGTAGAG----
TTTCTTCGGAAACTTGAGAGCGGCGCACGGGTGCGTAACACGTGTGCAACCTACCT
TTATCTGGGGGATAGCCTTTCGAAAGGAAGATTAATACCCCATAATATAATGATTG
GCATCAATTATTATTGAAAACTCCGGTGGATAGAGATGGGCACGCGCAAGATTAG
ATAGTTGGTGAGGTAACGGCTCACCAAGTCAATGATCTTTAGGGGTCCTGAGAGGG
AGATCCCCCACACTGGTACTGAGACACGGACCAGACTCCTACGGGAGGCAGCAGT
GAGGAATATTGGACAATGGGTGAGAGCCTG-
ATCCAGCCATCCCGCGTGAAGGATGACGGTCCTATGGATTGTAAACTTCTTTTGTA
CAGGGATAAACCTACTCTCGTGAGG-------GTAGCTGAAGGT-
ACTGTACGAATAAGCACCGGCTAACTCCGTGCC
>OTU2007 (Pseudomonas)
ATTGAACGCTGGCGGCAGGCCTAACACATGCAAGTCGAGCGGATGAT-
GGGAGCTTGCTCCTG-GATTCAGCGGCGGACGGGTGAGTAATGCCTAGG-
AATCTGCCTGGTAGTGGGGGACAACGTTTCGAAAGGAACGCTAATACCGCATA-
CGTCCTACGGGAGAAAGCAGGGGACCTTCGGGCCTTGCGCTATCAGATGAGCCTA
GGTCGGATTAGCTTGTTGGTGAGGTAATGGCTCACCAAGGCGACGATCCGTAACTG
GTCTGAGAGGATGATCAGTCACACTGGAACTGAGACACGGTCCAGACTCCTACGG
GAGGCAGCAGTGGGGAATATTGGACAATGGGCGAAAGCCTG-
ATCCAGCCATGCCGCGTGTGTGAAGAAGGTCTTCGGA-
TTGTAAAGCACTTTAAGTTGGGAGGAAGGGTTGTAGATTAATACTCTGCAATTTTG-
ACGTTTACCGACAGAATAAGCACCGGCTAACTCTGTGCC
>OTU99 (Moraxella)
ATTGAACGCTGGCGGCAGGCTTAACACATGCAAGTCGAACGATGACTCTCTAGCTT
GCTAGAGATGATTAGTGGCGGACGGGTGAGTAACATTTAGG-
AATCTACCTAGTAGTGGGGGATAGCTCGGGGAAACTCGAATTAATACCGCATA-
CGACCTACGGGTGAAAGGGGGCG-----CAAGCTCT-
TGCTATTAGATGAGCCTAAATCAGATTAGCTAGTTGGTGGGGTAAAGGCCCACCAA
GGCGACGATCTGTAACTGGTCTGAGAGGATGATCAGTCACACCGGAACTGAGACA
CGGTCCGGACTCCTACGGGAGGCAGCAGTGGGGAATATTGGACAATGGGGGCAAC
CCTG-ATCCAGCCATGCCGCGTGTGTGAAGAAGGCCTTTTGG-
TTGTAAAGCACTTTAAGCAGGGAGGAGAGGCTAATGGTTAATACCCATTAGATTAG
-ACGTT-ACCTGCAGAATAAGCACCGGCTAACTCTGTGCC
>OTU3112 (Chryseobacterium)
GATGAACGCTAGCGGGAGGCCTAACACATGCAAGCCGAGCGGTATTT----
GTTCTTCGGAACAGAGAGAGCGGCGCACGGGTGCGGAACACGTGTGCAACCTGCC
TTTATCTGGGGGATAGCCTTTCGAAAGGAAGATTAATACCCCATAATATATTGAGT
GGCATCATTTGATATTGAAAACTCCGGTGGATAGAGATGGGCACGCGCAAGATTA
GATAGTTGGTGAGGTAACGGCTCACCAAGTCAATGATCTTTAGGGGGCCTGAGAG
GGTGATCCCCCACACTGGTACTGAGACACGGACCAGACTCCTACGGGAGGCAGCA
GTGAGGAATATTGGACAATGGGTTAGCGCCTG-
ATCCAGCCATCCCGCGTGAAGGACGACGGCCCTATGGGTTGTAAACTTCTTTTGTA
TAGGGATAAACCTACTCTCGTGAGA-------GTAGCTGAAGGT-
ACTATACGAATAAGCACCGGCTAACTCCGTGCC
>OTU1844 (Brevundimonas)
AGCGAACGCTGGCGGCAGGCCTAACACATGCAAGTCGAACGGA--------------
CCCTTCGGGGTTAGTGGCGGACGGGTGAGTAACACGTGGG-
AACGTGCCTTTAGGTTCGGAATAGCTCCTGGAAACGGGTGGTAATGCCGAATG-
TGCCCTTCGGGGGAAAG--------------

ATTTATCGCCTTTAGAGCGGCCCGCGTCTGATTAGCTAGTTGGTGAGGTAACGGCT
CACCAAGGCGACGATCAGTAGCTGGTCTGAGAGGATGACCAGCCACACTGGGACT
GAGACACGGCCCAGACTCCTACGGGAGGCAGCAGTGGGGAATCTTGCGCAATGGG
CGAAAGCCTG-ACGCAGCCATGCCGCGTGAATGATGAAGGTCTTAGGA-
TTGTAAAATTCTTTCACCGGGGACGA------------TAATG--------------ACGGT-
ACCCGGAGAAGAAGCCCCGGCTAACTTCGTGCC
>OTU893 (Acinetobacter)
ATTGAACGCTGGCGGCAGGCTTAACACATGCAAGTCGAGCGGGGAAA-
GGTAGCTTGCTACCT-GACCTAGCGGCGGACGGGTGAGTAATGCTTAGG-
AATCTGCCTATTAGTGGGGGACAACATTCCGAAAGGAATGCTAATACCGCATA-
CGCCCTACGGGGGAAAGCAGGGGATCTTCGGACCTTGCGCTAATAGATGAGCCTA
AGTCAGATTAGCTAGTTGGTGGGGTAAAGGCCTACCAAGGCGACGATCTGTAGCG
GGTCTGAGAGGATGATCCGCCACACTGGGACTGAGACACGGCCCAGACTCCTACG
GGAGGCAGCAGTGGGGAATATTGGACAATGGGCGAAAGCCTG-
ATCCAGCCATGCCGCGTGTGTGAAGAAGGCCTTTTGG-
TTGTAAAGCACTTTAAGCGAGGAGGAGGCTACTTAGATTAATACTCTAGGATAGTG
GACGTT-ACTCGCAGAATAAGCACCGGCTAACTCTGTGCC
>OTU2385 (Acinetobacter)
ATTGAACGCTGGCGGCAGGCTTAACACATGCAAGTCGAGCGGAGAGA-
AGTAGCTTGCTACTG-ATCTTAGCGGCGGACGGGTGAGTAATACTTAGG-
AATCTGGCTATTAGTGGGGGACAACATTTCGAAAGGGATGCTAATACCGCATA-
CGTCCTACGGGAGAAAGCAGGGGATCTTCGGACCTTGCGCTAATAGATGAGCCTA
AGTCGGATTAGCTAGTTGGTGGGGTAAAGGCCTACCAAGGCGACGATCTGTAGCG
GGTCTGAGAGGATGATCCGCCACACTGGGACTGAGACACGGCCCAGACTCCTACG
GGAGGCAGCAGTGGGGAATATTGGACAATGGGGGGAACCCTGGATCCAGCCATGC
CGCGTGTGTGAAGAAGGCCTTATGG-
TTGTAAAGCACTTTAAGCGAGGAGGAGGGTACTGGTATTAATACTACCAGGTACTG
GACGTT-ACTCGCAGAATAAGCACCGGCTAACTCTGTGCC
>Moraxella osloensis DSM 6998 (NR113392)
ATTGAACGCTGGCGGCAGGCTTAACACATGCAAGTCGAACGATGACTCTCTAGCTT
GCTAGAGATGATTAGTGGCGGACGGGTGAGTAACATTTAGG-
AATCTACCTAGTAGTGGGGGATAGCTCGGGGAAACTCGAATTAATACCGCATA-
CGACCTACGGGTGAAAGGGGGCG-----CAAGCTCT-
TGCTATTAGATGAGCCTAAATCAGATTAGCTAGTTGGTGGGGTAAAGGCCCACCAA
GGCGACGATCTGTAACTGGTCTGAGAGGATGATCAGTCACACCGGAACTGAGACA
CGGTCCGGACTCCTACGGGAGGCAGCAGTGGGGAATATTGGACAATGGGGGCAAC
CCTG-ATCCAGCCATGCCGCGTGTGTGAAGAAGGCCTTTTGG-
TTGTAAAGCACTTTAAGCAGGGAGGAGAGGCTAATGGTTAATACCCATTAGATTAG
-ACGTT-ACCTGCAGAATAAGCACCGGCTAACTCTGTGCC
>Moraxella osloensis ATCC 19976 (NR113391)
ATTGAACGCTGGCGGCAGGCTTAACACATGCAAGTCGAACGATGACTCTCTAGCTT
GCTAGAGATGATTAGTGGCGGACGGGTGAGTAACATTTAGG-
AATCTACCTAGTAGTGGGGGATAGCTCGGGGAAACTCGAATTAATACCGCATA-
CGACCTACGGGTGAAAGGGGGCG-----CAAGCTCT-
TGCTATTAGATGAGCCTAAATCAGATTAGCTAGTTGGTGGGGTAAAGGCCCACCAA
GGCGACGATCTGTAACTGGTCTGAGAGGATGATCAGTCACACCGGAACTGAGACA
CGGTCCGGACTCCTACGGGAGGCAGCAGTGGGGAATATTGGACAATGGGGGCAAC
CCTG-ATCCAGCCATGCCGCGTGTGTGAAGAAGGCCTTTTGG-
TTGTAAAGCACTTTAAGCAGGGAGGAGAGGCTAATGGTTAATACCCATTAGATTAG
-ACGTT-ACCTGCAGAATAAGCACCGGCTAACTCTGTGCC
>Moraxella osloensis A1920 (NR104936)

ATTGAACGCTGGCGGCAGGCTTAACACATGCAAGTCGAACGATGACTCTCTAGCTT
GCTAGAGATGATTAGTGGCGGACGGGTGAGTAACATTTAGG-
AATCTACCTAGTAGTGGGGGATAGCTCGGGGAAACTCGAATTAATACCGCATA-
CGACCTACGGGTGAAAGGGGGCG-----CAAGCTCT-
TGCTATTAGATGAGCCTAAATCAGATTAGCTAGTTGGTGGGGTAAAGGCCCACCAA
GGCGACGATCTGTAACTGGTCTGAGAGGATGATCAGTCACACCGGAACTGAGACA
CGGTCCGGACTCCTACGGGAGGCAGCAGTGGGGAATATTGGACAATGGGGGCAAC
CCTG-ATCCAGCCATGCCGCGTGTGTGAAGAAGGCCTTTTGG-
TTGTAAAGCACTTTAAGCAGGGAGGAGAGGCTAATGGTTAATACCCATTAGATTAG
-ACGTT-ACCTGCAGAATAAGCACCGGCTAACTCTGTGCC
>Enhydrobacter aerosaccus G (NR029005)
ATTGAACGCTGGCGGCAGGCTTAACACATGCAAGTCGAACGATGACTCTCTAGCTT
GCTAGAGATGATTAGTGGCGGACGGGTGAGTAATATTTAGG-
AATCTGCCTAGTAGTGGGGGATAGCTCGGGGAAACTCGAATTAATACCGCATA-
CGACCTACGGGTGAAAGGGGGCG-----CAAGMTCT-
TGCTATTAGATGAGCCTAAATCAGATTAGCTAGTTGGTGGGGTAAAGGCCCACCAA
GGCGACGATCTGTAACTGGTCTGAGAGGATGATCAGTCACACCGGAACTGAGACA
CGGTCCGGACTCCTACGGGAGGCAGCAGTGGGGAATATTGGACAATGGGGGCAAC
CCTG-ATCCAGCCATGCCGCGTGTGTGAAGAAGGCCTTTTGG-
TTGTAAAGCACTTTAAGCAGGGAGGAGAGGCTAATGGTTAATACCCATTAGATTAG
-ACGTT-ACCTGCAGAATAAGCACCGGCTAACTCTGTGCC
>Moraxella lincolnii LMG 5127 (NR117693)
ATTGAACGCTGGCGGCAGGCTTAACACATGCAAGTCGAACGAAGAGG-
TCTAGCTTGCTAGAC-TGATTAGTGGCGAACGGGTGAGTAACATTTAGG-
AATCTACCTTATAGAGGGGGATAGCTCGGGGAAACTCGAATTAATACCGCATA-
CGACCTACGGGTGAAAGGGGGCG-----CAAGCTCT-
TGCTATAAGATGAGCCTAAACCAGATTAGCTAGTTGGTGGGGTAAAGGCCTACCAA
GGCGACGATCTGTAGCTGGTCTGAGAGGATGATCAGCCACACTGGGACTGAGACA
CGGCCCAGACTCCTACGGGAGGCAGCAGTGGGGAATATTGGACAATGGGCGAAAG
CCTG-ATCCAGCCATGCCGCGTGTGTGAAGAAGGCCTTATGG-
TTGTAAAGCACTTTAAGTGGGGAGGAAAAGCTTATGGATAACACCCATAGGTTCTG
-ACGTT-ACCCACAGAATAAGCACCGGCTAACTCTGTGCC
>Moraxella lincolnii CCUG 9405 (NR117189)
ATTGAACGCTGGCGGCAGGCTTAACACATGCAAGTCGAACGAAGAGG-
TCTAGCTTGCTAGAC-TGATTAGTGGCGAACGGGTGAGTAACATTTAGG-
AATCTACCTTATAGAGGGGGATAGCTCGGGGAAACTCGAATTAATACCGCATA-
CGACCTACGGGTGAAAGGGGGCG-----CAAGCTCT-
TGCTATAAGATGAGCCTAAACCAGATTAGCTAGTTGGTGGGGTAAAGGCCTACCAA
GGCGACGATCTGTAGCTGGTCTGAGAGGATGATCAGCCACACTGGGACTGAGACA
CGGCCCAGACTCCTACGGGAGGCAGCAGTGGGGAATATTGGACAATGGGCGAAAG
CCTG-ATCCAGCCATGCCGCGTGTGTGAAGAAGGCCTTATGG-
TTGTAAAGCACTTTTAAGTGGGGAGAAAAGCTTATGGATAACACCCATAGGTTCTG
-ACGTT-ACCCACAGAATAAGCACCGGCTAACTCTGTGCC
>Chryseobacterium hominis NF802 (NR042517.2)
GATGAACGCTAGCGGGAGGCCTAACACATGCAAGCCGAGCGGTATTT----
GTTCTTCGGAACAGAGAGAGCGGCGCACGGGTGCGGAACACGTGTGCAACCTGCC
TTTATCTGGGGGATAGCCTTTCGAAAGGAAGATTAATACCCCATAATATATTGAGT
GGCATCATTTGATATTGAAAACTCCGGTGGATAGAGATGGGCACGCGCAAGATTA
GATAGTTGGTGAGGTAACGGCTCACCAAGTCAATGATCTTTAGGGGGCCTGAGAG
GGTGATCCCCCACACTGGTACTGAGACACGGACCAGACTCCTACGGGAGGCAGCA
GTGAGGAATATTGGACAATGGGTTAGCGCCTG-

ATCCAGCCATCCCGCGTGAAGGACGACGGCCCTATGGGTTGTAAACTTCTTTTGTA
TAGGGATAAACCTACTCTCGTGAGA-------GTAGCTGAAGGT-
ACTATACGAATAAGCACCGGCTAACTCCGTGCC
>Chryseobacterium hagamense RHA2-9 (NR115852)
GATGAACGCTAGCGGGAGGCCTAACACATGCAAGCTGAGCGGTATTT----
GTCTTTCGGGACAGAGAGAGCGGCGTACGGGTGCGGAACACGTGTGCAACCTGCC
TTTATCTGGGGGATAGCCTTTCGAAAGGAAGATTAATACCCCATAACATATTGAGT
GGCATCACTTGATATTGAAAACTCCGGTGGATAGAGATGGGCACGCGCAAGATTA
GATAGTTGGTGAGGTAACGGCTCACCAAGTCGATGATCTTTAGGGGGCCTGAGAG
GGTGATCCCCCACACTGGTACTGAGACACGGACCAGACTCCTACGGGAGGCAGCA
GTGAGGAATATTGGACAATGGGTTAGCGCCTG-
ATCCAGCCATCCCGCGTGAAGGACGACGGCCCTATGGGTTGTAAACTTCTTTTGTA
CAGGGATAAACCTACCCTCGTGAGG-------GTAGCTGAAGGT-
ACTGTACGAATAAGCACCGGCTAACTCCGTGCC
>Chryseobacterium bovis H9 (NR044166)
GATGAACGCTAGCGGGAGGCCTAACACATGCAAGCCGAGCGGTAGAG----
TATCTTCGGATACTTGAGAGCGGCGCACGGGTGCGGAACACGTGTGCAACCTGCCT
TTATCTGGGGGATAGCCTTTCGAAAGGAAGATTAATACCCCATAATATATTTGATG
GCATCATTAGATATTGAAAACTCCGGTGGATAGAGATGGGCACGCGCAAGATTAG
TTAGTTGGTGAGGTAACGGCTCACCAAGGCGATGATCTTTAGGGGGCCTGAGAGG
GTGATCCCCCACACTGGTACTGAGACACGGACCAGACTCCTACGGGAGGCAGCAG
TGAGGAATATTGGACAATGGGTTAGCGCCTG-
ATCCAGCCATCCCGCGTGAAGGACGACGGCCCTATGGGTTGTAAACTTCTTTTGTA
TAGGGATAAACCTACTCTCGTGAGA-------GTAGCTGAAGGT-
ACTATACGAATAAGCACCGGCTAACTCCGTGCC
>Chryseobacterium proteolyticum 9670 (NR112113)
GATGAACGCTAGCGGGAGGCCTAACACATGCAAGCCGAGCGGTATAG----
ATCTTTCGGGATCTAGAGAGCGGCGCACGGGTGCGGAACACGTGTGCAACCTACCT
TTATCAGGGGGATAGCCTTTCGAAAGGAAGATTAATACCCCATAATATATTTGATG
GCATCATTAGATATTGAAAACTCCGGTGGATAGAGATGGGCACGCGCAAGATTAG
ATAGTTGGTGAGGTAACGGCTCACCAAGTCAATGATCTTTAGGGGGCCTGAGAGG
GTGATCCCCCACACTGGTACTGAGACACGGACCAGACTCCTACGGGAGGCAGCAG
TGAGGAATATTGGACAATGGGTGAGAGCCTG-
ATCCAGCCATCCCGCGTGAAGGATGACGGCCCTATGGGTTGTAAACTTCTTTTGTA
TAGGGATAAACCTACCCTCGTGAGG-------GTAGCTGAAGGT-
ACTATACGAATAAGCACCGGCTAACTCCGTGCC
>Chryseobacterium arthrosphaerae CC-VM-7 (NR116977)
GATGAACGCTAGCGGGAGGCCTAACACATGCAAGCCGAGCGGTAGAG----
ATCTTTCGGGATCTTGAGAGCGGCGTACGGGTGCGGAACACGTGTGCAACCTGCCT
TTATCAGGGGGATAGCCTTTCGAAAGGAAGATTAATACCCCATAATATATCGAGTG
GCATCACTTGATATTGAAAACTCCGGTGGATAGAGATGGGCACGCGCAAGATTAG
ATAGTTGGTGAGGTAACGGCTCACCAAGTCTGCGATCTTTAGGGGGCCTGAGAGGG
TGATCCCCCACACTGGTACTGAGACACGGACCAGACTCCTACGGGAGGCAGCAGT
GAGGAATATTGGACAATGGGTGCGAGCCTG-
ATCCAGCCATCCCGCGTGAAGGATGACGGCCCTATGGGTTGTAAACTTCTTTTGTA
TAGGGATAAACCTACCCTCGTGAGG-------GTAGCTGAAGGT-
ACTATACGAATAAGCACCGGCTAACTCCGTGCC
>Acinetobacter johnsonii ATCC 17909 (NR117624)
ATTGAACGCTGGCGGCAGGCTTAACACATGCAAGTCGAGCGGGGAAA-
GGTAGCTTGCTACCT-AACCTAGCGGCGGACGGGTGAGTAATGCTTAGG-
AATCTGCCTATTAGTGGGGGACAACATTCCGAAAGGAATGCTAATACCGCATA-

CGCCCTACGGGGGAAAGCAGGGGATCTTCGGACCTTGCGCTAATAGATGAGCCTA
AGTCAGATTAGCTAGTTGGTGGGGTAAAGGCCTACCAAGGCGACGATCTGTAGCG
GGTCTGAGAGGATGATCCGCCACACTGGGACTGAGACACGGCCCAGACTCCTACG
GGAGGCAGCAGTGGGGAATATTGGACAATGGGCGAAAGCCTG-
ATCCAGCCATGCCGCGTGTGTGAAGAAGGCCTTTTGG-
TTGTAAAGCACTTTAAGCGAGGAGGAGGCTACTTGGATTAATACTCTAGGATAGTG
GACGTT-ACTCGCAGAATAAGCACCGGCTAACTCTGTGCC
>Acinetobacter johnsonii Mannheim 3865/60 (NR044975)
ATTGAACGCTGGCGGCAGGCTTAACACATGCAAGTCGAGCGGGGAGA-
GGTAGCTTGCTACCT-AACCTAGCGGCGGACGGGTGAGTAATGCTTAGG-
AATCTGCCTATTAGTGGGGGACAACATTCCGAAAGGAATGCTAATACCGCATA-
CGTCCTACGGGGGAAAGCAGGGGATCTTCGGACCTTGCGCTAATAGATGAGCCTA
AGTCAGATTAGCTAGTTGGTGGGGTAAAGGCCTACCAAGGCGACGATCTGTAGCG
GGTCTGAGAGGATGATCCGCCACACTGGGACTGAGACACGGCCCAGACTCCTACG
GGAGGCAGCAGTGGGGAATATTGGACAATGGGCGGAAGCCTG-
ATCCAGCCATGCCGCGTGTGTGAAGAAGGCCTTTTGG-
TTGTAAAGCACTTTAAGCGAGGAGGAGGCTACTTGGATTAATACTCTAGGATAGTG
GACGTT-ACTCGCAGAATAAGCACCGGCTAACTCTGTGCC
>Acinetobacter haemolyticus ATCC 17906 (NR117622)
ATTGAACGCTGGCGGCAGGCTTAACACATGCAAGTCGAGCGGGGAAG-
GGTAGCTTGCTACCT-AACCTAGCGGCGGACGGGTGAGTAATGCTTAGG-
AATCTGCCTATTAGTGGGGGACAACATTCCGAAAGGAATGCTAATACCGCATA-
CGTCCTACGGGAGAAAGCAGGGGATCTTCGGACCTTGCGCTAATAGATGAGCCTA
AGTCGGATTAGCTAGTTGGTGGGGTAAAGGCCTACCAAGGCGACGATCTGTAGCG
GGTCTGAGAGGATGATCCGCCACACTGGGACTGAGACACGGCCCAGACTCCTACG
GGAGGCAGCAGTGGGGAATATTGGACAATGGGCGGAAGCCTG-
ATCCAGCCATGCCGCGTGTGTGAAGAAGGCCTTTTGG-
TTGTAAAGCACTTTAAGCGAGGAGGAGGCTACTCTAGTTAATACCTAGAGATAGTG
GACGTT-ACTCGCAGAATAAGCACCGGCTAACTCTGTGCC
>Acinetobacter oleivorans DR1 (NR102814)
ATTGAACGCTGGCGGCAGGCTTAACACATGCAAGTCGAGCGGAGAGA-
GGTAGCTTGCTACTG-ATCTTAGCGGCGGACGGGTGAGTAATGCTTAGG-
AATCTGCCTATTAGTGGGGGACAACATTTCGAAAGGAATGCTAATACCGCATA-
CGTCCTACGGGAGAAAGCAGGGGATCTTCGGACCTTGCGCTAATAGATGAGCCTA
AGTCGGATTAGCTAGTTGGTGGGGTAAAGGCCTACCAAGGCGACGATCTGTAGCG
GGTCTGAGAGGATGATCCGCCACACTGGGACTGAGACACGGCCCAGACTCCTACG
GGAGGCAGCAGTGGGGAATATTGGACAATGGGCGGAAGCCTG-
ATCCAGCCATGCCGCGTGTGTGAAGAAGGCCTTATGG-
TTGTAAAGCACTTTAAGCGAGGAGGAGGCTACTTTAGTTAATACCTAGAGATAGTG
GACGTT-ACTCGCAGAATAAGCACCGGCTAACTCTGTGCC
>Acinetobacter pittii ATCC 19004 (NR117621)
ATTGAACGCTGGCGGCAGGCTTAACACATGCAAGTCGAGCGGAGAGA-
GGTAGCTTGCTACTG-ATCTTAGCGGCGGACGGGTGAGTAATGCTTAGG-
AATCTGCCTATTAGTGGGGGACAACATTTCGAAAGGAATGCTAATACCGCATA-
CGTCCTACGGGAGAAAGCAGGGGATCTTCGGACCTTGCGCTAATAGATGAGCCTA
AGTCGGATTAGCTAGTTGGTGGGGTAAAGGCCTACCAAGGCGACGATCTGTAGCG
GGTCTGAGAGGATGATCCGCCACACTGGGACTGAGACACGGCCCAGACTCCTACG
GGAGGCAGCAGTGGGGAATATTGGACAATGGGCGCAAGCCTG-
ATCCAGCCATGCCGCGTGTGTGAAGAAGGCCTTATGG-
TTGTAAAGCACTTTAAGCGAGGAGGAGGCTACTTTAGTTAATACCTAGAGATAGTG
GACGTT-ACTCGCAGAATAAGCACCGGCTAACTCTGTGCC

>Acinetobacter baumannii DSM 30007 (NR117677)
ATTGAACGCTGGCGGCAGGCTTAACACATGCAAGTCGAGCGGGGGAA-
GGTAGCTTGCTACCG-GACCTAGCGGCGGACGGGTGAGTAATGCTTAGG-
AATCTGCCTATTAGTGGGGGACAACATCTCGAAAGGGATGCTAATACCGCATA-
CGTCCTACGGGAGAAAGCAGGGGATCTTCGGACCTTGCGCTAATAGATGAGCCTA
AGTCGGATTAGCTAGTTGGTGGGGTAAAGGCCTACCAAGGCGACGATCTGTAGCG
GGTCTGAGAGGATGATCCGCCACACTGGGACTGAGACACGGCCCAGACTCCTACG
GGAGGCAGCAGTGGGGAATATTGGACAATGGGGGGAACCCTG-
ATCCAGCCATGCCGCGTGTGTGAAGAAGGCCTTATGG-
TTGTAAAGCACTTTAAGCGAGGAGGAGGCTACTTTAGTTAATACCTAGAGATAGTG
GACGTT-ACTCGCAGAATAAGCACCGGCTAACTCTGTGCC
>Acinetobacter junii Mannheim 2723/59 (NR119360)
ATTGAACGCTGGCGGCAGGCTTAACACATGCAAGTCGAGCGGAGATG-
AGGTGCTTGCACCTT-ATCTTAGCGGCGGACGGGTGAGTAATGCTTAGG-
AATCTGCCTATTAGTGGGGGACAACATTCCGAAAGGAATGCTAATACCGCATA-
CGTCCTACGGGAGAAAGCAGGGGATCTTCGGACCTTGCGCTAATAGATGAGCCTA
AGTCGGATTAGCTAGTTGGTGGGGTAAAGGCCTACCAAGGCGACGATCTGTAGCG
GGTCTGAGAGGATGATCCGCCACACTGGGACTGAGACACGGCCCAGACTCCTACG
GGAGGCAGCAGTGGGGAATATTGGACAATGGGGGGAACCCTG-
ATCCAGCCATGCCGCGTGTGTGAAGAAGGCCTTATGG-
TTGTAAAGCACTTTAAGCGAGGAGGAGGCTACTGAGACTAATACTCTTGGATAGTG
GACGTT-ACTCGCAGAATAAGCACCGGCTAACTCTGTGCC
>Acinetobacter calcoaceticus ATCC 23055 (NR117619)
ATTGAACGCTGGCGGCAGGCTTAACACATGCAAGTCGAGCGGAGTGA-
TGGTGCTTGCACTAT-CACTTAGCGGCGGACGGGTGAGTAATGCTTAGG-
AATCTGCCTATTAGTGGGGGACAACATTTCGAAAGGAATGCTAATACCGCATA-
CGTCCTACGGGAGAAAGCAGGGGATCTTCGGACCTTGCGCTAATAGATGAGCCTA
AGTCGGATTAGCTAGTTGGTGGGGTAAAGGCCTACCAAGGCGACGATCTGTAGCG
GGTCTGAGAGGATGATCCGCCACACTGGGACTGAGACACGGCCCAGACTCCTACG
GGAGGCAGCAGTGGGGAATATTGGACAATGGGCGCAAGCCTG-
ATCCAGCCATGCCGCGTGTGTGAAGAAGGCCTTATGG-
TTGTAAAGCACTTTAAGCGAGGAGGAGGCTACTGAAGTTAATACCTTCAGATAGTG
GACGTT-ACTCGCAGAATAAGCACCGGCTAACTCTGTGCC
>Acinetobacter septicus AK001 (NR116071)
ATTGAACGCTGGCGGCAGGCTTAACACATGCAAGTCGAGCGGAGATG-
AGGTGCTTGCACCTT-ATCTTAGCGGCGGACGGGTGAGTAATACTTAGG-
AATCTGGCTATTAGTGGGGGACAACATTTCGAAAGGGATGCTAATACCGCATA-
CGTCCTACGGGAGAAAGCAGGGGATCTTCGGACCTTGCGCTAATAGATGAGCCTA
AGTCGGATTAGCTAGTTGGTGGGGTAAAGGCCTACCAAGGCGACGATCTGTAGCG
GGTCTGAGAGGATGATCCGCCACACTGGGACTGAGACACGGCCCAGACTCCTACG
GGAGGCAGCAGTGGGGAATATTGGACAATGGGGGGAACCCTG-
ATCCAGCCATGCCGCGTGTGTGAAGAAGGCCTTATGG-
TTGTAAAGCACTTTAAGCGAGGAGGAGGGTACTGGTATTAATACTACCAGCTACTG
GACGTT-ACTCGCAGAATAAGCACCGGCTAACTCTGTGCC
>Brevundimonas diminuta NBRC 12697 (NR113602)
AGCGAACGCTGGCGGCAGGCCTAACACATGCAAGTCGAACGGA--------------
CCCTTCGGGGTTAGTGGCGGACGGGTGAGTAACACGTGGG-
AACGTGCCTTTAGGTTCGGAATAGCTCCTGGAAACGGGTGGTAATGCCGAATG-
TGCCCTTCGGGGGAAAG--------------
ATTTATCGCCTTTAGAGCGGCCCGCGTCTGATTAGCTAGTTGGTGAGGTAACGGCT
CACCAAGGCGACGATCAGTAGCTGGTCTGAGAGGATGACCAGCCACACTGGGACT

GAGACACGGCCCAGACTCCTACGGGAGGCAGCAGTGGGGAATCTTGCGCAATGGG
CGAAAGCCTG-ACGCAGCCATGCCGCGTGAATGATGAAGGTCTTAGGA-
TTGTAAAATTCTTTCACCGGGGACGA------------TAATG--------------ACGGT-
ACCCGGAGAAGAAGCCCCGGCTAACTTCGTGCC
>Brevundimonas diminuta LMG 2089 (NR114708)
AGCGAACGCTGGCGGCAGGCCTAACACATGCAAGTCGAACGGA-------------
CCCTTCGGGGTTAGTGGCGGACGGGTGAGTAACACGTGGG-
AACGTGCCTTTAGGTTCGGAATAGCTCCTGGAAACGGGTGGTAATGCCGAATG-
TGCCCTTCGGGGGAAAG-------------
ATTTATCGCCTTTAGAGCGGCCCGCGTCTGATTAGCTAGTTGGTGAGGTAACGGCT
CACCAAGGCGACGATCAGTAGCTGGTCTGAGAGGATGACCAGCCACACTGGGACT
GAGACACGGCCCAGACTCCTACGGGAGGCAGCAGTGGGGAATCTTGCGCAATGGG
CGAAAGCCTG-ACGCAGCCATGCGGCGTGAATGATGAAGGTCTTAGGA-
TTGTAAAATTCTTTCACCGGGGACGA------------TAATG--------------ACGGT-
ACCCGGAGAAGAAGCCCCGGCTAACTTCGTGCC
>Brevundimonas staleyi FWC43 (NR114710)
AGCGAACGCTGGCGGCAGGCCTAACACATGCAAGTCGAACGGA-------------
CCCTTCGGGGTTAGTGGCGGACGGGTGAGTAACACGTGGG-
AACGTGCCTTTAGGTTCGGAATAGCTCCTGGAAACGGGTGGTAATGCCGAATG-
TGCCCTTCGGGGGAAAG-------------
ATTTATCGCCTTTAGAGCGGCCCGCGTCTGATTAGCTAGTTGGTGAGGTAAAGGCT
CACCAAGGCGACGATCAGTAGCTGGTCTGAGAGGATGACCAGCCACATTGGGACT
GAGACACGGCCCAAACTCCTACGGGAGGCAGCAGTGGGGAATCTTGCGCAATGGG
CGAAAGCCTG-ACGCAGCCATGCCGCGTGAATGATGAAGGTCTTAGGA-
TTGTAAAATTCTTTCACCGGGGACGA------------TAATG--------------ACGGT-
ACCCGGAGAAGAAGCCCCGGCTAACTTCGTGCC
>Brevundimonas bullata IAM 13153 (NR025831)
AGCGAACGCTGGCGGCAGGCCTAACACATGCAAGTCGAACGGA-------------
CCCTTCGGGGTTAGTGGCGGACGGGTGAGTAACACGTGGG-
AACGTGCCTTTAGGTTCGGAATAGCTCCTGGAAACGGGTGGTAATGCCGAATG-
TGCCCTTCGGGGGAAAG-------------
ATTTATCGCCTTTAGAGCGGCCCGCGTCTGATTAGCTAGTTGGTGAGGTAATGGCT
CACCAAGGCGACGATCAGTAGCTGGTCTGAGAGGATGACCAGCCACATTGGGACT
GAGACACGGCCCAAACTCCTACGGGAGGCAGCAGTGGGGAATCTTGCGCAATGGG
CGAAAGCCTG-ACGCAGCCATGCCGCGTGAATGATGAAGGTCTTAGGA-
TTGTAAAATTCTTTCACCGGGGACGA------------TAATG--------------ACGGT-
ACCCGGAGAAGAAGCCCCGGCTAACTTCGTGCC
>Brevundimonas abyssalis TAR-001 (NR114308)
AGCGAACGCTGGCGGCAGGCCTAACACATGCAAGTCGAACGGA-------------
CCCTTCGGGGTTAGTGGCGGACGGGTGAGTAACACGTGGG-
AACGTGCCTTTAGGTTCGGAATAAGCCCGGGAAACTGGGTCTAATACCGAATG-
TGCCCTTCGGGGGAAAG-------------
ATTTATCGCCTTTAGAGCGGCCCGCGTCTGATTAGCTAGTTGGTGAGGTAAAGGCT
CACCAAGGCGACGATCAGTAGCTGGTCTGAGAGGATGATCAGCCACACTGGGACT
GAGACACGGCCCAGACTCCTACGGGAGGCAGCAGTGGGGAATCTTGCGCAATGGG
CGAAAGCCTG-ACGCAGCCATGCCGCGTGAATGATGAAGGTCTTAGGA-
TTGTAAAATTCTTTCACCGGGGACGA------------TAATG--------------ACGGT-
ACCCGGAGAAGAAGCCCCGGCTAACTTCGTGCC
>Brevundimonas lenta DS-18 (NR044186)
AGCGAACGCTGGCGGCAGGCCTAACACATGCAAGTCGAACGGA-------------
CCTTTCGGGGTTAGTGGCGGACGGGTGAGTAACACGTGGG-

AACGTGCCTTTAGGTTCGGAATAGCTCCTGGAAACGGGTGGTAATGCCGAATG-
TGCCCTTCGGGGGAAAG--------------
ATTTATCGCCTTTAGAGCGGCCCGCGTCTGATTAGCTAGTTGGTGAGGTAAAGGCT
CACCAAGGCTACGATCAGTAGCTGGTCTGAGAGGATGACCAGCCACATTGGGACT
GAGACACGGCCCAAACTCCTACGGGAGGCAGCAGTGGGGAATCTTGCGCAATGGG
CGAAAGCCTG-ACGCAGCCATGCCGCGTGGATGATGAAGGTCTTAGGA-
TTGTAAAATCCTTTCACCGGTGAAGA------------TAATG--------------ACTGT-
AGCCGGAGAAGAAGCCCCGGCTAACTTCGTGCC
>Acinetobacter ursingii DSM 16037(AIEA01000080)
ATTGAACGCTGGCGGCAGGCTTAACACATGCAAGTCGAGCGGAGAGA-
GGTAGCTTGCTACTG-ATCTTAGCGGCGGACGGGTGAGTAATACTTAGG-
AATCTGGCTATTAGTGGGGGACAACATTTCGAAAGGGATGCTAATACCGCATA-
CGTCCTACGGGAGAAAGCAGGGGATCTTCGGACCTTGCGCTAATAGATGAGCCTA
AGTCGGATTAGCTAGTTGGTGGGGTAAAGGCCTACCAAGGCGACGATCTGTAGCG
GGTCTGAGAGGATGATCCGCCACACTGGGACTGAGACACGGCCCAGACTCCTACG
GGAGGCAGCAGTGGGGAATATTGGACAATGGGGGGAACCCTG-
ATCCAGCCATGCCGCGTGTGTGAAGAAGGCCTTATGG-
TTGTAAAGCACTTTAAGCGAGGAGGAGGGTACTGGTATTAATACTACCAGGTACTG
GACGTT-ACTCGCAGAATAAGCACCGGCTAACTCTGTGCC
>Acinetobacter seifertii LUH 1472 (NR134684)
ATTGAACGCTGGCGGCAGGCTTAACACATGCAAGTCGAGCGGAGAGA-
GGTAGCTTGCTACTG-ATCTTAGCGGCGGACGGGTGAGTAATGCTTAGG-
AATCTGCCTATTAGTGGGGGACAACATCTCGAAAGGGATGCTAATACCGCATA-
CGTCCTACGGGAGAAAGCAGGGGATCTTCGGACCTTGCGCTAATAGATGAGCCTA
AGTCGGATTAGCTAGTTGGTGGGGTAAAGGCCTACCAAGGCGACGATCTGTAGCG
GGTCTGAGAGGATGATCCGCCACACTGGGACTGAGACACGGCCCAGACTCCTACG
GGAGGCAGCAGTGGGGAATATTGGACAATGGGGGGAACCCTG-
ATCCAGCCATGCCGCGTGTGTGAAGAAGGCCTTATGG-
TTGTAAAGCACTTTAAGCGAGGAGGAGGCTACTTTAGTTAATACCTAGAGATAGTG
GACGTT-ACTCGCAGAATAAGCACCGGCTAACTCTGTGCC
>Pseudomonas parafulva NBRC 16636 (NR_113856)
ATTGAACGCTGGCGGCAGGCCTAACACATGCAAGTCGAGCGGATGAC-
GGGAGCTTGCTCCTT-GATTCAGCGGCGGACGGGTGAGTAATGCCTAGG-
AATCTGCCTGGTAGTGGGGGACAACGTTTCGAAAGGAACGCTAATACCGCATA-
CGTCCTACGGGAGAAAGCAGGGGACCTTCGGGCCTTGCGCTATCAGATGAGCCTA
GGTCGGATTAGCTTGTTGGTGAGGTAATGGCTCACCAAGGCGACGATCCGTAACTG
GTCTGAGAGGATGATCAGTCACACTGGAACTGAGACACGGTCCAGACTCCTACGG
GAGGCAGCAGTGGGGAATATTGGACAATGGGCGAAAGCCTG-
ATCCAGCCATGCCGCGTGTGTGAAGAAGGTCTTCGGA-
TTGTAAAGCACTTTAAGTTGGGAGGAAGGGTTGTAGATTAATACTCTGCAATTTTG-
ACGTT-ACCGACAGAATAAGCACCGGCTAACTCTGTGCC
>Pseudomonas cremoricolorata NBRC 16634 (NR_113855)
ATTGAACGCTGGCGGCAGGCCTAACACATGCAAGTCGAGCGGATGAC-
AGGAGCTTGCTCCTG-GATTCAGCGGCGGACGGGTGAGTAATGCCTAGG-
AATCTGCCTGGTAGTGGGGGACAACGTTTCGAAAGGAACGCTAATACCGCATA-
CGTCCTACGGGAGAAAGCAGGGGACCTTCGGGCCTTGCGCTATCAGATGAGCCTA
GGTCGGATTAGCTTGTTGGTGAGGTAATGGCTCACCAAGGCGACGATCCGTAACTG
GTCTGAGAGGATGATCAGTCACACTGGAACTGAGACACGGTCCAGACTCCTACGG
GAGGCAGCAGTGGGGAATATTGGACAATGGGCGAAAGCCTG-
ATCCAGCCATGCCGCGTGTGTGAAGAAGGTCTTCGGA-

TTGTAAAGCACTTTAAGTTGGGAGGAAGGGTTGTAGATTAATACTCTGCAATTTTG-
ACGTT-ACCGACAGAATAAGCACCGGCTAACTCTGTGCC
>Pseudomonas parafulva AJ 2129 (NR_040859)
ATTGAACGCTGGCGGCAGGCCTAACACATGCAAGTCGAGCGGATGAC-
GGGAGCTTGCTCCTT-GATTCAGCGGCGGACGGGTGAGTAATGCCTAGG-
AATCTGCCTGGTAGTGGGGGACAACGTTTCGAAAGGAACGCTAATACCGCATA-
CGTCCTACGGGAGAAAGCAGGGGACCTTCGGGCCTTGCGCTATCAGATGAGCCTA
GGTCGGATTAGCTTGTTGGTGAGGTAATGGCTCACCAAGGCGACGATCCGTAACTG
GTCTGAGAGGATGATCAGTCACACTGGAACTGAGACACGGTCCAGACTCCTACGG
GAGGCAGCAGTGGGGAATATTGGACAATGGGCGAAAGCCTG-
ATCCAGCCATGCCGCGTGTGTGAAGAAGGTCTTCGGA-
TTGTAAAGCACTTTAAGTTGGGAGGAAGGGTTGTAGATTAATACTCTGCAATTTTG-
ACGTT-ACCGACAGAATAAGCACCGGCTAACTCTGTGCC
>Pseudomonas fulva IAM1529 (NR_115610)
ATTGAACGCTGGCGGCAGGCCTAACACATGCAAGTCGAGCGGATGAC-
AGGAGCTTGCTCCTG-GATTCAGCGGCGGACGGGTGAGTAATGCCTAGG-
AATCTGCCTGGTAGTGGGGGACAACGTTTCGAAAGGAACGCTAATACCGCATA-
CGTCCTACGGGAGAAAGCAGGGGACCTTCGGGCCTTGCGCTATCAGATGAGCCTA
GGTCGGATTAGCTTGTTGGTGAGGTAATGGCTCACCAAGGCGACGACCTGTAACTG
GTCTGAGAGGATGATCAGTCACACTGGAACTGAGACACGGTCCAGACTCCTACGG
GAGGCAGCAGTGGGGAATATTGGACAATGGGCGAAAGCCTG-
ATCCAGCCATGCCGCGTGTGTGAAGAAGGTCTTCGGA-
TTGTAAAGCACTTTAAGTTGGGAGGAAGGGTTGTAGATTAATACTCTGCAATTTTG-
ACGTT-ACCGACAGAATAAGCACCGGCTAACTCTGTGCC
>Pseudomonas reinekei MT1 (NR_042541)
ATTGAACGCTGGCGGCAGGCCTAACACATGCAAGTCGAGCGGATGAC-
AGGAGCTTGCTCCTG-AATTCAGCGGCGGACGGGTGAGTAATGCCTAGG-
AATCTGCCTGGTAGTGGGGGACAACGTTTCGAAAGGAACGCTAATACCGCATA-
CGTCCTACGGGAGAAAGCAGGGGACCTTCGGGCCTTGCGCTATCAGATGAGCCTA
GGTCGGATTAGCTAGTTGGTGAGGTAATGGCTCACCAAGGCGACGATCCGTAACTG
GTCTGAGAGGATGATCAGTCACACTGGAACTGAGACACGGTCCAGACTCCTACGG
GAGGCAGCAGTGGGGAATATTGGACAATGGGCGAAAGCCTG-
ATCCAGCCATGCCGCGTGTGTGAAGAAGGTCTTCGGA-
TTGTAAAGCACTTTAAGTTGGGAGGAAGGGTTGTAGATTAATACTCTGCAATTTTG-
ACGTT-ACCGACAGAATAAGCACCGGCTAACTCTGTGCC
>Pseudomonas alcaligenes ATCC 14909 (NR_114472)
ATTGAACGCTGGCGGCAGGCCTAACACATGCAAGTCGAGCGGATGAG-
TGGAGCTTGCTCCAT-GATTCAGCGGCGGACGGGTGAGTAATGCCTAGG-
AATCTGCCTGGTAGTGGGGGACAACGTTTCGAAAGGAACGCTAATACCGCATA-
CGTCCTACGGGAGAAAGCAGGGGACCTTCGGGCCTTGCGCTATCAGATGAGCCTA
GGTCGGATTAGCTAGTAGGTGAGGTAATGGCTCACCTAGGCGACGATCCGTAACTG
GTCTGAGAGGATGATCAGTCACACTGGAACTGAGACACGGTCCAGACTCCTACGG
GAGGCAGCAGTGGGGAATATTGGACAATGGGCGAAAGCCTG-
ATCCAGCCATGCCGCGTGTGTGAAGAAGGTCTTCGGA-
TTGTAAAGCACTTTAAGTTGGGAGGAAGGGCAGTAAGTTAATACCTTGCTGTTTTG-
ACGTT-ACCAACAGAATAAGCACCGGCTAACTTCGTGCC
>Chryseobacterium lactis KC1864 (NR_126256)
GATGAACGCTAGCGGGAGGCCTAACACATGCAAGCCGAGCGGTAGAG----
ATTCTTCGGAATCTTGAGAGCGGCGTACGGGTGCGGAACACGTGTGCAACCTGCCT
TTATCTGGGGGATAGCCTTTCGAAAGGAAGATTAATACCCCATAATATATTGAATG
GCATCATTTGATATTGAAAACTCCGGTGGATAGAGATGGGCACGCGCAAGATTAG

ATAGTTGGTGAGGTAACGGCTCACCAAGTCTACGATCTTTAGGGGGCCTGAGAGGG
TGATCCCCCACACTGGTACTGAGACACGGACCAGACTCCTACGGGAGGCAGCAGT
GAGGAATATTGGACAATGGGTGAGAGCCTG-
ATCCAGCCATCCCGCGTGAAGGACGACGGCCCTATGGGTTGTAAACTTCTTTTGTA
TAGGGATAAACCTACTCTCGTGAGA-------GTAGCTGAAGGT-
ACTATACGAATAAGCACCGGCTAACTCCGTGCC
>Acinetobacter pittii CIP 70.29 (NR_116774)
ATTGAACGCTGGCGGCAGGCTTAACACATGCAAGTCGAGCGGAGAGA-
GGTAGCTTGCTACTG-ATCTTAGCGGCGGACGGGTGAGTAATGCTTAGG-
AATCTGCCTATTAGTGGGGGACAACATTTCGAAAGGAATGCTAATACCGCATA-
CGTCCTACGGGAGAAAGCAGGGGATCTTCGGACCTTGCGCTAATAGATGAGCCTA
AGTCGGATTAGCTAGTTGGTGGGGTAAAGGCCTACCAAGGCGACGATCTGTAGCG
GGTCTGAGAGGATGATCCGCCACACTGGGACTGAGACACGGCCCAGACTCCTACG
GGAGGCAGCAGTGGGGAATATTGGACAATGGGCGCAAGCCTG-
ATCCAGCCATGCCGCGTGTGTGAAGAAGGCCTTATGG-
TTGTAAAGCACTTTAAGCGAGGAGGAGGCTACTTTAGTTAATACCTAGAGATAGTG
GACGTT-ACTCGCAGAATAAGCACCGGCTAACTCTGTGCC
>Beijerinckia fluminensis UQM 1685 (Agrobacterium radiobacter)(NR_116306)
AACGAACGCTGGCGGCAGGCTTAACACATGCAAGTCGAACG----------------
CCCCGCAAGGGGAGTGGCAGACGGGTGAGTAACGCGTGGG-
AACATACCCTTTCCTGCGGAATAGCTCCGGGAAACTGGAATTAATACCGCATA-
CGCCCTACGGGGGAAAG--------------
ATTTATCGGGGAAGGATTGGCCCGCGTTGGATTAGCTAGTTGGTGGGGTAAAGGCC
TACCAAGGCGACGATCCATAGCTGGTCTGAGAGGATGATCAGCCACATTGGGACT
GAGACACGGCCCAAACTCCTACGGGAGGCAGCAGTGGGGAATATTGGACAATGGG
CGCAAGCCTG-ATCCAGCCATGCCGCGTGAGTGATGAAGGCCTTAGGG-
TTGTAAAGCTCTTTCACCGGAGAAGA------------TAATG--------------ACGGT-
ATCCGGAGAAGAAGCCCCGGCTAACTTCGTGCC
>Agrobacterium tumefaciens NCPPB2437 (NR_115516)
AACGAACGCTGGCGGCAGGCTTAACACATGCAAGTCGAACG----------------
CCCCGCAAGGGGAGTGGCAGACGGGTGAGTAACGCGTGGG-
AACATACCCTTTCCTGCGGAATAGCTCCGGGAAACTGGAATTAATACCGCATA-
CGCCCTACGGGGGAAAG--------------
ATTTATCGGGGAAGGATTGGCCCGCGTTGGATTAGCTAGTTGGTGGGGTAAAGGCC
TACCAAGGCGACGATCCATAGCTGGTCTGAGAGGATGATCAGCCACATTGGGACT
GAGACACGGCCCAAACTCCTACGGGAGGCAGCAGTGGGGAATATTGGACAATGGG
CGCAAGCCTG-ATCCAGCCATGCCGCGTGAGTGATGAAGGCCTTAGGG-
TTGTAAAGCTCTTTCACCGGAGAAGA------------TAATG--------------ACGGT-
ATCCGGAGAAGAAGCCCCGGCTAACTTCGTGCC
>Agrobacterium larrymoorei AF3.10 (NR_026519)
AACGAACGCTGGCGGCAGGCTTAACACATGCAAGTCGAACG----------------
CATCGCAAGATGAGTGGCAGACGGGTGAGTAACGCGTGGG-
AACATACCCTTTTCTACGGAATAGCTCTGGGAAACTGGAATTAATACCGTATA-
CGCCCTACGGGGGAAAG--------------
ATTTATCGGGGAAGGATTGGCCCGCGTTGGATTAGCTAGTTGGTGGGGTAAAGGCC
TACCAAGGCGACGATCCATAGCTGGTCTGAGAGGATGATCAGCCACATTGGGACT
GAGACACGGCCCAAACTCCTACGGGAGGCAGCAGTGGGGAATATTGGACAATGGG
CGCAAGCCTG-ATCCAGCCATGCCGCGTGAGTGATGAAGGCCTTAGGG-
TTGTAAAGCTCTTTCACCGATGAAGA------------TAATG--------------ACGGT-
AGTCGGAGAAGAAGCCCCGGCTAACTTCGTGCC
>Agrobacterium fabrum C58 (NR_074266)

AACGAACGCTGGCGGCAGGCTTAACACATGCAAGTCGAACG----------------
CCCCGCAAGGGGAGTGGCAGACGGGTGAGTAACGCGTGGG-
AATCTACCCATCTCTGCGGAATAGCTCTGGGAAACTGGAATTAATACCGCATA-
CGCCCTACGGGGGAAAG--------------
ATTTATCGGGGATGGATGAGCCCGCGTTGGATTAGCTAGTTGGTGGGGTAAAGGCC
TACCAAGGCGACGATCCATAGCTGGTCTGAGAGGATGATCAGCCACATTGGGACT
GAGACACGGCCCAAACTCCTACGGGAGGCAGCAGTGGGGAATATTGGACAATGGG
CGCAAGCCTG-ATCCAGCCATGCCGCGTGAGTGATGAAGGCCTTAGGG-
TTGTAAAGCTCTTTCACCGATGAAGA------------TAATG--------------ACGGT-
AGTCGGAGAAGAAGCCCCGGCTAACTTCGTGCC
>Sphingobium yanoikuyae NBRC 15102 (NR_113730)
AACGAACGCTGGCGGCATGCCTAATACATGCAAGTCGAACGAGA--------------
TCTTCGGATCTAGTGGCGCACGGGTGCGTAACGCGTGGG-
AATCTGCCCTTGGGTTCGGAATAACTTCTGGAAACGGAAGCTAATACCGGATG-
ATGACGTAAGTCCAAAG--------------
ATTTATCGCCCAAGGATGAGCCCGCGTAGGATTAGCTAGTTGGTGAGGTAAAGGCT
CACCAAGGCGACGATCCTTAGCTGGTCTGAGAGGATGATCAGCCACACTGGGACT
GAGACACGGCCCAGACTCCTACGGGAGGCAGCAGTAGGGAATATTGGACAATGGG
CGAAAGCCTG-ATCCAGCAATGCCGCGTGAGTGATGAAGGCCTTAGGG-
TTGTAAAGCTCTTTTACCCGGGATGA------------TAATG--------------ACAGT-
ACCGGGAGAATAAGCTCCGGCTAACTCCGTGCC
>Sphingobium yanoikuyae GIFU 9882 (NR_115524)
AACGAACGCTGGCGGCATGCCTAATACATGCAAGTCGAACGAGA--------------
TCTTCGGATCTAGTGGCGCACGGGTGCGTAACGCGTGGG-
AATCTGCCCTTGGGTTCGGAATAACTTCTGGAAACGGAAGCTAATACCGGATG-
ATGACGTAAGTCCAAAG--------------
ATTTATCGCCCAAGGATGAGCCCGCGTAGGATTAGCTAGTTGGTGAGGTAAAGGCT
CACCAAGGCGACGATCCTTAGCTGGTCTGAGAGGATGATCAGCCACACTGGGACT
GAGACACGGCCCAGACTCCTACGGGAGGCAGCAGTAGGGAATATTGGACAATGGG
CGAAAGCCTG-ATCCAGCAATGCCGCGTGAGTGATGAAGGCCTTAGGG-
TTGTAAAGCTCTTTTACCCGGGATGA------------TAATG--------------ACAGT-
ACCGGGAGAATAAGCTCCGGCTAACTCCGTGCC
>Sphingobium yanoikuyae IFO 15102 (NR_036767)
AACGAACGCTGGCGGCATGCCTAATACATGCAAGTCGAACGAGA--------------
TCTTCGGATCGAGTGGCCCACGGGTGCGTAACGCGTGGG-
AATCTGCCCTTGGGTTCGGAATAACTTCTGGAAACGGAAGCTAATACCGGATG-
ATGACGTAGGTCCAAAG--------------
ATTTATCGCCCAAGGATGAGCCCGCGTAGGATTAGCTAGTTGGTGAGGTAAAGGCT
CACCAAGGCGACGATCCTTAGCTGGTCTGAGAGGATGATCAGCCACACTGGGACT
GAGACACGGCCCAGACTCCTACGGGAGGCAGCAGTGGGGAATATTGGACAATGGG
CGAAAGCCTG-ATCCAGCAATGCCGCGTGAGTGATGAAGGCCTTAGGG-
TTGTAAAGCTCTTTTACCCGGGATGA------------TAATG--------------ACAGT-
ACCGGGAGAATAAGCTCCGGCTAACTCCGTGCC
>Sphingobium cloacae NBRC 102517 (NR_114135)
AACGAACGCTGGCGGCATGCCTAATACATGCAAGTCGAACGAGA--------------
TCTTCGGATCTAGTGGCGCACGGGTGCGTAACGCGTGGG-
AATCTGCCCTTGGGTTCGGAATAACATCGGGAAACTGATGCTAATACCGGATG-
ATGACGAAAGTCCAAAG--------------
ATTTATCGCCCAGGGATGAGCCCGCGTAGGATTAGCTAGTTGGTGGGGTAAAGGCT
CACCAAGGCGACGATCCTTAGCTGGTCTGAGAGGATGATCAGCCACACTGGGACT
GAGACACGGCCCAGACTCCTACGGGAGGCAGCAGTAGGGAATATTGGACAATGGG

GGCAACCCTG-ATCCAGCAATGCCGCGTGAGTGATGAAGGCCTTAGGG-
TTGTAAAGCTCTTTTACCCGGGATGA------------TAATG--------------ACAGT-
ACCGGGAGAATAAGCTCCGGCTAACTCCGTGCC
>Sphingobium herbicidovorans NBRC 16415 (NR_113843)
AACGAACGCTGGCGGCATGCCTAATACATGCAAGTCGAACGAGA-------------
TCTTCGGATCTAGTGGCGCACGGGTGCGTAACGCGTGGG-
AATCTGCCCTTGGGTTCGGAATAACAGTTGGAAACGACTGCTAATACCGGATG-
ATGACGTAAGTCCAAAG--------------
ATTTATCGCCCAAGGATGAGCCCGCGTAGGATTAGCTAGTTGGTGAGGTAAAGGCT
CACCAAGGCTACGATCCTTAGCTGGTCTGAGAGGATGATCAGCCACACTGGGACTG
AGACACGGCCCAGACTCCTACGGGAGGCAGCAGTAGGGAATATTGGACAATGGGC
GAAAGCCTG-ATCCAGCAATGCCGCGTGAGTGATGAAGGCCTTAGGG-
TTGTAAAGCTCTTTTACCCGGGATGA------------TAATG--------------ACAGT-
ACCGGGAGAATAAGCCCCGGCTAACTCCGTGCC
>Sphingobium ummariense RL-3 (NR_044171)
AACGAACGCTGGCGGCATGCCTAATACATGCAAGTCGAACGAAG-------------
GCTTCGGCCTTAGTGGCGCACGGGTGCGTAACGCGTGGG-
AATCTGCCCTTGGGTTCGGAATAACGTCTGGAAACGGACGCTAATACCGGATG-
ATGACGTAAGTCCAAAG--------------
ATTTATCGCCCAGGGATGAGCCCGCGTAGGATTAGCTAGTTGGTGAGGTAAAGGCT
CACCAAGGCGACGATCCTTAGCTGGTCTGAGAGGATGATCAGCCACACTGGGACT
GAGACACGGCCCAGACTCCTACGGGAGGCAGCAGTAGGGAATATTGGACAATGGG
CGAAAGCCTG-ATCCAGCAATGCCGCGTGAGTGATGAAGGCCTTAGGG-
TTGTAAAGCTCTTTTACCCGGGATGA------------TAATG--------------ACAGT-
ACCGGGAGAATAAGCCCCGGCTAACTCCGTGCC
